# Conformationally Constrained Phosphonate Backbone Linkages Enhance CpG Oligonucleotide Activation of Toll-Like Receptor 9

**DOI:** 10.1021/acsptsci.6c00326

**Published:** 2026-07-29

**Authors:** Ivana Kóšiová, Ivan Štěpánek, Ondřej Šimák, Ondřej Kostov, Magdalena Petrová, Ondřej Páv, Dominik Rejman, Radek Liboska, Miloš Buděšínský, Šárka Rosenbergová, Gabriel Birkuš, Ivan Rosenberg

**Affiliations:** Institute of Organic Chemistry and Biochemistry, 89220 Czech Academy of Sciences, Flemingovo nám. 2, Prague 6 166 10, Czech Republic

**Keywords:** TLR9, TLR9 agonists, CpG oligonucleotides, phosphonate internucleotide linkages, backbone conformation, solid-phase oligonucleotide synthesis

## Abstract

The effect of flexible versus conformationally constrained phosphonate internucleotide linkages on CpG-mediated TLR9 activation was investigated. Sixteen analogues of the CpG oligonucleotide **ODN 2006**, a well-known TLR9 agonist, uniformly modified at all CpG motifs, were synthesized and evaluated in a human TLR9 (hTLR9) reporter cell line and peripheral blood mononuclear cells. Analogues bearing conformationally constrained 2′,3′-cytidine phosphonate modifications (**ODN1**–**ODN10**) generally exhibited enhanced activity relative to **ODN 2006**, with **ODN1** (incorporating modification **I**) displaying ∼30-fold increased potency. Analogues with flexible phosphonate linkages (**ODN11**, **ODN13**, **ODN15**, **ODN16**) showed comparable activity to that of the parent ODN, whereas additional sugar modification abolished activity (**ODN12**, **ODN14**). A hexamer (**ODN-B**) incorporating the most potent modification **I**, when combined with CpG DNA, enhanced hTLR9 activation, consistent with interaction at the auxiliary 5′-xCx site. This study evaluates phosphonate internucleotide linkages in CpG oligonucleotides and demonstrates that a conformationally constrained phosphonate backbone in **ODN 2006** analogues enhances hTLR9 activation, offering a potential strategy for the development of potent immunostimulatory oligonucleotides.

## Graphical abstract

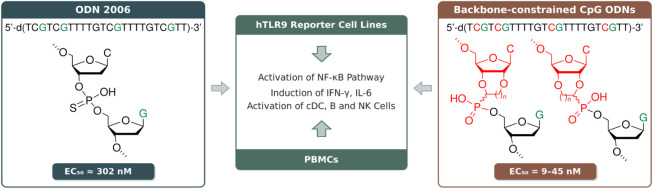

Toll-like receptor 9 (TLR9) is an intracellular pattern recognition receptor that detects bacterial DNA or synthetic oligodeoxynucleotides (ODNs) through interaction with unmethylated CpG motifs,^1–5^ which occur less frequently in vertebrate DNA than would be expected from a random distribution of nucleobases. In humans, TLR9 is mainly expressed in B cells and plasmacytoid dendritic cells (pDCs),^6^ whereas in mice, its expression is broader and includes myeloid immune cells.^3^ The CpG sites are often found in a specific sequence, where they are preceded by C and followed by G (CCpGG), and this pattern does not strongly activate TLR9.^6^ The sequence required for TLR9 activation in vertebrate DNA is described as XCGY, where X is any base except C and Y is any base except G. The optimal sequence is species-specific: GACGTT is among the most potent motifs for murine TLR9 (mTLR9),^7^ whereas GTCGTT is most effective in human cells.^8^ The most effective ODNs typically contain duplicated or triplicated CpG motifs, with maximal activity when motifs are separated by at least two nucleotides.^6^

The effects of ODN backbone, sugar, and nucleobase modifications on TLR9 ligand activity have been reported. Native phosphodiester DNA is rapidly degraded by endonucleases; therefore, CpG ODNs typically use a nuclease-resistant phosphorothioate backbone, extending the *in vivo* half-life from minutes to several days.^6^ Sugar modifications within the CpG motif, including LNA (locked nucleic acid), ENA (ethylene-bridged nucleic acid), or 2′-*O*-methyl and 2′-*O*-methoxymethyl analogues, markedly reduce TLR9 stimulation.^9–12^ Modifications of cytosine or guanine within CpG motifs are generally not well tolerated,^13–16^ although some changes—such as 5-hydroxy or 4-amino-alkyl substitutions of cytidine^14^ or replacement of guanine with hypoxanthine or 6-thioguanine^16^—can retain activity. In contrast, modifications in the flanking sequences are often tolerated and may even enhance activity.^17–19^

Three main classes of immunostimulatory ODNs have been defined based on sequence, structure, backbone (phosphoro-thioate vs phosphodiester), and their effects on PBMCs.^20–24^ Type A ODNs (e.g., human **ODN 2216**) feature a central CpG-containing palindromic phosphodiester core and a fully phosphorothioate-modified 3′ poly dG tail. They elicit robust IFN-α production by pDCs but only weakly activate NF-κB signaling. Type B ODNs (e.g., human **ODN 2006**; mouse **ODN 1826**) possess a phosphorothioate backbone with multiple CpG motifs and induce high levels of IL-6 in PBMCs. They potently activate B cells and natural killer (NK) cells but are poor inducers of IFN-α in pDCs. The CpG C-class combines the characteristics of the A- and B-classes, stimulating strong B cell and pDC type I interferon production and consists of a hexameric CpG motif positioned at or near the 5′-end and linked by a T spacer to a GC-rich palindromic sequence.^15^

Several synthetic ODN agonists of human TLR9 are under development for cancer and other immune-related diseases.^25–27^ Type B **ODN 2006** (CpG 7909, agatolimod), developed by Coley Pharmaceutical Group and licensed to Pfizer, contains a phosphorothioate backbone and the optimal human TLR9-stimulatory motif GTCGTT.^8^ It has been evaluated as a single agent and in combination with other therapies or vaccines in clinical trials for stage IV renal cell carcinoma, melanoma, cutaneous T-cell lymphoma, breast neoplasm, and others.^25^

In recent years, the mechanism of TLR9 activation has been discovered.^28–34^ TLR9 contains two DNA-binding sites, the CpG DNA (primary) binding site and the 5′-xCx DNA (secondary) binding site. Engagement of both sites is required for efficient TLR9 activation. Binding of CpG DNA to the primary site induces weak activation.^28,33^ Subsequent binding of a 5′-xCx DNA to the secondary binding site—although insufficient to activate TLR9 on its own—augments CpG DNA-induced signaling by promoting dimerization of activated TLR9. These studies were performed using equine, bovine, and murine TLR9. The mechanism of human TLR9 activation is thought to be the same, however, it has been reported that human TLR9 requires both motifs for activation whereas murine TLR9 can be activated by CpG DNA only.^29–31^

Our research focuses on the synthesis and biochemical and biological evaluation of isopolar, iso-, and nonisosteric phosphonate analogues of natural nucleotides and oligonucleotides.^35–48^ Unlike phosphodiester linkages, phosphonates can be prepared as regioisomeric pairs (5′-O–P–C-3′ or 5′-C–P–O-3′) and can additionally be introduced in conformationally constrained architectures through the formation of five- or six-membered rings bridging the 2′ and 3′ positions of the sugar moiety. Such structural motifs preserve a DNA-like sugar conformation while introducing defined backbone preorganization, a largely unexplored parameter in TLR9 ligand design. In the present study, we investigated the impact of flexible and conformationally constrained phosphonate modifications introduced within CpG motifs of the well-characterized human type B **ODN 2006** on their ability to activate hTLR9. We selected a representative set of phosphonate modifications: (i) 2′,3′-locked cytidine phosphonates featuring five-membered rings (Figure 1a-i; **I–VI**), (ii) 2′,3′-locked cytidine phosphonates featuring six-membered rings (Figure 1a-ii; **VII–X**), and (iii) acyclic 2′- or 3′-phosphonate analogues (Figure 1a-iii), comprising flexible 2′- and 3′-*O*-methylphosphonates (**XI–XIV**), a 3′-*S*-methylphosphonate (**XV**), and a 3′-*O*-methylphosphonothioate (**XVI**).

**Figure 1:**
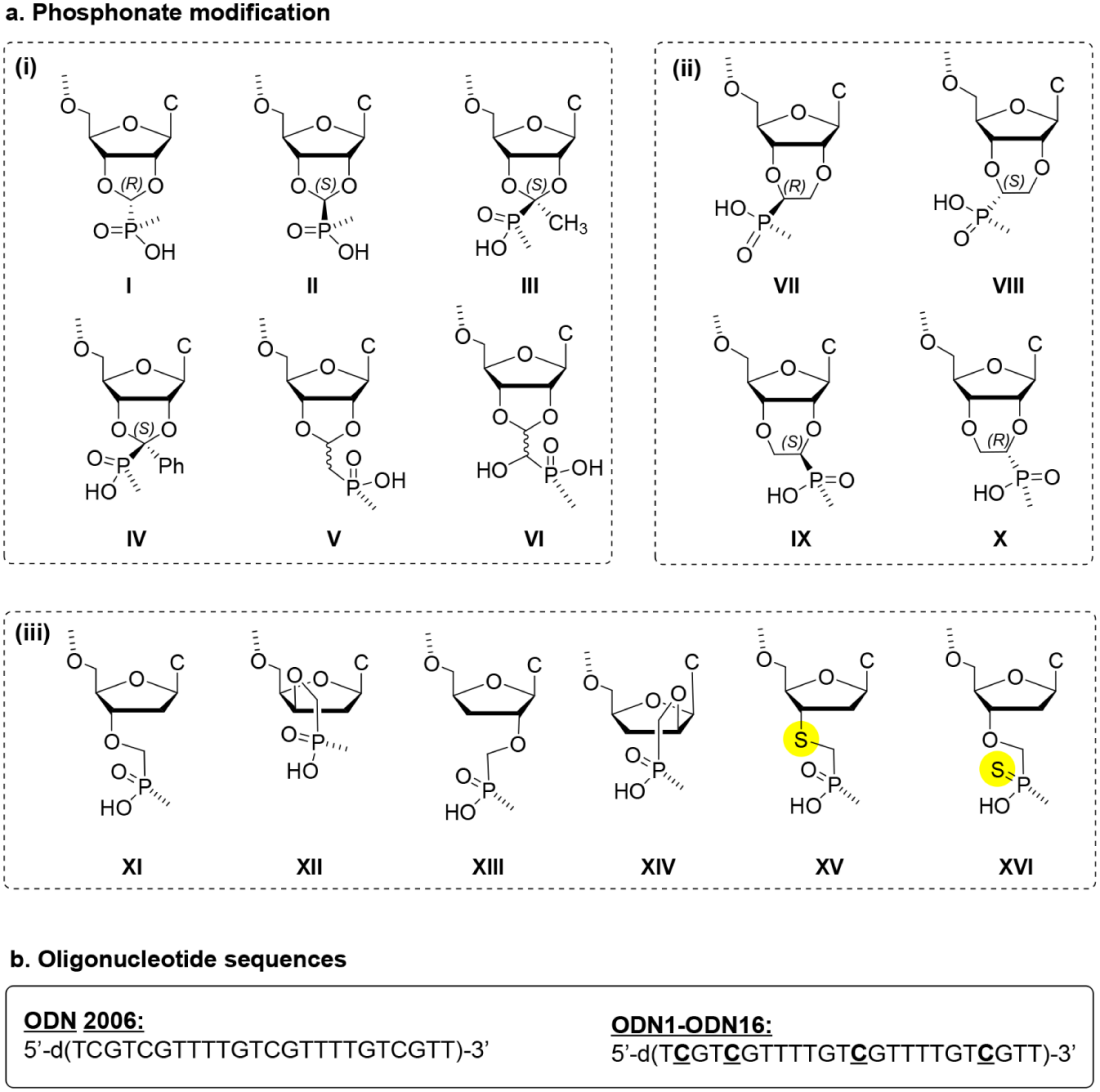
Selected phosphonate modifications for human type B **ODN 2006** analogues (a) Structural classification of phosphonate analogues examined in this study: (i) phosphonate modifications incorporating a five-membered ring bridging the 2′ and 3′ positions; (ii) phosphonate modifications incorporating a six-membered ring bridging the 2′ and 3′ positions; (iii) acyclic phosphonate modifications at the 2′ or 3′ position, including regioisomeric variants and oxygen-to-sulfur substitutions at bridging or nonbridging positions. (b) Oligonucleotide design illustrating the placement of modifications within the model sequence. All oligonucleotides contain phosphorothioate backbone and marked cytidines ( **C** ) correspond with respective modifications (**I-XVI**).

## Results and Discussion

### Design of Phosphonate Modifications

To probe the influence of backbone structure on TLR9 recognition, we designed a panel of phosphonate modifications representing three complementary architectures: conformationally constrained five-membered phosphonate rings, six-membered 1,4-dioxane phosphonates, and flexible acyclic methylphosphonate analogues (Figure 1a). These modifications were selected to modulate backbone preorganization, stereochemistry, and electronic properties of the internucleotide linkage, enabling systematic evaluation of how these parameters affect CpG ODN–mediated activation of human TLR9. The selected architectures were intended to test three distinct structural hypotheses. Conformationally constrained five-membered phosphonate rings were expected to impose strong local backbone preorganization, potentially favoring geometries compatible with TLR9 recognition. Six-membered 1,4-dioxane phosphonate rings introduce a different spatial arrangement of the phosphonate linkage relative to the sugar framework, allowing evaluation of how altered backbone spacing and orientation influence receptor activation. In contrast, acyclic methylphosphonate analogues retain greater conformational flexibility and primarily alter the electronic properties of the internucleotide linkage while minimizing geometric constraints. Together, this set of modifications enables systematic comparison of rigid and flexible phosphonate architectures within CpG motifs of the well-characterized human type B **ODN 2006** scaffold (Figure 1b).

### Synthesis of Modified Oligonucleotides

The studied set of modified oligonucleotides **ODN1–ODN16** was synthesized on solid support using all three condensation chemistries and their mutual combination (Figure 2a). A major part of each sequence, composed of native DNA units, was assembled using standard phosphoramidite chemistry starting from commercially available protected deoxynucleoside 3′-phosphoramidites. To prepare modified sequences **ODN1–ODN15** containing modifications **I–XV**, the corresponding monomers **1–15** (Figure 2b–d) were employed using a combination of phosphoramidite and advanced phosphotriester condensation methodologies.^35,46^ **ODN16**, containing modification **XVI**, was prepared using a combination of phosphoramidite and H-phosphonate condensation chemistry with commercially available protected deoxynucleoside 3′-phosphoramidites and H-phosphinate **16** ^47^ (Figure 2d). Sulfurization after each coupling step afforded phosphorothioate and phosphonothioate linkages.^48^

**Figure 2:**
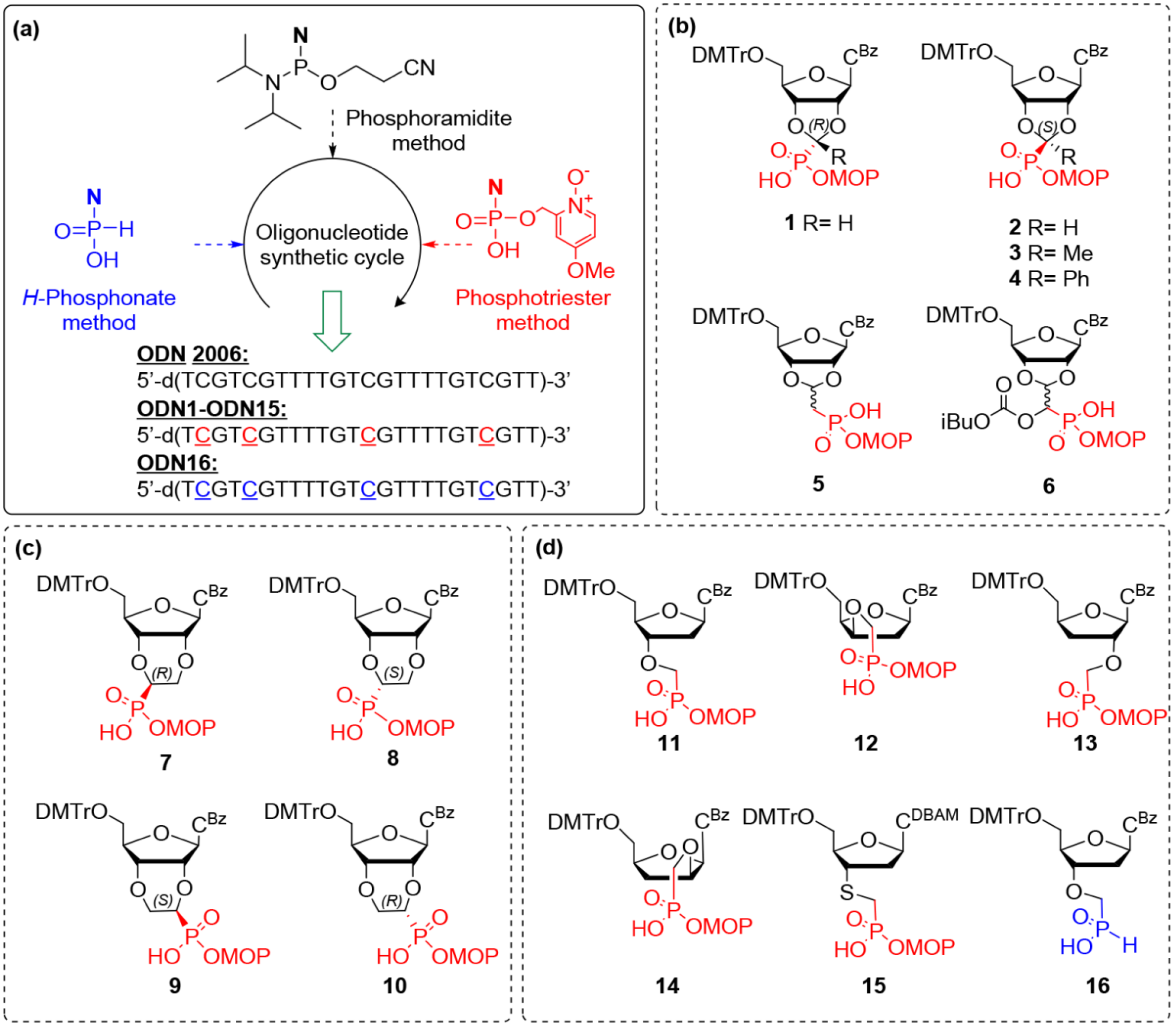
Synthetic methods and monomer units used for the synthesis of **ODN 2006** analogues. a) Schematic representation of the applied oligonucleotide synthesis; b-d) monomer units used in oligonucleotide synthesis; **N** = nucleoside, MOP = 4-hydroxy-1-oxido-2-pyridylmethyl.

This unique combination of three different condensation chemistries was achieved in a continuous solid-phase synthesis using a custom oligonucleotide synthesizer developed in our laboratory, with an upgraded model currently under development for future commercial applications. The preparation of these new oligonucleotide analogues **ODN1–ODN16** was enabled by synthetic methodologies and technological advances established in our laboratory. Detailed protocols for monomer preparation are provided in Supporting Information.

### Modified ODNs in hTLR9 Activation Study

Prepared analogues **ODN1-ODN16** were profiled for their ability to induce human TLR9-dependent expression of secreted embryonic alkaline phosphatase (SEAP) by HEK-Blue hTLR9 cells (InvivoGen), and the induction of IL-6 and INF-γ by human PBMCs (Table 1). We also examined the ability of the ODN analogues to activate immune cell populations present in PBMCs, specifically conventional dendritic cells (cDCs), B cells, and NK cells, by monitoring the expression of activation markers CD80, CD86, and CD69 using flow cytometry (Table 1). The tested analogues were grouped into three structural classes: analogues containing five-membered phosphonate rings (**ODN1-ODN6**), six-membered 1,4-dioxane phosphonate rings (**ODN7-ODN10**), and acyclic methylphosphonate analogues (**ODN11-ODN16**). This classification allowed the comparison of how different backbone architectures influence TLR9 activation and downstream immune responses.

**Table 1:** TLR9 Stimulatory Activity of Analogues of Human Type B ODN 2006^a^

		HEK-Blue cells^b^		PBMC IL-6^b^		PBMC INF-γ^c^		B cells^d^ (CD86)	cDC^d^ (CD80)	NK cells^d^ (CD69)
		EC_50_[nM]	Max	EC_50_[nM]	Max	EC_50_[nM]	Max	EC_50_[nM]	EC_50_[nM]	EC_50_[nM]
**ODN 2006**	-	302 ± 186	1	36 ± 7	1	96 ± 62	1	33 ± 10	19 ± 3	44 ± 8
**ODN1**	**I**	9 ± 6	1.59 ± 0.48	18 ± 17	1.80 ± 0.14	21 ± 16	0.92 ± 0.43	12 ± 6	3 ± 0	15 ± 8
**ODN2**	**II**	38 ± 19	2.22 ± 0.65	34 ± 1	1.39 ± 0.50	41 ± 16	1.09 ± 0.20	17 ± 13	9 ± 2	48 ± 9
**ODN3**	**III**	111 ± 91	1.07 ± 0.21	37 ± 12	0.47 ± 0.10	69 ± 47	1.30 ± 0.84	14 ± 7	21 ± 16	15 ± 8
**ODN4**	**IV**	>1000								
**ODN5**	**V**	213 ± 18	1.38 ± 0.15	31 ± 23	0.57 ± 0.29	51 ± 51	1.19 ± 0.77	14 ± 6	24 ± 22	14 ± 6
**ODN6**	**VI**	241 ± 188	0.90 ± 0.28	41 ± 3	0.53 ± 0.05	68 ± 32	0.85 ± 0.36	16 ± 9	24 ± 10	19 ± 11
**ODN7**	**VII**	14 ± 2	1.45 ± 0.08	22 ± 12	0.68 ± 0.34	24 ± 22	0.88 ± 0.10	9 ± 1	10 ± 1	8 ± 1
**ODN8**	**VIII**	45 ± 13	0.93 ± 0.06	37 ± 1	0.62 ± 0.35	36 ± 6	1.08 ± 0.02	26 ± 5	115 ± 86	62 ± 25
**ODN9**	**IX**	22 ± 1	1.43 ± 0.01	9 ± 1	0.76 ± 0.42	29 ± 5	0.90 ± 0.13	12 ± 4	9 ± 1	13 ± 5
**ODN10**	**X**	166 ± 124	0.97 ± 0.05	39 ± 5	0.65 ± 0.30	40 ± 2	1.11 ± 0.12	16 ± 9	33 ± 21	30 ± 13
**ODN11**	**XI**	159 ± 12	1.45 ± 0.65	22 ± 1	0.91 ± 0.06	38 ± 5	0.70 ± 0.24	16 ± 2	14 ± 3	N.D.
**ODN12**	**XII**	>1000								
**ODN13**	**XIII**	116 ± 52	1.10 ± 0.03	184 ± 34	0.62 ± 0.03	125 ± 102	0.74 ± 0.56	12±3	114 ± 113	249 ± 291
**ODN14**	**XIV**	>1000								
**ODN15**	**XV**	653 ± 217	0.39 ± 0.08	63 ± 36	1.18 ± 0.58	100 ± 21	0.73 ± 0.56	33 ± 2	21 ± 3	N.D.
**ODN16**	**XVI**	301 ± 1	1.03 ± 0.19	13 ± 3	0.89 ± 0.16	45 ± 17	0.73 ± 0.19	19 ± 2	19 ± 3	N.D.

a Max value indicates maximallevel of inducedcytokine/reporter proteinnormalized over maximalcytokine/reporter protein level triggered by **ODN 2006**. EC_50_ represent mean values ± SD. N.D., not determined. The data are results of at least two independent experiments.

b Determined in the HEK-Blue hTLR9 cell reporter cell line.

c Induction of IL-6 and INF-γ by human PBMC treated with ODNs was measured by multiplex ELISA.

d Activation markers on human cDC (CD80), B cells (CD86), and NK cells (CD69) were measured by flow cytometry.

Three analogues from the panel completely lost stimulatory activity. These included **ODN4**, bearing a bulky phenyl substituent, and two acyclic analogues **ODN12** and **ODN14**, both containing phosphonate linkages oriented toward the sugar pocket in the opposite configuration relative to the natural 2′ or 3′ linkage. These results suggest that TLR9 recognition is highly sensitive to bulky hydrophobic substituents in this region and that the stereochemical orientation of the phosphonate linkage relative to the sugar framework is strongly restricted. Analogues containing six-membered 1,4-dioxane phosphonate rings (**ODN7–ODN10**) were generally potent TLR9 agonists, displaying the activity trend **ODN7** > **ODN9** > **ODN8** > **ODN10** in the reporter assay. Notably, **ODN7** and **ODN9** showed strong activity not only in the reporter system but also in PBMC assays, indicating that this class of conformationally constrained phosphonates is well tolerated by the receptor.

Analogues containing flexible acyclic modifications (**XI**, **XIII**, and **XVI**) showed somewhat lower stimulatory activity compared with the constrained phosphonate units but still exhibited activity comparable to that of the parent **ODN 2006**. Replacement of the oxygen atom with sulfur in the 3′-*S-*methylphosphonate unit (**XV**) resulted in a pronounced decrease in the activity of **ODN15**. Replacement of a nonbridging oxygen atom in the phosphonate backbone (**ODN16**) also reduced activity, although to a lesser extent. This observation indicates that substitution at the 2′ and 3′ positions of the sugar can be tolerated provided that the overall sugar configuration and conformation are preserved. However, even subtle heteroatom changes can influence activity.

Among all analogues tested, ODNs containing conformationally constrained modifications **I** and **II** were the most potent stimulators in the TLR9 reporter assay. In particular, **ODN1** containing modification **I** (R-epimer) exhibited the highest potency with an EC_50_ value of 9 ± 6 nM. Analogues bearing a methyl substituent at position 2 of the fused 1,3-dioxolane ring (**ODN3**) and those containing a 2′,3′-phosphonoethylidene linkage (**ODN5** and **ODN6**) showed lower activity compared with **ODN1** and **ODN2**, although their potency remained comparable to that of the **ODN 2006** reference.

The ability of ODN analogues to stimulate B cells, cDCs, and NK cells or to induce cytokine production in PBMCs generally correlated with the results obtained in the hTLR9 reporter assay (Table 1). The most potent agonist, **ODN1**, exhibited 2-fold and 4.5-fold lower EC_50_ values for the induction of IL-6 and IFN-γ, respectively, compared with **ODN 2006**. Furthermore, **ODN1** triggered the expression of CD80, CD86, and CD69 activation markers on cDCs, B cells, and NK cells at 3–7-fold lower EC_50_ concentrations than **ODN 2006**. Analogues **ODN7** and **ODN9** containing modifications **VII** and **IX** also displayed superior activity in PBMC assays relative to **ODN 2006**. In contrast, ODN analogues exhibiting triple-digit nanomolar EC_50_ values in the reporter assay generally induced cytokine production and immune cell activation with potencies similar to those of **ODN 2006**.

### ODN1 Signals via the 5′-xCx Auxiliary Site of Human TLR9

To test if our most potent modification **I** enhances hTLR9 activation via a secondary binding site, we prepared a series of short ODN sequences (Figure 3a). It has been previously determined^29,30^ that at least two CpG motifs within the B-class ODN are required for the activation of hTLR9, while ODNs with a single CpG motif are able to activate only mTLR9. However, later it was shown that mouse-specific ODNs or ODNs with a single CpG motif can trigger an hTLR9 response while being combined with short single-stranded DNA.^31,33^

**Figure 3:**
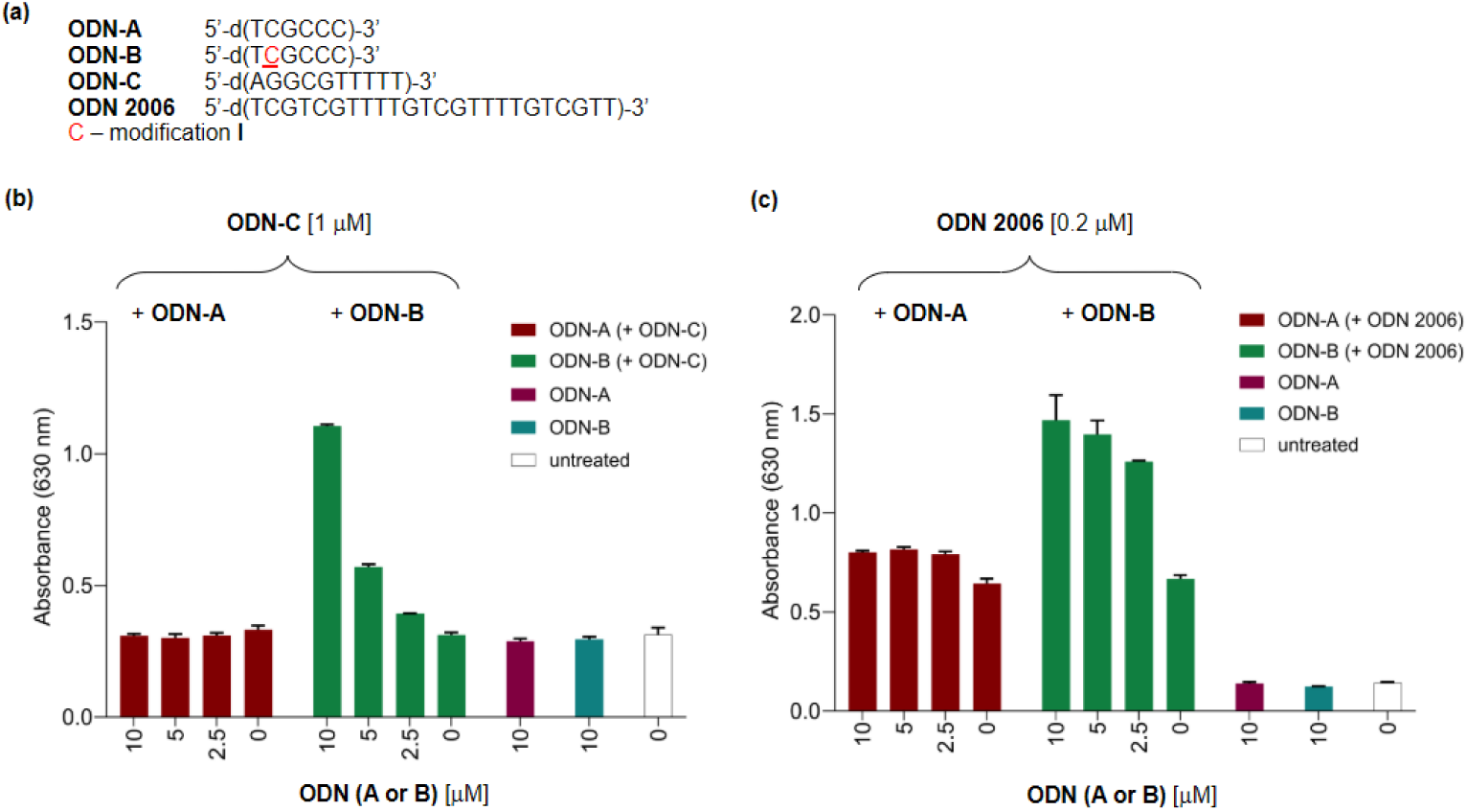
Phosphonate modification **I** enhances the activity of an ODN ligand for the 5′-xCx binding site of hTLR9. a) Sequences of ODNs. b) Hexamer **ODN-B** with phosphonate modification **I** facilitates the hTLR9 activation. c) Hexamer **ODN-B** with phosphonate modification **I** augments the activity of **ODN 2006**. (b, c) The HEK-Blue hTLR9 cell line was stimulated overnight with the combination of the indicated ODNs, and the activity of the NF-κB/AP-1 signaling pathway was recorded by phosphatase activity/absorbance of the substrate. Activities of ODNs were recorded in the concentration range of 10–2.5 μM and plotted as the mean values ± SD. The data shown are representative results of at least two independent experiments.

In our experiment, we used the previously reported^33^ decamer sequence AGGCGTTTTT (**ODN-C**) as a potential ligand for the primary CpG binding site and the hexamer sequence TCGCCC (**ODN-A**) as a potential ligand for the secondary 5′-xCx binding site. As expected, **ODN-C** alone did not activate hTLR9, but its combination with **ODN-A** did not enhance the activity either (Figure 3b). In contrast, incorporation of modification **I** into the hexamer sequence (T **C** GCCC, **ODN-B**) and its subsequent combination with **ODN-C** elicited a measurable response, indicating that this modification contributes to binding at the secondary site (Figure 3b). We next examined the influence of hexamers **ODN-A** and **ODN-B** in combination with **ODN 2006**, which contains both the optimal human TLR9-stimulatory CpG motif and a sequence motif capable of interacting with the secondary binding site within a single strand. The combination of **ODN-A** with **ODN 2006** had no significant effect on hTLR9 activation, whereas **ODN-B** combined with **ODN 2006** enhanced receptor activation (Figure 3c). These results suggest that the hexamer containing modification **I** (**ODN-B**) acts as a more effective ligand for the secondary binding site than the native 5′-xCx motif present in **ODN 2006**. Consequently, **ODN-B** may interact synergistically with **ODN 2006**, leading to enhanced activation of hTLR9. These findings indicate that phosphonate modification **I** not only enhances intrinsic TLR9 agonistic activity but can also improve recognition of ligands targeting the auxiliary 5′-xCx binding site, highlighting the importance of backbone architecture for cooperative TLR9 activation.

### Stability in Human Serum

We have tested the stability of the most active **ODN1**, **ODN2**, and **ODN 2006** in 50% human serum at 37 °C for 20 h. IE HPLC analysis showed full nuclease stability of both parental **ODN 2006** and its analogues.

## Experimental Section

### General Information

Unless otherwise stated, all solvents and reagents were purchased from commercial suppliers, used without further purification and according to the manufacturers’ recommendations. Oligonucleotides were synthesized on an automated GenSyn DNA/RNA synthesizer (IOCB Prague). Following deprotection and cleavage from the solid support, the oligonucleotides were analyzed using a Waters HPLC system equipped with a DNAPac PA100 column (4 × 250 mm; Dionex) at a flow rate of 1 mL/min using appropriate gradients. Detection was performed with a photodiode array (PDA) detector monitoring absorbance at 254 nm. Preparative purification of the oligonucleotides was carried out on a DNAPac PA100 column (10 × 250 mm; Dionex). Purified oligonucleotides were desalted on a Luna C18 column (10 μm, 4 × 30 mm; Phenomenex). Fractions containing the oligonucleotides were concentrated to dryness using a CentriVap concentrator, coevaporated with methanol (3 × 1 mL), and lyophilized from Milli-Q water.

### Synthesis of Monomers

Detailed synthetic procedures for monomer preparation are provided in the Supporting Information (SI).

### Synthesis of Modified Oligonucleotides

Except for **ODN16**, all oligonucleotides were synthesized according to the protocol summarized in Table 2. Solid-phase synthesis was performed in the 3′ → 5′ direction on LCAA-CPG (Glen Research) functionalized with 5′-*O*-dimethoxytrityl-1-(thymin-1-yl)-β-d-ribofuranos-3′-*O*-yl succinate (loading 36 μmol/g), using 30 mg of the solid phase (capacity of 1 μmol) for each synthesis in the automated DNA/RNA synthesizer GenSyn (IOCB Prague). The natural nucleotide units were incorporated into the oligonucleotide chain by the standard phosphoramidite method using commercially available deoxynucleoside phosphoramidites. The phosphonate monomers were introduced into the chain using the advanced phosphotriester method. **ODN16** was prepared according to a previously reported procedure.^48^ All prepared oligonucleotides were characterized by MALDI-TOF mass spectrometry and were obtained with >95% purity as determined by HPLC analysis.

**Table 2:** Protocol for Synthesis of Modified Oligonucleotides **ODN1**-ODN15^a^

**Phosphoramidite coupling protocol**			
Operation	Reagent	Volume [mL]	Time [sec]
1. Detritylation	3% DCA in DCM	3.0	135
2. Coupling	0.1 M nucleoside phosphoramidite in ACN	0.1	600
	0.25 M ETT in ACN	0.1	
3. Sulfurization	0.1 M Reagent *II* in pyridine	0.2	180
4. Capping	Ac_2_O/pyridine/THF 1:1:8	0.1	150
	1-MeIm/THF 1:9	0.1	

**End of the phosphoramidite cycle**			
**Phosphotriester coupling protocol for the incorporation of protected phosphonate monomer**			
Operation	Reagent	Volume [mL]	Time [s]
1. Detritylation	3% DCA in DCM	3	135
2. Coupling	0.1 M monomer in pyridine/ACN 1:1	0.1	600
	0.3 M NEP-Cl in pyridine/ACN 1:9	0.1	
3. Capping	Ac_2_O/pyridine/THF 1:1:8	0.1	150
	1-MeIm/THF 1:9	0.1	

**End of the phosphotriester cycle**			
**Final postsynthetic operations**			
1. Dealkylation of Mop groups	PhSH-Et_3_N-DMF (23:32:45)	0.5	16 h
2. Cleavage and deprotection	Ammonia gas 0.7 bar, 20 °C	—	16 h
3. Ion exchange column chromatography: DNAPac PA100 (Dionex) 10 × 250 mm	0.02 M → 0.5 M NaClO_4_/60 min in 0.050 M Tris-HCl buffer (pH 7.5) containing 10% (v/v) ACN; 3 mL·min^–1^; 80 °C		
4. Desalting on the column Luna C18 (10 μm) 4 × 30 mm	0.05 M triethylammonium bicarbonate → 90% aqueous MeOH/20 min, 2 mL·min^–1^, 20 °C		
5. Adjustment	Lyophilization from water		

a DCA, dichloroacetic acid; DCM, dichloromethane; ACN, acetonitrile; ETT, 5-ethylthiotetrazole; Reagent *II:* DDTT, 3-(dimethylamino-methylidene)­amino)-3*H*-1,2,4-dithiazol-3-thione; Ac_2_O, acetic anhydride; THF, tetrahydrofuran; NEP-Cl, 2-chloro-5,5-dimethyl-1,3,2-dioxaphosphorinane-2-oxide; 1-MeIm, 1-methylimidazole; Mop, 4-hydroxy-1-oxido-2-pyridylmethyl; PhSH, thiophenol; DMF, dimethylformamide.

### ODN Stability in Human Serum

The stability of the most active **ODN1**, **ODN2**, and **ODN 2006** was assessed in 50% human serum at 37 °C for 20 h under the following conditions: 10 μM solution of oligonucleotide in 0.05 M Tris-HCl buffer (pH 7.5) containing 0.01 M magnesium sulfate. Under these conditions, IE HPLC analyses showed complete nuclease stability of both the parental **ODN 2006** and its analogues (for further details, see SI).

### hTLR9 Reporter Assay

HEK-Blue hTLR9 cells (hkb-hTLR9; InvivoGen) were used to assess activation of the hTLR9 signaling pathway according to the manufacturer’s instructions. Briefly, poly-d-lysine–coated 96-well half-area plates (Greiner Bio-One, cat. 675180) were seeded with 40,000 cells per well in 100 μL of complete DMEM supplemented with 10% FBS, 100 U/mL penicillin, and 100 μg/mL streptomycin. After overnight incubation (37 °C, 5% CO_2_), the medium was aspirated, 30 μL of fresh medium was added, followed by 30 μL of 2× concentrated test compound solution prepared in complete medium to achieve final concentrations of 5, 1, 0.2, 0.04, 0.008, and 0.0016 μM. Each concentration was tested in triplicate. After 16 h of incubation, the plates were centrifuged (200 × g, 5 min), and 20 μL of supernatant was transferred to a fresh plate for determination of SEAP activity. In this reporter system, SEAP expression is driven by an IFN-β promoter containing five NF-κB/AP-1 binding sites. SEAP activity was quantified using Quanti-Blue reagent (InvivoGen, rep-qbs) according to the manufacturer’s instructions. Briefly, 20 μL of supernatant was mixed with 180 μL of Quanti-Blue reagent, incubated for 15–45 min at room temperature, and absorbance was measured at 630 nm using a Tecan Spark microplate reader.

Mean absorbance values were plotted against compound concentration in GraphPad Prism 8.4.3 and fitted using a four-parameter logistic (4PL) nonlinear regression with robust fitting. EC_50_ and maximal response (Max) were calculated from the resulting concentration–response curves.

### Activation of PBMCs and Cytokine Production

PBMCs were isolated from buffy coats of healthy donors by density-gradient centrifugation using Ficoll-Paque PLUS (Cytiva, cat. 17144003). PBMCs (1 × 10^6^ cells per well) were seeded into U-bottom 96-well plates (VWR, cat. 734-2228) in 75 μL of complete RPMI 1640 medium supplemented with 10% FBS, 100 U/mL penicillin, and 100 μg/mL streptomycin. Subsequently, 75 μL of 2× concentrated test compound solutions prepared in complete medium were added to achieve final concentrations of 5, 1, 0.2, 0.04, 0.008, and 0.0016 μM. All samples were analyzed in duplicate and compared with unstimulated controls (medium only).

After 16 and 48 h of incubation, the plates were centrifuged (200 × g, 5 min), and the supernatants were collected. The concentrations of IFN-γ and IL-6 were determined using ProcartaPlex Luminex multiplex immunoassays (Human Basic Kit, cat. EPX010-10420-901; IL-6, EPX01A-20603-901; IFN-γ, EPX01A-20606-901; Invitrogen) according to the manufacturer’s instructions. Measurements were performed using a MAGPIX instrument (Millipore).

### Flow Cytometric Analysis of Immune Cell Activation

Activation of cDCs, B cells, and NK cells was analyzed after 48 h of stimulation. Cell pellets were washed with PBS, and dead cells were stained using the Zombie NIR Fixable Viability Kit (BioLegend, cat. 423106) according to the manufacturer’s instructions. Cells were subsequently washed with FC buffer (PBS containing 0.5% BSA and 2 mM EDTA), centrifuged (200 × g, 5 min), and resuspended in antibody mixtures prepared by diluting each antibody 1:100 in FC buffer. Following a 20 min incubation at room temperature in the dark, cells were washed with 150 μL of FC buffer and analyzed using a BD LSRFortessa flow cytometer. Conventional dendritic cells were identified as viable single CD3^–^CD14^–^CD19^–^HLA-DR^+^CD11c^+^ cells, B cells as CD3^–^CD14^–^CD19^+^ cells, and NK cells as CD3^–^CD56^+^ cells. The following antibodies were used: anti-CD3-BV605 (BD Biosciences, cat. 563219), anti-CD14-BV605 (BD Biosciences, cat. 564054), anti-CD19-BUV395 (BD Biosciences, cat. 563549), anti-HLA-DR-PE-CF594 (BD Biosciences, cat. 562331), anti-CD11c-BB515 (BD Biosciences, cat. 564491), anti-CD86-APC (Thermo Fisher Scientific, cat. MHCD8605), anti-CD69-V450 (BD Biosciences, cat. 560740), anti-CD11c-V450 (BD Biosciences, cat. 560370), anti-CD56-FITC (BD Biosciences, cat. 562794), and anti-CD69-APC-R700 (BD Biosciences, cat. 565155). All measurements were performed in at least two independent experiments.

Average cytokine concentrations and the geometric mean fluorescence intensities (GMFI) of the indicated cell populations were plotted against the corresponding concentrations of the tested oligonucleotides using GraphPad Prism 8.4.3. Concentration–response curves were fitted using a four-parameter logistic (4PL) nonlinear regression with robust fitting. EC_50_ values and maximal responses were calculated from the resulting dose–response curves.

## Conclusion

We have long been interested in the synthesis and biochemical as well as biological evaluation of isopolar, iso-, and nonisosteric phosphonate analogues of natural nucleotides and oligonucleotides. Building on our established chemistry of protected cytosine nucleoside C3′-phosphonates as monomers for oligonucleotide synthesis, we prepared a series of structurally diverse **ODN 2006** analogues modified at all four CpG motifs to enable a more detailed structure–activity relationship (SAR) analysis. Whereas base and sugar modifications within CpG motifs and their effects on TLR9 activation have been extensively investigated, isopolar phosphonate substitutions of internucleotide phosphodiester linkages in CpG oligonucleotides have not, to our knowledge, been systematically synthesized and evaluated. These linkages provide an alternative to conventional phosphorothioate backbones.

Incorporation of selected phosphonate nucleotide analogues (e.g., **I**, **II**, **VII**, and **IX**) increased the potency of **ODN 2006**, identifying isopolar phosphonate modification as an effective strategy for modulating its activity. Notably, oligonucleotide **ODN1** exhibited a 30-fold increase in potency relative to **ODN 2006**, suggesting that conformationally constrained internucleotide linkages within CpG motifs promote a preorganized sugar–phosphonate backbone conformation favorable for TLR9 recognition. In addition, the hexamer **ODN-B** containing modification **I** acts as a ligand for the secondary binding site and, in combination with **ODN 2006**, enhances hTLR9 activation.

Overall, this SAR study of **ODN 2006** analogues highlights conformationally constrained internucleotide linkages as a promising strategy for the development of potent human TLR9 ligands and provides new insights into how the backbone architecture influences CpG DNA recognition. Our findings suggest that backbone engineering may provide a useful strategy for the design of new immunostimulatory oligonucleotides.

## Supplementary Material





## Notes

SMILES strings for compounds **1**–**16** (CSV)
Synthetic procedures for monomer synthesis, characterization data, HPLC chromatograms of prepared monomers and oligonucleotides, and MALDI-TOF MS spectra of prepared oligonucleotides (PDF)

## Abbreviations

1-MeIm: 1-methylimidazole
Ac_2_O: acetic anhydride
ACN: acetonitrile
cDC: conventional dendritic cell
DCA: dichloroacetic acid
DCM: dichloromethane
DDTT: 3-((dimethylamino)­methylidene)­amino)-3*H*-1,2,4-dithiazole-5-thione (Reagent *II*)
DMF: dimethylformamide
DNA: DNA
ETT: 5-ethylthio-1*H*-tetrazole
HPLC: high-performance liquid chromatography
hTLR9: human Toll-like receptor 9
IE-HPLC: ion-exchange high-performance liquid chromatography
IFN-α: interferon alpha
IFN-γ: interferon gamma
IL-6: interleukin-6
LCAA-CPG: long-chain alkylamine controlled pore glass
mTLR9: murine Toll-like receptor 9
Mop: 4-hydroxy-1-oxido-2-pyridylmethyl
NEP-Cl: 2-chloro-5,5-dimethyl-1,3,2-dioxaphosphorinane-2-oxide
NF-κB: nuclear factor kappa B
NK: natural killer
ODN: oligodeoxynucleotide
PBMC: peripheral blood mononuclear cell
PS: phosphorothioate
RNA: ribonucleic acid
SEAP: secreted embryonic alkaline phosphatase
THF: tetrahydrofuran
TLR9: Toll-like receptor 9
