# Supplementary material for: Conformationally Constrained Phosphonate Backbone Linkages Enhance CpG Oligonucleotide Activation of Toll-Like Receptor 9
Source: ACS Pharmacol Transl Sci. 2026 Jul 29;9(8):2324–32. doi: 10.1021/acsptsci.6c00326 (PMC13482898; doi:10.1021/acsptsci.6c00326)
Supplement: Supplementary file 1 [file pt6c00326_si_002.pdf]

Conformationally Constrained Phosphonate Backbone Linkages Enhance CpG Oligonucleotide Activation of Toll-Like Receptor 9

Ivana Kóšiová[*,†], Ivan Štěpánek[†], Magdalena Petrová, Ondřej Páv, Ondřej Šimák, Radek Liboska, Ondřej Kostov, Dominik Rejman, Miloš Buděšínský, Šárka Rosenbergová, Gabriel Birkuš[*], and Ivan Rosenberg

Institute of Organic Chemistry and Biochemistry, Czech Academy of Sciences v.v.i., Flemingovo nám. 2, 166 10 Prague 6, Czech Republic

[†] Authors Ivana Kóšiová and Ivan Štěpánek contributed equally

[*] Corresponding authors: ivana.kosiova@uochb.cas.cz, gabriel.birkus@uochb.cas.cz

Table of Contents:

Synthesis of monomers 2
Experimental 4
  General information 4
  Experimental procedures for monomer synthesis 4
Additional data 10
  NMR data of prepared monomers 10
  Purity data of prepared monomers 15
  LC-MS chromatograms of prepared monomers 16
  MALDI-TOF MS and purity data for prepared ODNs 24
  Purity chromatograms and MALDI-TOF MS of prepared oligonucleotides 25
ODN stability in human serum 44
Abbreviations 45
References: 46

Synthesis of monomers

Monomers **1-4** (Scheme S1) were prepared according to previously reported procedures,[1,2] starting from corresponding diethyl esters **18** (prepared from protected nucleoside **17** as mixture of epimers, with *S*-epimer as major product), **19** and **20** (both intermediates prepared from protected nucleoside **17** and formed exclusively as the *S*-epimer). Phosphonate diethylesters **22-24** (epimers separated after cleavage of 5´-*O*-TBDPS group and introducing DMTr protecting group) were converted to 4-hydroxy-1-oxido-2-pyridylmethyl (MOP) phosphono esters **1-4** using our standard three-step procedure: *1.* deprotection of diethylester groups using TMSBr in the presence of 2,6-lutidine; *2.* complete esterification of free phosphonic acid with 2-(hydroxymethyl)-4-methoxypyridine *N*-oxide (MOP-OH) in the presence of 2-chloro-5,5-dimethyl-1,3,2-dioxaphosphorinane 2-oxide (NEP-Cl) to obtain bisMOP derivative; and *3.* deprotection of one MOP ester group with thiophenol.

The synthesis of monomers **5** and **6** (Scheme S1) started from phosphonate diethylesters **26** and **27**, prepared from protected nucleoside **17** *via* common intermediate **25** according to previously reported method.[3] Application of the same standard three-step protocol afforded monomers **5** and **6** as a mixture of diastereomers.

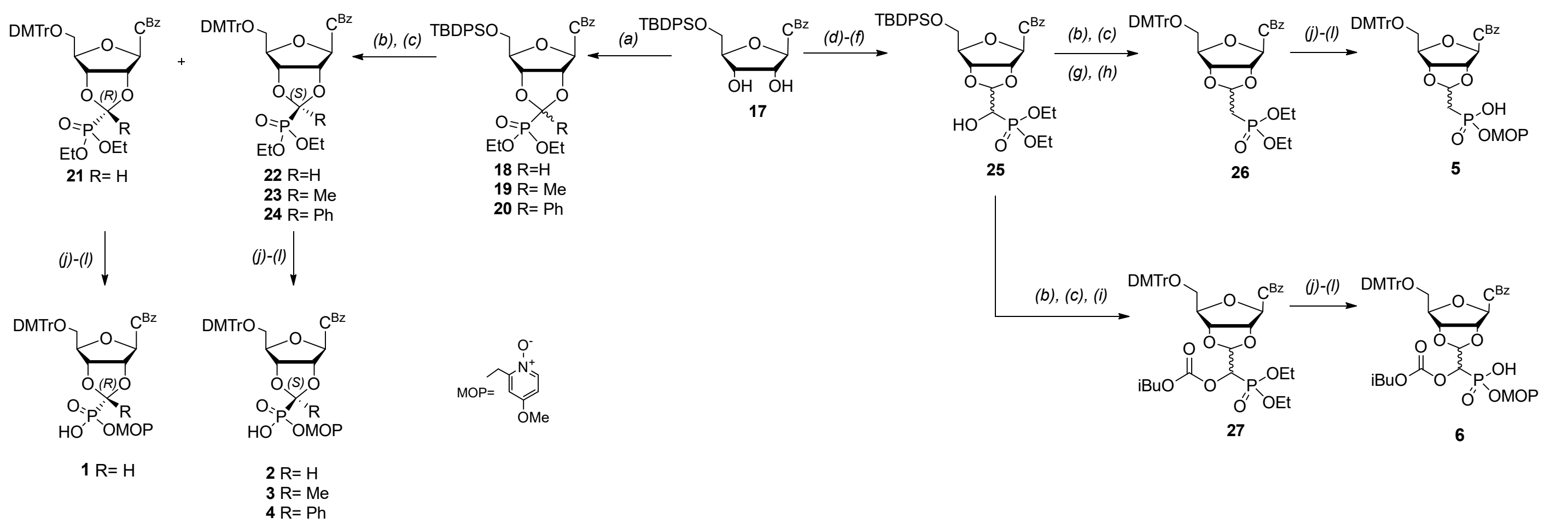

Scheme S1. Synthesis of monomers **1-6** *(a)* 1. trialkylortho ester, pTsOH, DCM, 25 °C, 2. diethyl chlorophosphite, ACN, 25 °C; *(b)* TBAF, THF, 25 °C; *(c)* DMTrCl, pyridine, 25 °C; *(d)* acrolein dimethylacetal, TsOH, DCM, 25 °C; *(e)* 1. OsO_4, THF/H_2O (1/1), 25 °C, 2. $NaIO_4$, acetone/H_2O (1/1), 25 °C; *(f)* diethylphosphite, Et_3N, DCM; *(g)* 1,1'-thiocarbonyldiimidazole, 1,2-dichloroethane, 25 °C; *(h)* AIBN, Bu_3SnH, toluene, reflux; *(i)* isobutyl 1-*H*-tetrazole-1-carboxylate, DMAP, THF, 25 °C; *(j)* TMSBr, 2,6-lutidine, ACN, 25 °C; *(k)* MOP-OH, NEP-Cl, pyridine, 25 °C; *(l)* PhSH, Et_3N, dioxane, 25 °C

The synthesis of 1,4-dioxane phosphonates **7**-**10** (Scheme S2) started from hemiacetals **28** and **29**, prepared according to previously reported procedures.[2] Direct phosphonylation with diethyl trimethylsilyl phosphite afforded 5´-*O*-TBDPS protected phosphonates. After cleavage of 5´-*O*-TBDPS group and introducing DMTr protecting group, the epimeric pairs **30**/**31** and **32**/**33** were readily separated by silica gel chromatography. Finally, phosphonate diethylesters were converted to MOP phosphono esters **7**-**10** using the same standard three-step procedure.

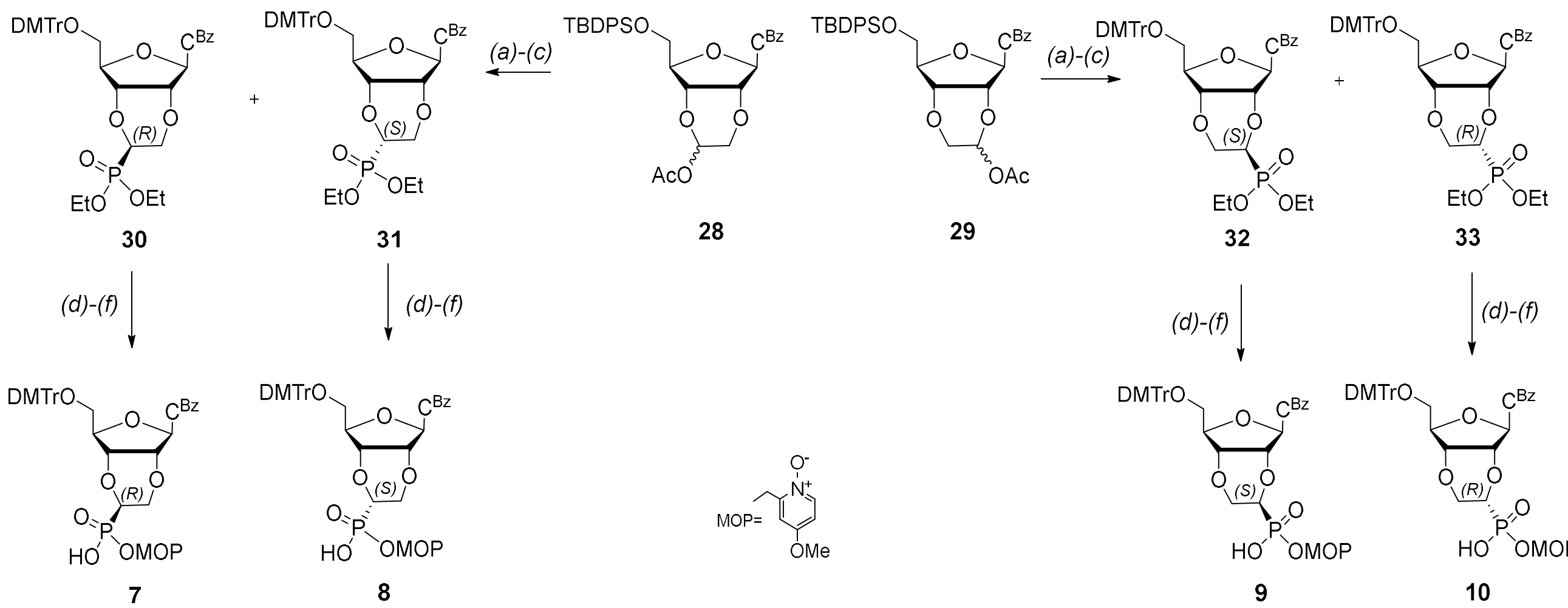

Scheme S2. Synthesis of monomers **7-10** *(a)* diethyl trimethylsilyl phosphite, TMSOTf, ACN, 25 °C *(b)* TBAF, THF, 25 °C; *(c)* DMTrCl, pyridine, 25 °C; *(d)* TMSBr, 2,6-lutidine, ACN, 25 °C; *(e)* MOP-OH, NEP-Cl, pyridine, 25 °C*; (f)* PhSH, Et_3N, dioxane, 25 °C

The synthesis of monomers with 2' or 3'-*O*-methylphosphonate units (**11-14**) started from corresponding protected nucleosides **34**-**37**[4–6] (Scheme S3). Reaction with monomethyl tosyloxymethanephosphonate reagent[7] afforded monomethyl methylphosphonate intermediates, which were subsequently treated with MOP-OH in the presence of NEP-Cl. The resulting mixed diesters were treated with 60% aqueous pyridine to cleave the remaining methyl ester group, yielding the final MOP phosphono esters **11**-**14**.

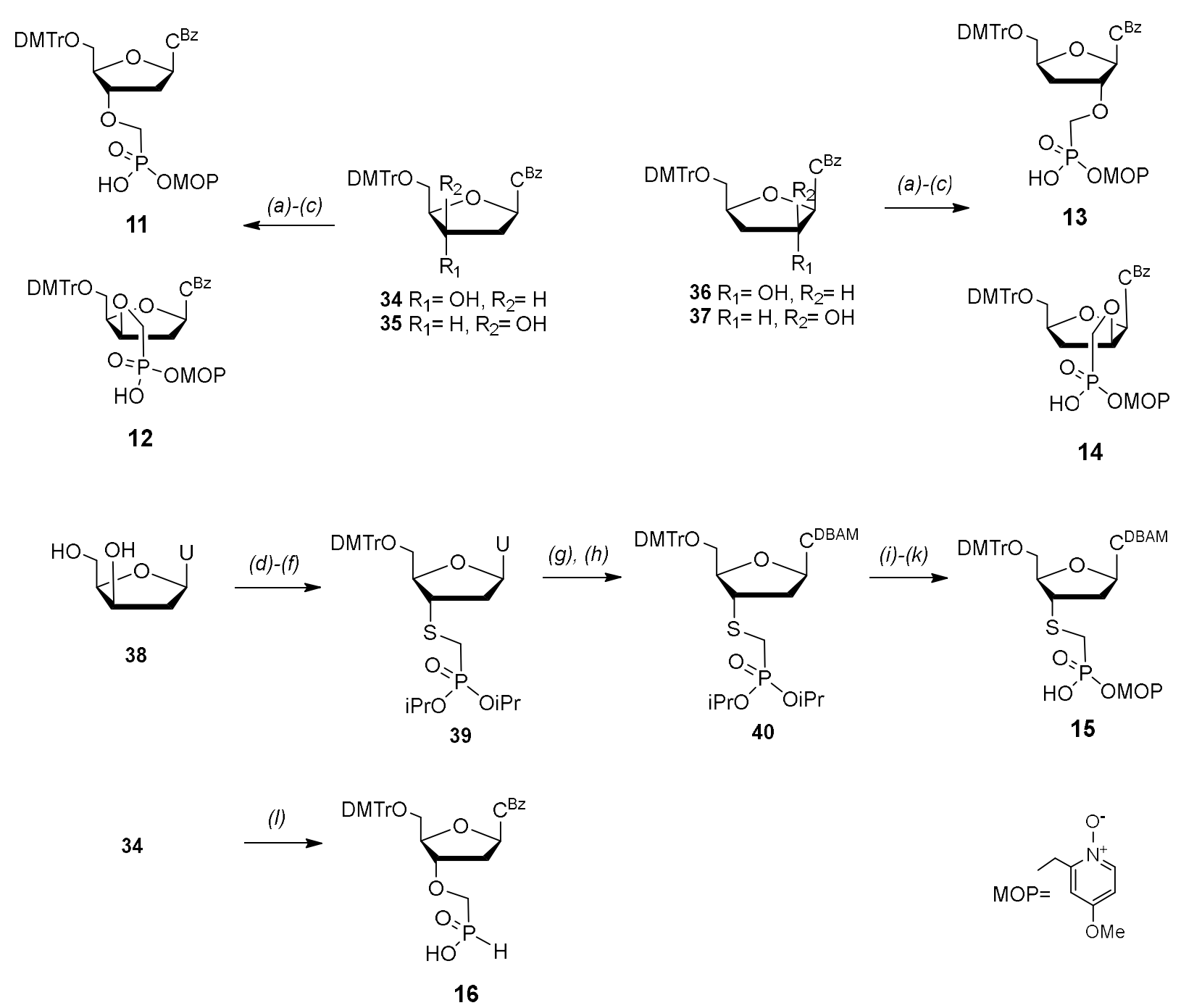

Scheme S3. Synthesis of monomers **11-16** *(a)* NaH, $TsOCH_2P(O)(OMe)(OH)$, DMF, -30 °C to 25 °C; *(b)* MOP-OH, NEP-Cl, pyridine, 25 °C; *(c)* 60% aqueous pyridine, 60 °C*; (d)* DMTrCl, pyridine, 25 °C; *(e)* MsCl, pyridine, cat. DMAP, DCM, 0 °C; *(f) S*-(diisopropylphosphonomethyl)isothio-uronium tosylate, iPrONa, DMF, 25 °C; *(g)* 1. Et_3N, DMAP, TiPBSCl*,* ACN, 25 °C 2. NH_4OH, 0 °C - 25 °C; *(h) N,N'*-dimethylformamide dibutylactetal, methanol; *(i)* TMSBr, 2,6-lutidine, ACN, 25 °C; *(j)* MOP-OH, NEP-Cl, pyridine, 25 °C; *(k)* PhSH, Et_3N, dioxane, 25 °C; *(l)* 4-toluenesulfonyloxymethyl-(*H*)-phosphinic acid, NaH, DMF, -40 °C to 25 °C

The synthesis of monomer **15** started from protected uridine derivative **38**[8] (Scheme S3). The mesylated intermediate was phosphonylated with *in situ* generated diisopropyl sulfanylmethylphosponate[9] to provide intermediate **39**. Subsequent conversion of uracil nucleobase to cytosine, followed by protection of nucleobase amino group, conversion of diisopropyl ester **40** into MOP ester, using the standard procedure, led to desired product **15**. Monomer **16** was prepared according to previously reported procedure[10] from protected nucleoside **34** (Scheme S3).

Experimental

General information

Unless otherwise stated, all solvents and reagents were purchased from commercial suppliers and used without further purification and according to the manufacturers' recommendations. Mass spectra were recorded on the ZAB-EQ (VG Analytical) instrument using FAB (ionization with Xe, accelerating voltage 8 kV; glycerol and thioglycerol as matrices) and on the LTQ Orbitrap XL (Thermo Fisher Scientific) using ESI ionization. MALDI-TOF MS was performed on Reflex IV (Bruker - Daltonics) instrument using 3-hydroxypyridine-2-carboxylic acid and pyridine-2-carboxylic acid as matrices. The NMR spectra were measured on the Bruker AVANCE-600 instrument (^{1}H at 600.13 MHz and ^{13}C at 150.9 MHz) with a cryoprobe and the Bruker AVANCE-500 instrument with a cryoprobe (^{31}P at 202.4 MHz) in d_6-DMSO at 25°C. The ^{1}H and ^{13}C spectra were referenced to the solvent peak (using δ_H(DMSO) = 2.50 ppm; δ_C(DMSO) = 39.7 ppm) and ^{31}P spectra to external H_3PO_4. The structural assignment of the proton and carbon signals was achieved combining 1D-1H and 13C-spectra with homonuclear 2D-H,H-COSY, 2D-H,H-ROESY and heteronuclear 2D-H,C-HSQC and 2D-H,C-HMBC spectra. NMR assigments of prepared monomers are listed in Tables S1-S3. Purity of prepared monomers was determined by LC-MS performed on Waters Alliance HPLC System with MS SQ Detector 2 (Waters, Milford, USA) using LUNA C18 column (Phenomenex, 100 × 4.6 mm, 3 µm) and 10 mM ammonium acetate buffer pH 6.8 with linear gradient of acetonitrile (0 to 100% in 20 min; or 2.5% to 100% in 15 min, flow rate 1 mL/min, column temperature 40°C, injection volume 5µL). The analytes were detected by PDA detector (254 nm channel), and MS detector (ESI scan). Original software (MassLynx) was used for instrument control and data acquisition. MestReNova software was used for data analysis. Oligonucleotides were synthesized on an automated GenSyn DNA/RNA synthesizer (IOCB Prague). After deprotection and cleavage from the solid support, crude oligonucleotides were analyzed by ion-exchange HPLC on a Waters HPLC system equipped with a DNAPac PA100 column (4 × 250 mm; Dionex) using appropriate gradients at a flow rate of 1.0 mL/min. Detection was performed with a PDA detector at 254 nm. Preparative purification was carried out on a DNAPac PA100 column (10 × 250 mm; Dionex) at a flow rate of 3.0 mL/min. Purified oligonucleotides were desalted on a Luna C18 column (4 × 30 mm, 10 µm; Phenomenex) using a linear gradient of methanol (0–90% over 20 min) in 0.05 M aqueous triethylammonium bicarbonate (TEAB) buffer at a flow rate of 2.0 mL/min.

Experimental procedures for monomer synthesis

2-(((((2S,3aR,4R,6R,6aR)-4-(4-benzamido-2-oxopyrimidin-1(2H)-yl)-6-((bis(4-methoxyphenyl)(phenyl)methoxy)methyl)tetrahydrofuro[3,4-d][1,3]dioxol-2-yl)(hydroxy)phosphoryl)oxy)methyl)-4-methoxypyridine 1-oxide (1) Title compound was prepared from **17** according to previously reported methods.[1,2] All spectroscopic data were in agreement with the literature.

2-(((((2R,3aR,4R,6R,6aR)-4-(4-benzamido-2-oxopyrimidin-1(2H)-yl)-6-((bis(4-methoxyphenyl)(phenyl)methoxy)methyl)tetrahydrofuro[3,4-d][1,3]dioxol-2-yl)(hydroxy)phosphoryl)oxy)methyl)-4-methoxypyridine 1-oxide (2) Title compound was prepared from **17** according to previously reported methods.[1,2] All spectroscopic data were in agreement with the literature.

2-(((((2S,3aR,4R,6R,6aR)-4-(4-benzamido-2-oxopyrimidin-1(2H)-yl)-6-((bis(4-methoxyphenyl)(phenyl)methoxy)methyl)-2-methyltetrahydrofuro[3,4-d][1,3]dioxol-2-yl)(hydroxy)phosphoryl)oxy)methyl)-4-methoxypyridine 1-oxide (3) Title compound was prepared from **18** (500 mg, 0.669 mmol) in 20% overall yield (120 mg, 0.134 mmol) according to previously reported methods.[1,2] HRMS $(M+H)^+$ for $C_{46}H_{46}O_{13}N_4P$ calculated: 893.27935 found: 893.27926.

2-(((((2S,3aR,4R,6R,6aR)-4-(4-benzamido-2-oxopyrimidin-1(2H)-yl)-6-((bis(4-methoxyphenyl)(phenyl)methoxy)methyl)-2-phenyltetrahydrofuro[3,4-d][1,3]dioxol-2-yl)(hydroxy)phosphoryl)oxy)methyl)-4-methoxypyridine 1-oxide (4) Title compound was prepared from **19** (500 mg, 0.615 mmol) in 38% overall yield (223 mg, 0.233 mmol) according to previously reported methods.[1,2] HRMS $(M+H)^+$ for $C_{51}H_{48}O_{13}N_4P$ calculated: 955.29500 found: 955.29490.

2-((((((3aR,4R,6R,6aR)-4-(4-benzamido-2-oxopyrimidin-1(2H)-yl)-6-((bis(4-methoxyphenyl)(phenyl)methoxy)methyl)tetrahydrofuro[3,4-d][1,3]dioxol-2-yl)methyl)(hydroxy)phosphoryl)oxy)methyl)-4-methoxypyridine 1-oxide (5) A solution of **26** (350 mg, 0.431 mmol) in ACN (5 mL) was treated at 25 °C with TMSBr (0.23 mL, 1.724 mmol) and 2,6-lutidine (0.40 mL, 3.449 mmol). After 14 h, reaction mixture was concentrated *in vacuo*, the residue was dissolved in chloroform (50 mL) and washed with 10% aqueous solution of citric acid (50 mL) and 0.2M TEAB (2x 50 mL). The organic phase was dried over anhydrous Na_2SO_4, filtered, concentrated *in vacuo* and the residue co-evaporated with pyridine (2x 10 mL). Crude phosphonic acid was used in the next step without further purification. solution of phosphonic acid (0.431 mmol) in pyridine (5 mL) was treated at room temperature with 2-(hydroxymethyl)-5-methoxypyridine *N*-oxide (200 mg, 1.293 mmol) and 2-chloro-5,5-dimethyl-1,3,2-dioxaphosphorinane 2-oxide (238 mg, 1.293 mmol). After 2 h, 2M TEAB (2 mL) was added, and reaction mixture was concentrated *in vacuo*. The residue was dissolved in chloroform (50 mL) and washed with 0.2 M TEAB (2x50 mL). The organic phase was dried over anhydrous Na_2SO_4, filtered, and concentrated *in vacuo*. Crude MOP diester was used in the next step without further purification.

A solution of diester (0.431 mmol) in dioxane (5 mL) was treated with thiophenol (430 µL, 4.187 mmol) and triethylamine (640 µL, 4.591 mmol). After 1 h, reaction mixture was poured directly on silica gel column (silica gel treated with 1% Et_3N) and product was purified by chromatography with linear gradient of 0-100% mixture of ethyl acetate/ethanol/acetone/water 4:1:1:1 in ethyl acetate to give **5** (260 mg, 0.291 mmol) in 67% overall yield. HRMS $(M+Na)^+$ for $C_{46}H_{45}O_{13}N_4NaP$ calculated: 915.26129 found: 915.26090.

2-((((((3aR,4R,6R,6aR)-4-(4-benzamido-2-oxopyrimidin-1(2H)-yl)-6-((bis(4-methoxyphenyl)(phenyl)methoxy)methyl)tetrahydrofuro[3,4-d][1,3]dioxol-2-yl)((isobutoxycarbonyl)oxy)methyl)(hydroxy)phosphoryl)oxy)methyl)-4-methoxypyridine 1-oxide (6) Title compound was prepared from **27** (650 mg, 0.745 mmol) in 74% overall yield (560 mg, 0.555 mmol) according to method used for **5**. HRMS $(M+Na)^+$ for $C_{51}H_{53}O_{16}N_4NaP$ calculated: 1031.30864 found: 1031.90886.

2-(((((2R,4aR,5R,7R,7aR)-5-(4-benzamido-2-oxopyrimidin-1(2H)-yl)-7-((bis(4-methoxyphenyl)(phenyl)methoxy)methyl)hexahydrofuro[3,4-b][1,4]dioxin-2-yl)(hydroxy)phosphoryl)oxy)methyl)-4-methoxypyridine 1-oxide (7) Title compounds was prepared from **28**

according to previously reported procedure.[2] HRMS $(M+Na)^+$ for $C_{46}H_{45}O_{13}N_4NaP$ calculated: 915.26129 found: 915.26091.

2-(((((2S,4aR,5R,7R,7aR)-5-(4-benzamido-2-oxopyrimidin-1(2H)-yl)-7-((bis(4-methoxyphenyl)(phenyl)methoxy)methyl)hexahydrofuro[3,4-b][1,4]dioxin-2-yl)(hydroxy)phosphoryl)oxy)methyl)-4-methoxypyridine 1-oxide (8) Title compounds was prepared from **28** according to previously reported procedure.[2] HRMS $(M+Na)^+$ for $C_{46}H_{45}O_{13}N_4NaP$ calculated: 915.26129 found: 915.26096.

2-(((((2S,4aR,5R,7R,7aR)-7-(4-benzamido-2-oxopyrimidin-1(2H)-yl)-5-((bis(4-methoxyphenyl)(phenyl)methoxy)methyl)hexahydrofuro[3,4-b][1,4]dioxin-2-yl)(hydroxy)phosphoryl)oxy)methyl)-4-methoxypyridine 1-oxide (9) Title compound was prepared from **29** according to previously reported procedure.[2] HRMS $(M-H)^-$ for $C_{46}H_{44}O_{13}N_4P$ calculated: 891.26480 found: 891.26346.

2-(((((2R,4aR,5R,7R,7aR)-7-(4-benzamido-2-oxopyrimidin-1(2H)-yl)-5-((bis(4-methoxyphenyl)(phenyl)methoxy)methyl)hexahydrofuro[3,4-b][1,4]dioxin-2-yl)(hydroxy)phosphoryl)oxy)methyl)-4-methoxypyridine 1-oxide (10) Title compound was prepared from **29** according to previously reported procedure.[2] HRMS $(M+Na)^+$ for $C_{46}H_{45}O_{13}N_4NaP$ calculated: 915.26129 found: 915.26168.

2-((((((2R,3S,5R)-5-(4-benzamido-2-oxopyrimidin-1(2H)-yl)-2-((bis(4-methoxyphenyl)(phenyl)methoxy)methyl)tetrahydrofuran-3-yl)oxy)methyl)(hydroxy)phosphoryl)oxy)methyl)-4-methoxypyridine 1-oxide (11) A solution of **34** (1.0 g, 1.58 mmol) in DMF (20 mL) was cooled down to -30°C and treated with NaH (0.253 g, 6.32 mmol, 60% suspension in mineral oil). After 30 minutes at -30 °C, monomethyl tosyloxymethanephosphonate (0.885 g, 3.16 mmol) was added to the reaction mixture, and stirred until starting material disappeared (reaction mixture was gradually left to warm up to room temperature). Then the reaction mixture was cooled down to -30 °C, carefully neutralized with glacial acetic acid and concentrated *in vacuo*. The residue was dissolved in chloroform (100 mL) and washed with 1M TEAB (3x50 mL). The organic phase was dried over anhydrous Na_2SO_4, filtered, concentrated *in vacuo* and the methyl ester intermediate was purified by chromatography on silica gel (linear gradient of 0-100% mixture of ethyl acetate/ethanol/acetone/water 4:1:1:1 in ethyl acetate, silica gel treated with 1% Et_3N). A solution of the intermediate (1.0 g, 1.35 mmol) in pyridine (20 mL) was treated at 25 °C with MOP-OH (0.628 g, 4.05 mmol) and NEP-Cl (0.747 g, 4.05 mmol) until starting material disappeared. Then 5 mL of 2M TEAB was added, and reaction mixture was concentrated *in vacuo*, dissolved in chloroform (100 mL) and washed with 0.2 M TEAB (3x 50 mL). The organic phase was dried over anhydrous Na_2SO_4, filtered, and concentrated *in vacuo*. The residue was treated with 60% aqueous pyridine (40 mL) at 60 °C. After 16 h, the reaction mixture was concentrated *in vacuo*, the residue was dissolved in chloroform (100 mL) and washed with 0.2 M TEAB (3x 50 mL). The organic phase was dried over anhydrous Na_2SO_4, filtered, and concentrated *in vacuo*. Product was purified by chromatography on silica gel (linear gradient of 0-100% mixture of ethyl acetate/ethanol/acetone/water 4:1:1:1 in ethyl acetate, silica gel treated with 1% Et_3N). Product **11** was prepared in 55% overall yield (640 mg, 0.74 mmol). Obtained spectroscopic data were in agreement with literature.[2]

2-((((((2R,3R,5R)-5-(4-benzamido-2-oxopyrimidin-1(2H)-yl)-2-((bis(4-methoxyphenyl)(phenyl)methoxy)methyl)tetrahydrofuran-3-yl)oxy)methyl)(hydroxy)phosphoryl)oxy)methyl)-4-methoxypyridine 1-oxide (12) Title compound was

prepared from **35** (500 mg, 0.789 mmol) using method described above (**11**) in 50% overall yield (250 mg, 0.29 mmol). HRMS $(M-H)^-$ for $C_{45}H_{44}O_{12}N_4P$ calculated: 863.26988 found: 863.26862.

2-((((((2R,3R,5S)-2-(4-benzamido-2-oxopyrimidin-1(2H)-yl)-5-((bis(4-methoxyphenyl)(phenyl)methoxy)methyl)tetrahydrofuran-3-yl)oxy)methyl)(hydroxy)phosphoryl)oxy)methyl)-4-methoxypyridine 1-oxide (13) Title compound was prepared from **36** (750mg, 1.18 mmol) using method described above (**11**) in 41% overall yield (426 mg, 0.486 mmol). HRMS $(M-H)^-$ for $C_{45}H_{44}O_{12}N_4P$ calculated: 863.26988 found: 863.26945.

2-((((((2R,3S,5S)-2-(4-benzamido-2-oxopyrimidin-1(2H)-yl)-5-((bis(4-methoxyphenyl)(phenyl)methoxy)methyl)tetrahydrofuran-3-yl)oxy)methyl)(hydroxy)phosphoryl)oxy)methyl)-4-methoxypyridine 1-oxide (14) Title compound was prepared from **37** (800 mg, 1.26 mmol) using method described above (**11**) in 60% overall yield (660 mg, 0.76 mmol). HRMS $(M-H)^-$ for $C_{45}H_{44}O_{12}N_4P$ calculated: 863.26988 found: 863.26888.

2-((((((2R,3S,5R)-2-((bis(4-methoxyphenyl)(phenyl)methoxy)methyl)-5-(4-(((Z)-(dibutylamino)methylene)amino)-2-oxopyrimidin-1(2H)-yl)tetrahydrofuran-3-yl)thio)methyl)(hydroxy)phosphoryl)oxy)methyl)-4-methoxypyridine 1-oxide (15) A solution of **40** (1.0 g, 1.15 mmol) in ACN (10 mL) was treated at 25 °C with TMSBr (0. 60 mL, 4.6 mmol) and 2,6-lutidine (1.1 mL, 9.2 mmol). After 14 h, reaction mixture was concentrated *in vacuo*, the residue was dissolved in chloroform (50 mL) and washed with 10% aqueous solution of citric acid (50 mL) and 0.2M TEAB (2x 50 mL). The organic phase was dried over anhydrous Na_2SO_4, filtered, concentrated *in vacuo* and the residue co-evaporated with pyridine (2x 10 mL). Crude phosphonic acid was used in the next step without further purification. A solution of phosphonic acid (1.15 mmol) in pyridine (10 mL) was treated at at 25 °C with 2-(hydroxymethyl)-5-methoxypyridine *N*-oxide (540 mg, 3.48 mmol) and 2-chloro-5,5-dimethyl-1,3,2-dioxaphosphorinane 2-oxide (642 mg, 3.48 mmol). After 2 h, 2M TEAB (3 mL) was added, and reaction mixture was concentrated *in vacuo*. The residue was dissolved in chloroform (50 mL) and washed with 0.2 M TEAB (2x50 mL). The organic phase was dried over anhydrous Na_2SO_4, filtered, and concentrated *in vacuo*. Crude MOP diester was used in the next step without further purification. A solution of crude MOP diester (1.15 mmol) in dioxane (3 mL) was treated at 25 °C with thiophenol (1 mL) and Et_3N (1.3 mL). After 1 h, reaction mixture was poured directly on silica gel column (silica gel treated with Et_3N) and product was purified by chromatography with linear gradient of 0-100% mixture of ethyl acetate/ethanol/acetone/water 4:1:1:1 in ethyl acetate to give **15** (460 mg, 0.5 mmol) in 44% overall yield. HRMS $(M+H)^+$ for $C_{47}H_{59}O_{10}N_5PS$ calculated: 916.37148 found: 916.37082.

((((2R,3S,5R)-5-(4-benzamido-2-oxopyrimidin-1(2H)-yl)-2-((bis(4-methoxyphenyl)(phenyl)methoxy)methyl)tetrahydrofuran-3-yl)oxy)methyl)phosphinic acid (16) Title compound was prepared from **34** according to previously reported method.[10] All spectroscopic data were in agreement with the literature.

Diethyl (((3aR,4R,6R,6aR)-4-(4-benzamido-2-oxopyrimidin-1(2H)-yl)-6-(((tert-butyldiphenylsilyl)oxy)methyl)tetrahydrofuro[3,4-d][1,3]dioxol-2-yl)(hydroxy)methyl)phosphonate (25) Title compound was prepared from **17** in 75% yield (1.8 g, 2.356 mmol) according to previously reported method.[3] HRMS $(M+H)^+$ for $C_{38}H_{47}O_{10}N_3PSi$ calculated: 764.27628 found: 764.27641.

Diethyl (((3aR,4R,6R,6aR)-4-(4-benzamido-2-oxopyrimidin-1(2H)-yl)-6-((bis(4-methoxyphenyl)(phenyl)methoxy)methyl)tetrahydrofuro[3,4-d][1,3]dioxol-2-yl)methyl)phosphonate (26) A solution of **25** (1.0 g, 1.309 mmol) was treated at 25 °C with TBAF.3H_2O (496 mg, 1.570 mmol). After 2 h, reaction

mixture was diluted in methanol, and DOWEX 50 in Et_3N^+ cycle was added to the stirred solution to remove tetrabutylammonium ions. After 30 minutes, reaction mixture was filtered, concentrated *in vacuo*, and residue was purified by chromatography on silica gel (linear gradient of 0-10% mixture of ethanol in chloroform) to give desilylated intermediate (630 mg, 1.199 mmol). A solution of intermediate (630 mg, 1.199 mmol) in pyridine (10 mL) was treated at 25 °C with DMTrCl (487 mg, 1.439 mmol). After 16 h, methanol (2 mL) was added, and reaction mixture was concentrated *in vacuo*. The residue was dissolved in chloroform (50 mL) and washed with 10% aqueous solution of citric acid (50 mL) and saturated aqueous $NaHCO_3$ solution (2x 25 mL). The organic phase was dried over anhydrous Na_2SO_4, filtered, concentrated *in vacuo* and the residue was purified by chromatography on silica gel (linear gradient of 0-10% mixture of ethanol in chloroform) to give 5'-protected intermediate (800 mg, 0.966 mmol). A solution of intermediate (800 mg, 0.966 mmol) in 1,2-dichloroethane (10 mL) was treated with 1,1'-thiocarbonyl imidazole (344 mg, 1.932 mmol) at 25 °C. After 16 h, the reaction mixture was diluted with chloroform (10 mL) and imidazoylthiocarbonyl intermediate was purified by chromatography on silica gel (linear gradient of 0-5% ethanol in chloroform). Stirred solution of imidazoylthiocarbonyl derivative (0.889 mmol) was treated with tributyltinhydride (470 µL, 1.777 mmol) and catalytic amount of AIBN (10 mg, 0.06 mmol) at 120 °C. After 1 h, reaction mixture was cooled down, poured directly onto a silica gel column and product was purified by chromatography (linear gradient of 0-50% acetone in toluene) to give **26** in 52% overall yield (552 mg, 0.679 mmol). HRMS $(M+H)^+$ for $C_{43}H_{47}O_{11}N_3P$ calculated: 812.29427 found: 812.29442.

((3aR,4R,6R,6aR)-4-(4-benzamido-2-oxopyrimidin-1(2H)-yl)-6-((bis(4-methoxyphenyl)(phenyl)methoxy)methyl)tetrahydrofuro[3,4-d][1,3]dioxol-2-yl)(diethoxyphosphoryl)methyl isobutyl carbonate (27) A solution of **25** (800 mg, 1.047 mmol) was treated at 25 °C with TBAF.3H_2O (396 mg, 1.257 mmol). After 2 h, reaction mixture was diluted in methanol, and DOWEX 50 in Et_3N^+ cycle was added to the stirred solution to remove tetrabutylammonium ions. After 30 minutes, reaction mixture was filtered, concentrated *in vacuo*, and residue was purified by chromatography on silica gel (linear gradient of 0-10% mixture of ethanol in chloroform) to give desilylated intermediate (450 mg, 0.856 mmol). A solution of intermediate (450 mg, 0.856 mmol) in pyridine (10 mL) was treated at 25 °C with DMTrCl (348 mg, 1.027 mmol). After 16 h, methanol (2 mL) was added, and reaction mixture was concentrated *in vacuo*. The residue was dissolved in chloroform (50 mL) and washed with 10% aqueous solution of citric acid (50 mL) and saturated aqueous $NaHCO_3$ solution (2x 25 mL). The organic phase was dried over anhydrous Na_2SO_4, filtered, concentrated *in vacuo* and the residue was purified by chromatography on silica gel (linear gradient of 0-10% mixture of ethanol in chloroform) to give 5'-protected intermediate (650 mg, 0.785 mmol). A solution of intermediate (650 mg, 0.785 mmol) in THF (7 mL) was treated at room temperature with isobutyl 1-*H*-tetrazole-1-carboxylate (160 mg, 0.942 mmol) and 4-dimethylaminopyridine (115 mg; 0.942 mmol). After 2 h, methanol (1 mL) was added, and the reaction mixture was concentrated *in vacuo*. The residue was dissolved in chloroform and washed with 10% aqueous solution of citric acid (50 mL) and 0.2M TEAB (2x 50 mL). The organic phase was dried over anhydrous Na_2SO_4, filtered, concentrated *in vacuo* and the residue was purified by chromatography on silica gel (linear gradient of 0-10% ethanol in chloroform) to give **27** (660 mg, 0.711 mmol) in 68% overall yield. HRMS $(M+Na)^+$ for $C_{48}H_{54}O_{14}N_3NaP$ calculated: 950.32356 found: 950.32345.

Diisopropyl ((((2R,3S,5R)-2-((bis(4-methoxyphenyl)(phenyl)methoxy)methyl)-5-(2,4-dioxo-3,4-dihydropyrimidin-1(2H)-yl)tetrahydrofuran-3-yl)thio)methyl)phosphonate (39) A solution of **38** (1.25 g, 5.5 mmol) in pyridine (50 mL) was treated with DMTrCl (2.2 g, 6.6 mmol) at 25 °C. After 16 h, reaction mixture was quenched with methanol (3 mL), reaction mixture was concentrated *in vacuo*, diluted with chloroform (200 mL) and washed with saturated solution of $NaHCO_3$ (2x 100 mL), 10 % aqueous solution of citric acid (1x 100 mL) and 0.1 M TEAB (100 ml). The organic phase was dried over anhydrous Na_2SO_4, filtered, and concentrated *in vacuo*. Product was purified by chromatography on silica gel (linear gradient of 0-10% ethanol in chloroform) to give 5'-*O*-DMTr intermediate (2.4 g, 4.52 mmol).

A solution of 5'-*O*-DMTr intermediate (2.4 g, 4.52 mmol) in DCM (45 mL) was treated with pyridine (3.6 mL, 45.2 mmol), catalytical amount of DMAP (55 mg, 0.45 mmol) and cooled down to 0°C. Mesyl chloride (1.8 mL, 22.6 mmol) was added dropwise under stirring and reaction mixture was left to warm-up to room temperature. After 5 h, reaction mixture was cooled down to 0°C, reaction was quenched with saturated solution of $NaHCO_3$ (10 mL), mixture was diluted with DCM (200 mL), washed with saturated solution of $NaHCO_3$ (2x 100 mL) and water (1x100 mL). The organic phase was dried over anhydrous Na_2SO_4, filtered, and concentrated *in vacuo*. Product was purified by chromatography on silica gel (linear gradient of 0-10% ethanol in chloroform) to give mesyl intermediate (2.0 g, 3.3 mmol, 74%).

NaH (792 mg, 19.8 mmol) was added to the mixture of iPrOH (1.5 mL, 19.8 mmol) in DMF (20 mL) and stirred under argon for 15 minutes. *S*-(diisopropylphosphonomethyl)isothio-uronium tosylate[6] (4.2 g, 9.9 mmol) was added to pre-mixed solution of iPrONa in DMF and resulting mixture was stirred at 25 °C for another 30 minutes. Then the mesyl intermediate (2.0 g, 3.3 mmol) in DMF (10 mL) was added, mixture was stirred at 25 °C and after 14 h, cooled down to 0 °C, carefully neutralized with acetic acid and evaporated *in vacuo*. Residue was dissolved in chloroform (200 mL) and washed with 0.2 M TEAB (2 x 100 mL). The organic phase was dried over anhydrous Na_2SO_4, filtered, and concentrated *in vacuo*. Product was purified by chromatography on silica gel (linear gradient of 0-50% acetone in toluene) to give **39** (1.9 g, 2.62 mmol) in 79% yield. HRMS $(M+Na)^+$ for $C_{37}H_{45}O_9N_2NaPS$ calculated: 747.24756 found: 747.24697.

Diisopropyl ((((2R,3S,5R)-2-((bis(4-methoxyphenyl)(phenyl)methoxy)methyl)-5-(4-(((Z)-(dibutylamino)methylene)amino)-2-oxo-3,4-dihydropyrimidin-1(2H)-yl)tetrahydrofuran-3-yl)thio)methyl)phosphonate (40) A solution of **39** (1.9 g, 2.62 mmol) in ACN (25 mL) was treated at room temperature with Et_3N (0.77 mL, 5.52 mmol), DMAP (0.674 g, 5.52 mmol) and TiPBSCl (1.7 g, 5.52 mmol). After 14 h, reaction mixture was cooled down to 0°C and aqueous NH_3 (30 mL) was added to a mixture and reaction mixture was left to warm-up at room temperature. After 2 h, reaction mixture was evaporated *in vacuo*, residue was dissolved in chloroform (100 mL), washed with 10% aqueous solution of citric acid (50 mL) and 0.2 M TEAB (2 x 50 mL). The organic phase was dried over anhydrous Na_2SO_4, filtered, and concentrated *in vacuo*. Product was purified by chromatography on silica gel (linear gradient of 0-20% ethanol in chloroform) to give unprotected intermediate (1.6 g, 2.21 mmol) in 84% yield. A solution of intermediate (1.6 g, 2.2 mmol) in methanol (20 mL) was treated at room temperature with *N,N'*-dimethylformamide dibutylacetal (0.77 mL, 3.3 mmol). After 14 h, reaction mixture was evaporated *in vacuo,* and residue was purified by chromatography on silica gel (linear gradient of 0-10% ethanol in chloroform) to give **40** (1.5 g, 1.74 mmol) in 79% yield. HRMS $(M+H)^+$ for $C_{46}H_{64}O_8N_4PS$ calculated: 863.41770 found: 863.41736.

Additional data

NMR data of prepared monomers

Compound	H-1′	H-2′	H-3′	H-4′	H-5′	H-5″	H-5	H-6
**3**	5.89 d<br>1′,2′ = 1.7	5.19 dd<br>2′,1′ = 1.7<br>2′,3′ ≈ 6.3	4.97 dd<br>3′,2′ = 6.3<br>3′,4′ = 3.8	4.275 dt<br>4′3′ = 3.8<br>4′,5′ = 6.6<br>4′,5″ = 3.4	3.345 dd<br>5′,4′ = 6.6<br>5′,5″ = 10.6	3.12 dd<br>5″,4′ = 3.4<br>5″,5′ = 10.6	7.23 bd<br>5,6 = 7.5	8.18 bd<br>6,5 = 7.5
**4**	5.88 d<br>1′,2′ = 1.3	5.355 dd<br>2′,1′ = 1.3<br>2′,3′ ≈ 6.2	5.14 dd<br>3′,2′ = 6.2<br>3′,4′ = 4.2	4.095 ddd<br>4′3′ = 4.2<br>4′,5′ = 6.5<br>4′,5″= 3.4	3.34 dd<br>5′,4′ = 6.5<br>5′,5″= 10.4	3.13 dd<br>5″,4′ = 3.4<br>5″,5′= 10.4	7.265 bd<br>5,6 = 7.1	8.20 bd<br>6,5 = 7.1
**5**<br>DIAs 50:50	5.90 d<br>1′,2′ = 1.7<br>5.87 d<br>1′,2′ = 2.1	4.92 dd<br>2′,1′ = 1.7<br>2′,3′ ≈ 6.4<br>4.89 dd<br>2′,1′ = 2.1<br>2′,3′ ≈ 6.4	4.69 dd<br>3′,2′ = 6.4<br>3′,4′ = 3.6<br>4.76 dd<br>3′,2′ = 6.4<br>3′,4′ = 4.3	4.31 dt<br>4′3′ = 3.6<br>4′,5′ = 6.4<br>4′,5″ = 3.8<br>4.23 dt<br>4′3′ = 4.3<br>4′,5′ = 6.5<br>4′,5″ = 4.1	3.41 dd<br>5′,4′ = 6.4<br>5′,5″= 10.4<br>3.26 dd<br>5′,4′ = 4.1<br>5′,5″= 10.4	3.37 dd<br>5″,4′ = 6.4<br>5″,5′ = 10.4<br>3.25 dd<br>5″,4′ = 4.1<br>5″,5′ = 10.4	7.26 bd<br>5,6 = 7.5	8.16 bd<br>6,5 = 7.5<br>8.19 bd<br>6,5 = 7.5
**6**	5.85 d<br>1′,2′ = 1.2	5.01 dd<br>2′,1′ = 1.2<br>2′,3′ ≈ 6.2	4.72 dd<br>3′,2′ = 6.2<br>3′,4′ = 4.1	4.275 dt<br>4′3′ = 4.1<br>4′,5′ = 6.9<br>4′,5″ = 3.4	3.30 dd<br>5′,4′ = 6.9<br>5′,5″= 10.2	3.065 dd<br>5″,4′ = 3.4<br>5″,5′ = 10.2	7.29 bd<br>5,6 = 7.2	8.18 bd<br>6,5 = 7.2
**7**	6.57 d<br>1′,2′ = 8.0	4.30 dd<br>2′,1′ = 8.0<br>2′,3′ ≈ 4.3	4.08 d<br>3′,2′ = 4.3<br>3′,4′ ≈ 0	4.04 dd<br>4′3′ ≈ 0<br>4′,5′ = 4.3<br>4′,5″ = 3.3	3.365 dd<br>5′,4′ = 4.3<br>5′,5″= 10.8	3.18 dd<br>5″,4′ = 3.3<br>5″,5′ = 10.8	7.15 bd<br>5,6 = 7.5	8.02 bd<br>6,5 = 7.5
**8**	5.77 s<br>1′,2′ ≈ 0	4.07 d<br>2′,1′ ≈ 0<br>2′,3′≈ 4.3	4.58 dd<br>3′,2′ = 4.3<br>3′,4′ = 9.6	4.69 ddd<br>4′3′ = 9.6<br>4′,5′ = 3.8<br>4′,5″ = 2.0	3.40 dd<br>5′,4′ = 3.8<br>5′,5″= 11.3	3.325 dd<br>5″,4′ = 2.0<br>5″,5′ = 11.3	7.13 bd<br>5,6 = 7.4	8.39 d<br>6,5 = 7.4
**9**	6.55 d<br>1′,2′ = 7.7	4.50 dd<br>2′,1′ = 7.7<br>2′,3′ ≈ 4.3	4.14 dd<br>3′,2′ = 4.3<br>3′,4′ = 1.3	4.11 ddd<br>4′3′ = 1.3<br>4′,5′ = 4.2<br>4′,5″ = 3.6	3.37 dd<br>5′,4′ = 4.2<br>5′,5″= 10.8	3.205 dd<br>5″,4′ = 3.6<br>5″,5′ = 10.8	7.15 bd<br>5,6 = 7.5	8.06 bd<br>6,5 = 7.5
**10**	5.75 s<br>1′,2′ ≈ 0	4.11 d<br>2′,1′ ≈ 0	4.475 dd<br>3′,2′ = 4.3	4.575 ddd<br>4′3′ = 9.7	3.36 d (2H)<br>5′,4′ = 3.0		7.11 bd<br>5,6 = 7.5	8.36 d<br>6,5 = 7.5

		2′,3′ ≈ 4.3	*3′,4′ = 9.7*	*4′,5′ = 3.0*<br>*4′,5″ = 3.0*				
**12**	6.195 dd<br>*1′,2′ = 2.0*<br>*1′,2″ = 8.0*	2.28 dd<br>*2′,1′ = 2.0*<br>*2′,2″ = 14.9*<br>*2′,3′≈ 0*	4.28 dd<br>*3′,2′ ≈ 0.0*<br>*3′,2″ = 5.5*<br>*3′,4′ = 3.3*	4.22 dt<br>*4′3′ = 3.3*<br>*4′,5′ = 7.5*<br>*4′,5″ = 3.7*	3.455 dd<br>*5′,4′ = 7.5*<br>*5′,5″ = 10.3*	3.365 dd<br>*5″,4′ = 3.7*<br>*5″,5′ = 10.3*	7.20 bd<br>*5,6 = 7.5*	8.16 d<br>*6,5 = 7.5*
**13**<br>T =100 deg	5.91 s<br>*1′,2′ ≈ 0*	4.92 bd<br>*2′,1′ ≈ 0*<br>*2′,3′ ≈ 0*<br>*2′,3″ = 5.2*	1.96 dd<br>*3′,2′ ≈ 0.0*<br>*3′,3″ = 13.6*<br>*3′,4′ = 5.5*	4.39 m<br>*4′3′ = 5.5*<br>*4′,3″ = 11.2*<br>*4′,5′ = 2.9*<br>*4′,5″ = 4.2*	3.43 dd<br>*5′,4′ = 2.9*<br>*5′,5″ = 10.9*	3.32 dd<br>*5″,4′ = 4.2*<br>*5″,5′ = 10.9*	7.075 bd<br>*5,6 = 7.5*	8.23 d<br>*6,5 = 7.5*
**14**	6.21 d<br>*1′,2″ = 5.5*	**H-2″**<br>*4.57 td*<br>*2″,1 = 5.5*<br>*2″,3′ = 7.1*<br>*2″,3″=7.0*	1.94 ddd<br>*3′,2′ ≈ 0*<br>*3′,3″= 13.0*<br>*3′,4′ = 9.2*	4.12 m<br>*4′3′ = 9.2*<br>*4′3″ = 6.6*<br>*4′,5′= 5.0*<br>*4′,5″= 3.1*	3.27 dd<br>*5′,4′ = 5.0*<br>*5′,5″= 10.6*	3.23 dd<br>*5″,4′ = 3.1*<br>*5″,5′= 10.6*	7.15 bd<br>*5,6 = 7.5*	8.12 d<br>*6,5 = 7.5*
**15**	6.04 t<br>*1′,2′ = 5.1*<br>*1′,2″ = 5.1*	≈2.38 m	≈3.69 m	3.93 m	≈3.29 m	≈3.29 m	5.69 d<br>*5,6 = 7.2*	8.01 d<br>*6,5 = 7.2*

Table S1. ^{1}H NMR data of compounds **3-9, 10, 12-15** in DMSO. Coupling constants *J*(H,H) in Hz and in italics

Additional proton NMR data in compounds (only signal multiplicity and number of protons are given)

3 **2.3-O>C(CH$_3$)-P(=O,-OH)-O-CH$_2$-Pic:** 1.49 d, 3H; 4.87 dd + 4.84 dd, 2H; 8.09 d, 1H; 6.93 dd, 1H; 7.14 d, 1H; 3.79 s, 3H; **DMTr:** 7.34 m, 2H; 7.29 m, 2H; 7.22 m, 2H; 7.21 m, 2H; 7.20 m, 1H; 6.87 m, 2H; 6.85 m, 2H; 3.71 s, 3H; 3.70 s, 3H; **NH-Bz:** 11.32 br; 8.01 m, 2H; 7.63 m, 1H; 7.52 m, 2H

4 **2.3-O>C(C$_6$H$_5$)-P(=O,-OH)-O-CH$_2$-Pic:** 7.63 m, 2H; 7.30 m, 3H; 4.75 bd, 2H; 6.97 bd, 1H; 6.90 dd, 1H; 8.07 d, 1H; 3.75 s, 3H; **DMTr:** 7.32 m, 2H; 7.21 m, 2H; 7.20 m, 5H; 6.86 m, 2H; 6.83 m, 2H; 3.72 s, 3H; 3.70 s, 3H; **NH-Bz:** 11.32 bs; 8.01 m, 2H; 7.63 m, 1H; 7.52 m, 2H

5 **2.3-O>CH-CH$_2$-P(=O,-OH)-O-CH$_2$-Pic:** 5.47 bq**,** 1H; 5.31 bq, 1H; 2.02 bd, 1H; 1.92 bd, 1H; 4.83 um, 2H; 7.16 d, 1H; 6.91 br, 1H; 8.10 bd, 1H; 3.76 s, 3H; **DMTr:** 7.34 m, 2H; 7.27 m, 2H; 7.21 m, 4H; 7.18 m, 1H; 6.85 m, 2H; 6.83 m, 2H; 3.70 s, 3H; 3.68 s, 3H: **NH-Bz:** 11.34 br; 8.01 m, 2H; 7.52 m, 2H; 7.63

6 **2.3-O>CH-CH(O-CO-O-iBu)-P(=O,-OH)(-O-CH$_2$-Pic):** 5.44 bt, 1H; 4.88 m, 1H; 4.885 m, 2H; 7.12 bd, 1H; 6.96 dd, 1H; 8.12 d, 1H; 3.83 s, 3H; **DMTr:** 7.32 m, 2H; 7.26 m, 2H; 7.21 m, 2H; 7.19 m, 3H; 6.85 m, 2H; 6.82 m, 2H; 3.71 s, 3H; 3.69 s, 3H; **NH-Bz:** 11.32 br**;** 8.01 m, 2H; 7.52 m, 2H; 7.63 m, 1H

7 **O-CH$_2$-CH-P(=O,OH)-O-CH$_2$-Pic:** 3.93 m + 3.71 m, 2H; 3.74 m, 1H; 4.91 d, 2H; 7.14 bd, 1H; 6.90 dd, 1H; 8.11 d, 1H; 3.78 s, 3H; **DMTr**: 7.38 m, 2H; 7.35 m, 2H; 7.26 m, 1H, 7.25 m, 4H; 6.93 m, 4H; 3.75 s, 6H; **NH-Bz:** 11.34 br**;** 8.00 m, 2H; 7.52 m, 2H; 7.63 m, 1H

8 **O-CH$_2$-CH-P(=O,OH)-O-CH$_2$-Pic:** 3.94 m +3.61 m, 2H; 3.94 m, 1H; 4.93 dd + 4.85 dd, 2H; 7.11 d, 1H; 6.96 dd, 1H; 8.15 d, 1H; 3.79 s, 3H; **DMTr**: 7.38 m, 2H; 7.33 m, 2H; 7.26 m, 4H; 7.22 m, 1H; 6.90 m, 4H; 3.73 s, 3H; 3.72 s, 3H; **NH-Bz:** 11.27 br**;** 8.00 m, 2H; 7.52 m, 2H; 7.63 m, 1H

9 **O-CH$_2$-CH-P(=O,OH)-O-CH$_2$-Pic:** 3.94 m + 3.71 m, 2H; 3.95 m , 1H; 4.86 dd + 4.82 dd, 2H; 7.10 bd, 1H; 6.90 dd, 1H; 8.09 d, 1H; 3.78 s, 3H; **DMTr**: 7.38 m, 2H; 7.35 m, 2H; 7.26 m, 5H; 6.93 m, 4H; 3.75 s, 6H; **NH-Bz:** 11.33 br; 8.00 m, 2H; 7.63 m, 1H; 7.52 m, 2H

10 **O-CH$_2$-CH-P(=O,OH)-O-CH$_2$-Pic:** 3.75 m + 3.63 m, 2H; 3.64 m , 1H; 4.94 dd + 4.89 dd, 2H; 7.13 bd, 1H; 6.94 dd, 1H; 8.12 d, 1H; 3.79 s, 3H; **DMTr**: 7.38 m, 2H; 7.34 m, 2H; 7.26 m, 5H; 6.91 m, 4H; 3.79 s, 3H; 3.75 s, 3H; **NH-Bz:** 11.28 bs; 8.00 m, 2H; 7.62 m, 1H; 7.52 m, 2H

12 **O-CH$_2$-P(=O)OH-Pic:** 3.30 dd + 3.26 dd, 2H; 4.71 dd + 4.68 dd, 2H; 8.09 d, 1H; 6.99 bd, 1H; 6.89 dd, 1H; 3.71 s, 3H; **DMTr**: 7.40 m, 2H; 7.28 m, 4H; 7.25 m, 2H; 7.18 m, 1H; 6.84 m, 4H; 3.706 s, 3H; 3.703 s, 3H; **NH-Bz:** 11.15 br; 7.98 m, 2H; 7.62 m, 1H; 7.51 m, 2H; **H-2″:** 2.50 dd; 2″,1 = 8.0; 2″,2′= 14.9; 2″,3′ = 5.5

13 **O-CH$_2$-P(=O)OH-Pic:** 3.85 dd + 3.69 dd, 2H; 4.97 d, 2H; 8.04 d, 1H; 7.21 bd, 1H; 6.87 dd, 1H; 3.71 s, 3H; **H-3″:** 2.05 ddd, 3″,2′ ≈ 5.2, 3″,3′ = 13.6, 3″,4′ = 11.2; **DMTr**: 7.39 m, 2H; 7.28 m, 4H; 7.34 m, 2H; 7.25 m, 1H; 6.91 m, 4H; 3.77 s, 6H; **NH-Bz:** 10.90 br; 8.01 m, 2H; 7.61 m, 1H; 7.51 m, 2H

14 **O-CH$_2$-P(=O)OH-Pic:** 3.29 m, 2H; 4.77 m, 2H; 8.03 d, 1H; 7.01 bd, 1H; 6.83 dd, 1H; 3.71 s, 3H; **H-3″:** 2.30 ddd; 3″,2″ ≈ 7.0; 3″,3′ = 13.0; 3″,4′ = 6.6; **DMTr:** 7.38 m, 2H; 7.33 m, 2H; 7.25 m, 4H; 7.24 m, 1H; 6.91 m, 4H; 3.751 s, 3H; 3.748 s, 3H; **NH-Bz:** 11.16 br; 7.98 m, 2H; 7.62 m, 1H; 7.51 m, 2H

15 **H-2″:** ≈2.38 m; **S-CH$_2$-P(=O)(OH)-O-CH$_2$-Pic:** 2.81 m, 2H; 4.86 bd, 8.10 d, 1H; 7.13 bd, 1H; 6.93 bdd, 1H; 3.76 s, 3H; **DMTr:** 7.38 m, 2H; 7.30 m, 2H; 7.25 m, 4H; 7.20 m, 1H; 6.88 m, 4H; 3.719 s, 3H; 3.717 s, 3H; **N=CH-N(CH$_2$-CH$_2$-CH$_2$-CH$_3$)$_2$:** 8.61 s; 3.47 m, 2H; 3.41 m, 2H; 1.54 m, 4H; 1.27 m, 4H; 0.902 t, 3H; 0.899 t, 3H

Compound	H-1’	H-2’	H-3’	H-4’	H-5’	H-5”	H-5	H-6
**25**<br>DIAs 50:50	5.99 d<br>*1’,2’ = 1.7*	5.03 dd<br>*2’,1’ = 1.7*<br>*2’,3’ ≈ 6.4*	4.845 dd<br>*3’,2’ = 6.4*<br>*3’,4’ = 3.8*	4.52 dt<br>*4’3’ = 3.8*<br>*4’,5’ = 5.6*<br>*4’,5” = 3.8*	3.82 dd<br>*5’,4’ = 5.6*<br>*5’,5” = 11.3*	3.97 dd<br>*5”,4’ = 3.8*<br>*5”,5’ = 11.3*	7.29 bd<br>*5,6 = 7.2*	8.225 bd<br>*6,5 = 7.2*
	5.96 d<br>*1’,2’ = 1.7*	5.00 dd<br>*2’,1’ = 1.7*<br>*2’,3’ ≈ 6.4*	4.84 dd<br>*3’,2’ = 6.4*<br>*3’,4’ = 3.8*	4.455 dt<br>*4’3’ = 3.8*<br>*4’,5’ = 5.7*<br>*4’,5” = 3.8*				8.21 bd<br>*6,5 = 7.2*
**26**<br>DIAs 68:32	5.905 d<br>*1’,2’ = 2.0*	4.98 dd<br>*2’,1’ = 2.0*<br>*2’,3’ ≈ 6.3*	4.84 dd<br>*3’,2’ = 6.3*<br>*3’,4’ = 4.3*	4.275 ddd<br>*4’3’ = 4.3*<br>*4’,5’ = 7.0*<br>*4’,5” = 6.4*	3.40 dd<br>*5’,4’ = 7.0*<br>*5’,5” = 10.8*	3.185 dd<br>*5”,4’ = 6.4*<br>*5”,5’ = 10.8*	7.29 bd<br>*5,6 = 7.5*	8.20 bd<br>*6,5 = 7.5*
	5.908 d<br>*1’,2’ = 1.6*	5.01 dd<br>*2’,1’ = 1.6*<br>*2’,3’ ≈ 6.3*	4.76 dd<br>*3’,2’ = 6.3*<br>*3’,4’ = 4.3*	4.395 ddd<br>*4’3’ = 4.3*<br>*4’,5’ = 7.0*<br>*4’,5” = 6.4*	3.345 dd<br>*5’,4’ = 7.0*<br>*5’,5” = 10.8*	3.175 dd<br>*5”,4’ = 6.4*<br>*5”,5’ = 10.8*		
**27**<br>DIAs 50:50	5.93 d<br>*1’,2’ = 1.3*	5.14 dd<br>*2’,1’ = 1.3*<br>*2’,3’ ≈ 6.3*	4.845 dd<br>*3’,2’ = 6.4*<br>*3’,4’ = 3.8*	4.315 dt<br>*4’3’ = 3.8*<br>*4’,5’ = 6.8*<br>*4’,5” = 3.8*	3.327 dd<br>overlap	3.10 dd<br>*5”,4’ = 3.8*<br>*5”,5’ = 10.4*	7.28 bd<br>*5,6 = 7.5*	8.245 bd<br>*6,5 = 7.5*
	5.83 d<br>*1’,2’ = 1.5*	5.05 dd<br>*2’,1’ = 1.6*<br>*2’,3’ ≈ 6.2*	4.87 dd<br>*3’,2’ = 6.4*<br>*3’,4’ = 3.8*	4.465 dt<br>*4’3’ = 3.8*<br>*4’,5’ = 6.6*<br>*4’,5”=3.8*	3.327 dd<br>overlap	3.16 dd<br>*5”,4’ = 3.8*<br>*5”,5’ = 10.4*		8.18 bd<br>*6,5 = 7.5*
**39**	6.07 dd<br>*1’,2’ = 3.4*<br>*1’,2” = 7.1*	2.52 ddd<br>*2’,1’ = 3.4*<br>*2’,2” = 13.8*<br>*2’,3’ ≈ 7.7*	3.78 m<br>*3’,2’ = 7.7*<br>*3’,2”= 9.4*<br>*3’,4’ = 8.6*	3.91 ddd<br>*4’3’ = 8.6*<br>*4’,5’ = 2.6*<br>*4’,5” = 4.2*	3.33 dd<br>*5’,4’ = 2.6*<br>*5’,5” = 10.8*	3.27 dd<br>*5”,4’ = 4.2*<br>*5”,5’ = 10.8*	5.285 dd<br>*5,6 = 8.0*<br>*5,NH = 2.1*	7.82 d<br>*6,5 = 8.0*
**40**	6.06 dd<br>*1’,2’ = 3.8*<br>*1’,2” = 6.2*	≈2.44 m	≈3.75 m	3.955 ddd<br>*4’3’ = 8.9*<br>*4’,5’ = 2.5*<br>*4’,5” = 3.8*	3.395 dd<br>*5’,4’ = 2.5*<br>*5’,5” = 11.0*	3.31 dd<br>*5”,4’ = 3.8*<br>*5”,5’ = 11.0*	5.605 d<br>*5,6 = 7.1*	8.04 d<br>*6,5 = 7.1*

Table S2. ^{1}H NMR data of compounds **25-27, 39, 40** in DMSO. Coupling constants *J*(H,H) in Hz and in italics

Additional proton NMR data in compounds (only signal multiplicity and number of protons are given)

25 **2.3-O>CH-CHOH-P(=O)(-O-CH$_2$-CH$_3$)$_2$:** 5.30 dd, 1H; 3.99 m, 1H; 5.38 t, 1H; 4.12-4.06 m, 4H; 1.24 t, 6H; 5.27 dd, 1H; 3.99 m, 1H; 5.36 t, 1H; 4.12-4.06 m, 4H; 1.21 t, 6H; **TBDMS:** 7.63 m, 2H; 7.43 m, 2H; 7.47 m, 1H; 0.99 s , 9H; 7.60 m, 2H; 7.39 m, 2H,; 7.43 m, 1H; 0.99 s, 9H; **NH-Bz:** 11.29 br; 8.00 m, 2H; 7.63 m, 1H; 7.52 m, 2H

26 **2.3-O>CH-CH$_2$-P(=O)(-O-CH$_2$-CH$_3$)$_2$:** 5.43 q, 1H; 2.28 m, 2H; 4.02 m, 4H; 1.23 t, 3H; 1.22 t, 3H; 5.25 q, 1H; 2.37 m, 2H; 4.02 m, 4H; 1.22 t, 3H; 1.24 t, 3H; **DMTr:** 7.37 m, 2H; 7.28 m, 2H; 7.25 m, 4H; 7.21 m, 1H; 6.86 m, 4H; 3.72 s, 3H; 3.70 s, 3H; **NH-Bz:** 11.32 br; 8.01 m, 2H; 7.63 m, 1H; 7.52 m, 2H

27 **2.3-O>CH-CH(O-CO-O-iBu)(-P(=O)(-O-CH_2-CH_3)$_2$):** 5.49 dd, 1H; 5.22 dd, 1H; 4.17 m, 4H; 1.27 t, 3H; 1.24 t, 3H; 4.03 dd, 1H; 4.00 dd, 1H; 1.96 m, 1H; 0.91 d, 3H; 0.87 d, 3H; 5.47 dd, 1H; 5.22 dd, 1H; 4.10 m, 4H; 1.23 t, 3H; 1.19 t, 3H; 3.95 m, 2H; 1.91 m, 1H; 0.91 d, 3H; 0.84 d, 3H; **DMTr:** 7.34 m, 2H; 7.27 m, 2H; 7.23 m, 2H; 7.21 m, 3H; 6.86 m, 4H; 3.72 s, 3H; 3.71 s, 3H; **NH-Bz:** 11.32 br; 8.01 m, 2H; 7.63 m, 1H; 7.52 m, 2H

39 **H-2”:** 2.42 ddd, 2”,1 = 7.1; 2”,2’= 13.8; 2”,3’ = 9.4; **S-CH_2-P(=O)(-iPr)$_2$**: 2.87 dd + 2.91 dd, 2H; 4.55 m, 2H; 1.17 d, 1.213 d, 1.215 d, 1.218 d (4x CH_3); **DMTr:** 7.38 m, 2H; 7.31 m, 2H; 7.25 m, 4H; 7.24 m, 1H; 6.89 m, 4H; 3.74 s, 6H

40 **H-2”:** ≈2.44 m; **S-CH_2-P(=O)(-iPr)$_2$**: 2.89 dd + 2.85 dd, 2H; 4.55 m, 2H; 1.210 d, 1.209 d, 1.206; 1.164 d, (4x CH_3); **DMTr:** 7.40 m, 2H; 7.32 m, 2H; 7.27 m, 4H; 7.25 m, 1H; 6.89 m, 4H; 3.74 s, 6H; **N=CH-N(CH_2-CH_2-CH_2-CH_3)$_2$:** 8.62 s**;** 3.47 m,2H; 3.42 m, 2H; 1.54 m, 4H; 1.27 m, 4H; 0.907 t, 3H; 0.903 t, 3H

Compound	C-1‘	C-2‘	C-3‘	C-4‘	C-5‘	C-2	C-4	C-5	C-6	^{31}P
**3**	94.03	86.68	82.44	87.04	64.24	154.31	163.83	96.33	147.14	10.80
**4**	94.00	86.11	82.79	86.21	64.33	154.37	163.79	96.30	147.50	9.25
**5**	94.85	85.03	80.76	87.11	64.67	154.37	163.76	96.38	147.09	14.34
DIAs 50:50	94.58	82.96	81.45	85.03	64.22			96.52	147.78	
**6**	94.01	85.59	80.66	86.84	64.18	154.38	163.90	96.37	147.71	6.77
**7**	81.00	76.20	74.33 d (10.0)	82.88	63.98	154.70	163.38	97.01	144.57	9.89
**8**	89.55	76.21	59.34 d (9.2)	76.12	61.89	154.27	163.40	96.18	144.95	11.48
**9**	81.78	75.74	73.45	82.18	63.87	154.87	163.43	96.88	144.91	10.59
**10**	89.83	77.00	68.95	74.81	61.96	154.14	163.33	96.27	145.12	9.52
**12**	85.73	37.04	78.77 d (8.9)	83.62	62.22	162.95	154.71	96.15	146.14	13.53
**13** T=100 deg	90.57	84.59 d (10.4)	31.07	80.06	63.34	162.69	n.d.	95.81	143.98	13.02
**14**	84.97	79.23 d (7.9)	32.41	75.90	64.41	162.96	154.68	95.72	146.35	13.00
**15**	85.27	39.76	41.50	84.17	62.64	155.12	171.28	101.48	142.08	13.81
**25**	93.59	85.54	81.11	87.07	64.19	154.42	163.78	96.31	147.04	21.18
DIAs 50:50	93.39	85.46	81.07	86.89	64.17				146.90	21.15
**26**	94.07	83.50	81.09	84.27	64.33	154.36	163.84	96.47	147.61	25.23
DIAs 68:32	93.94	85.45	81.56	86.75	64.00			96.29	147.26	25.21
**27**	94.06	85.94	81.75	86.76	64.03	154.34	163.95	96.35	147.90	15.40
DIAs 50:50	93.47	85.71		85.76	64.00	154.30	163.86		147.25	15.31
**39**	84.15	38.73	41.70 d (5.0)	83.52	62.51	150.49	163.22	101.45	140.61	23.26
**40**	85.16	39.87	41.22 d (4.6)	83.87	62.18	155.07	171.32	101.50	141.89	23.33

Table S3. ^{13}C and ^{31}P NMR data of compounds **3-9, 10, 12-15, 25-27, 39, 40** in DMSO. Coupling constants *J*(P,C) in Hz and number of equivalent carbon atoms are given in brackets

Additional carbon NMR data in compounds:

3 **2.3-O>C(CH_3)-P(=O,-OH)-O-CH_2-Pic:** 113.75 d (*187.2*); 23.70 d (*17.5*); 61.22 d (4.4); 151.34; 107.68; 156.63; 110.35; 139.40; 56.19; **DMTr:** 158.28; 158.23; 144.81; 135.51; 135.46; 129.87 (2C); 129.75 (2C); 128.00 (2C); 127.83 (2C); 126.68; 113.36 (4C); 85.95; 55.14; 55.11; **NBz:** 167.80; 133.97; 132.91; 128.61 (4C).

4 **2.3-O>C(C_6H_5)-P(=O,-OH)-O-CH_2-Pic:** 113.65 d (185.0); 141.22 d (15.8); 126.66, 2C; 127.12, 2C; 127.46; 61.90 d (5.0); 151.13 d (4.0); 108.60; 156.65; 110.38; 139.40; 56.13; **DMTr**: 158.29; 158.23; 144.83; 135.52; 135.43; 129.87 (2C); 129.73 (2C); 127.99 (2C); 127.81 (2C); 126.68; 113.36 (2C); 113.34 (2C); 85.94; 55.14; 55.10; **NBz:** 167.48; 133.25; 132.93; 128.61 (4C).

5 **2.3-O>CH-CH_2-P(=O,-OH)-O-CH_2-Pic:** 102.27; 33.96 d (130.0); 60.54; 151.05; 108.96; 156.66; 110.34; 139.40; 56.17; 102.57; 33.36 d (130.0); 60.45; 151.05; 108.82; 156.66; 110.23; 139.40; 56.15*;* **DMTr**: 158.26; 158.23; 158.19; 144.97; 144.79; 135.60; 135.54; 135.48; 135.42; 129.89; 129.75; 129.73; 127.96; 127.82; 126.87; 126.83; 113.33; 113.30; 55.13; 55.10; 55.08; **NBz:** 167.54; 133.28; 132.96; 128.63 (4C).

6 **2.3-O>CH-CH(O-CO-O-iBu)-P(=O,-OH)(-O-CH_2-Pic):** 105.64 d (5.4); 72.30 d (148.0); 61.36 d (3.7); 150.91; 108.68; 156.70; 110.35; 139.44; 56.16; **DMTr**: 158.26; 158.20; 144.90; 135.51; 135.42; 129.86 (2C); 129.72 (2C); 127.93 (2C); 127.77 (2C); 126.85; 113.30 (2C); 113.2(2C); 55.13; 55.09; **NBz:** 167.49; 133.23; 132.96; 128.62 (4C).

7 **O-CH_2-CH-P(=O,OH)-O-CH_2-Pic:** 62.18 d (5.7); 72.79 d (152.5); 61.36 d (4.3); 150.99 d (5.2); 108.62; 156.65; 110.31; 139.42; 56.15; **DMTr**: 158.41; 158.40; 144.57; 135.37; 135.12; 129.90 (4C); 128.21 (2C); 127.84 (2C); 127.08; 113.57 (2C); 113.56 (2C); 55.28; 55.23; **NBz:** 167.65; 133.26; 132.98; 128.70 (2C); 128.61 (2C).

8 **O-CH_2-CH-P(=O,OH)-O-CH_2-Pic:** 66.11; 67.39 d (153.6); 61.43 d (4.3); 150.81; 108.99; 156.89; 110.31; 139.53; 56.25; **DMTr**: 158.32 (2C); 144.52; 135.63; 135.35; 129.89 (2C); 129.80 (2C); 128.14 (2C); 127.94 (2C); 127.00; 113.50 (4C); 55.11 (2C); **NBz:** 167.44; 133.29; 132.90; 128.62 (4C).

9 **O-CH_2-CH-P(=O,OH)-O-CH_2-Pic:** 65.75 d (4.0); 68.01 d (151.6); 60.99 d (4.1); 151.03 d (5.6); 108.56; 156.58; 110.37; 139.38; 56.16; **DMTr**: 158.43; 158.39; 144.58; 135.43; 135.19; 129.90 (2C); 129.87 (2C); 128.19 (2C); 127.86 (2C); 127.08; 113.55 (4C); 55.23 (2C); **NBz:** 167.49; 133.19; 132.96; 128.68 (2C); 128.61 (2C).

10 **O-CH_2-CH-P(=O,OH)-O-CH_2-Pic:** 61.24 d (5.7); 73.23 d (152.8); 61.38 d (4.4); 150.92 d (5.4); 108.79; 156.63; 110.27; 139.49; 56.18; **DMTr**: 158.37; 158.35; 144.62; 135.63; 135.34; 129.95 (4C); 128.15 (2C); 128.00 (2C); 127.07; 113.49 (4C); 55.18 (2C); **NBz:** 167.60; 133.31; 132.91; 129.83 (2C); 128.60 (2C).

12 **O-CH_2-P(=O)(OH)-O-CH_2-Pic:** 65.45 d (154.9); 60.79 d (4.3); 150.85 d (5.2); 108.81; 156.49; 110.07; 139.35; 56.04; **DMTr**: 158.19 (2C); 145.18; 135.74; 135.68; 129.89 (2C); 128.58 (2C); 127.92 (2C); 127.86 (2C); 126.76; 113.28 (4C); 85.74; 55.09 (2C); **NBz:** 167.18; 133.74; 132.77; 128.58 (2C); 128.56 (2C).

13 **O-CH_2-P(=O)(OH)-O-CH_2-Pic:** 66.12 d (155.8); 60.37 d (4.5); 153.94;109.07; 156.46; 109.94; 138.91; 55.78; **DMTr:** 158.15 (2C); 144.12; 135.41; 135.33; 129.45 (2C); 129.41 (2C); 127.68 (2C); 127.52 (2C); 126.53; 113.17 (4C); 86.00; 54.88 (2C); **NBz**: 167.57; 133.28; 132.16; 128.03 (2C); 128.02 (2C).

14 **O-CH_2-P(=O)(OH)-O-CH_2-Pic:** 67.60 d (153.7); 61.11 d (4.3); 151.00;108.56; 156.49; 110.16; 139.32; 56.06; **DMTr**: 158.30 (2C); 144.76; 135.69; 135.59; 129.87 (2C); 129.83 (2C); 128.08 (2C); 127.94 (2C); 126.96; 113.43 (4C); 85.83; 54.18 (2C); **NBz:** 166.37; 133.43; 132.79; 128.59 (2C); 128.56 (2C).

15 **S-CH_2-P(=O)(OH)-O-CH_2-Pic:** 46.64; 61.10; 150.89; 108.76; 156.75; 110.39; 139.47; 56.20; **DMTr**: 158.28 (2C); 144.73; 135.58; 135.39; 129.95 (4C); 128.09 (2C); 127.97 (2C); 126.92; 113.44 (4C); 86.06; 55.20; 55.18; **N=CH-N(CH_2-CH_2-CH_2-CH_3)$_2$:** 157.71; 51.37; 44.87; 30.59; 28.76; 19.70; 19.33; 13.82; 13.76.

25 **2.3-O>CH-CHOH-P(=O)(-O-CH_2-CH_3)$_2$:** 106.30 d (9.7);67.44 d (160.9); 62.54 d (7.4); 61.88 d (6.9); 16.56 d (5.4); 16.49 d (5.4); 106.20 d (9.8); 67.10 d (161.3); 62.46 d (7.4); 61.83 d (6.9); 16.50d (5.4); 16.49 d (5.4); **TBDMS:** 135.26 4C); 132.78; 132.63; 130.15; 130.13; 128.13; 128.07; 18.94; 26.80 (3C); **NBz:** 167.50; 133.21; 132.96; 128.63 (4C).

26 **2,3-O>CH-CH_2-P(=O)(-O-CH_2-CH_3)$_2$:** 100.19; 30.70 d (137.6); 61.51 d, 2C (5.9); 16.38 d, 2C (5.0); 102.90; 31.04 d (137.7); 61.51 d, 2C (5.9); 16.38 d, 2C (5.0); **DMTr**: 158.29 (2C); 144.92; 135.55; 135.49; 129.89 (4C); 129.79 (2C); 127.99 (2C); 126.88; 113.35 (4C); 85.88; 55.15; 55.12; **NBz:** 167.51; 133.19; 132.97; 128.62 (4C).

27 **2,3-O>CH-CH(O-CO-O-iBu)(-P(=O)(-O-CH_2-CH_3)$_2$**: 103.98 d (6.7); 70.01 d (163.5); 63.49 d (6.3); 63.38 d (6.3); 16.47 d (5.5); 16.32 d (5.5); 154.39; 74.58; 27.48; 18.69; 18.65; 103.86 d (6.4); 69.87 d (163.0); 63.82 d (5.9); 62.86 d (5.9); 16.32 d (5.6); 16.31 d (5.6); 154.36; 74.58; 27.48; 18.69; 18.65; **DMTr**: 158.30; 158.28; 158.26; 158.22; 144.90; 144.85; 135.49; 135.44; 135.39; 129.89; 129.88; 129.78; 129.72; 127.98; 127.94; 127.75; 127.77; 126.90; 126.88; 113.35; 113.33; 113.32; 113.28; 55.15; 55.12; 55.10; **NBz:** 167.50; 133.49; 132.98; 128.63 (4C).

39 **S-CH_2-P(=O)(-iPr)$_2$:** 24.14 d (146.8); 70.51 d (6.5); 70.49 d (6.5); 23.98 d (3.7), 23.96 d (3.8), 23.85 d (4.8), 23.80 d (4.9) (4x CH_3); **DMTr**: 158.33; 158.22; 144.75; 135.40; 135.28; 129.98 (4C); 128.04 (2C); 127.92 (2C); 126.97; 113.39 (2C); 113.32 (2C); 86.09; 55.21 (2C).

40 **S-CH_2-P(=O)(-iPr)$_2$**: 24.18 d (146.7); 70.48 d (6.4); 70.44 d (6.4); 23.97 d (3.6), 23.94 d (3.7), 23.84 d (4.8), 23.78 d (4.9) (4x CH_3); **DMTr**: 158.33 (2C); 144.64; 135.42; 135.26; 130.02 (2C); 128.97 (2C); 128.06 (2C); 127.97 (2C); 126.99; 113.41 (4C); 86.15; 55.20 (2C); **N=CH-N(CH_2-CH_2-CH_2-CH_3)$_2$:** 157.68; 51.33; 44.85; 30.57; 28.73; 19.66; 19.30; 13.89; 13.73.

Purity data of prepared monomers

Purity of prepared monomers was determined by LC-MS performed on Waters Alliance HPLC System with MS SQ Detector 2 (Waters, Milford, USA) using LUNA C18 column (Phenomenex, 100 × 4.6 mm, 3 µm) and 10 mM ammonium acetate buffer pH 6.8 with linear gradient of acetonitrile (0 to 100% in 20 min; or 2.5% to 100% in 15 min, flow rate 1 mL/min, column temperature 40°C, injection volume 5µL). The analytes were detected by PDA detector (254 nm channel), and MS detector (ESI scan). Original software (MassLynx) was used for instrument control and data acquisition. MestReNova software was used for data analysis.

Monomer	Purity (%)	Gradient
**1**	95	2.5%B-100%B in A, 15 min
**2**	99	0%B-100%B in A, 20 min
**3**	96	0%B-100%B in A, 20 min
**4**	96	0%B-100%B in A, 20 min
**5**	98	0%B-100%B in A, 20 min
**6**	99	0%B-100%B in A, 20 min
**7**	98	0%B-100%B in A, 20 min
**8**	95	0%B-100%B in A, 20 min
**9**	95	0%B-100%B in A, 20 min
**10**	99	0%B-100%B in A, 20 min
**11**	97	0%B-100%B in A, 20 min
**12**	96	0%B-100%B in A, 20 min
**13**	98	0%B-100%B in A, 20 min
**14**	95	0%B-100%B in A, 20 min
**15**	95	0%B-100%B in A, 20 min
**16**	99	2.5%B-100% B in A, 15 min

Table S4. Purity data of prepared monomers. LC-MS analysis on LUNA C18 column (Phenomenex, 100 × 4.6 mm, 3 µm); column temperature 40 °C; injection volume 5µL; flow rate 1 mL/min; mobile phases: A, 10 mM ammonium acetate buffer pH 6.8 and B, acetonitrile.

LC-MS chromatograms of prepared monomers

Monomer **1**

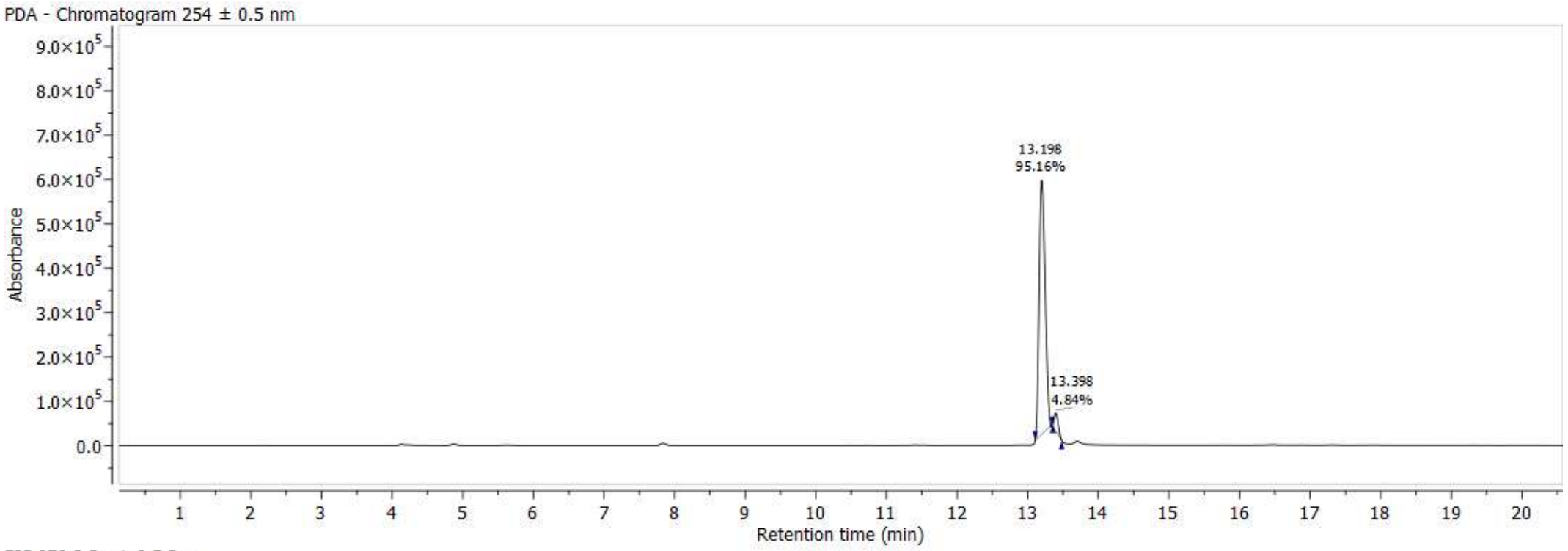

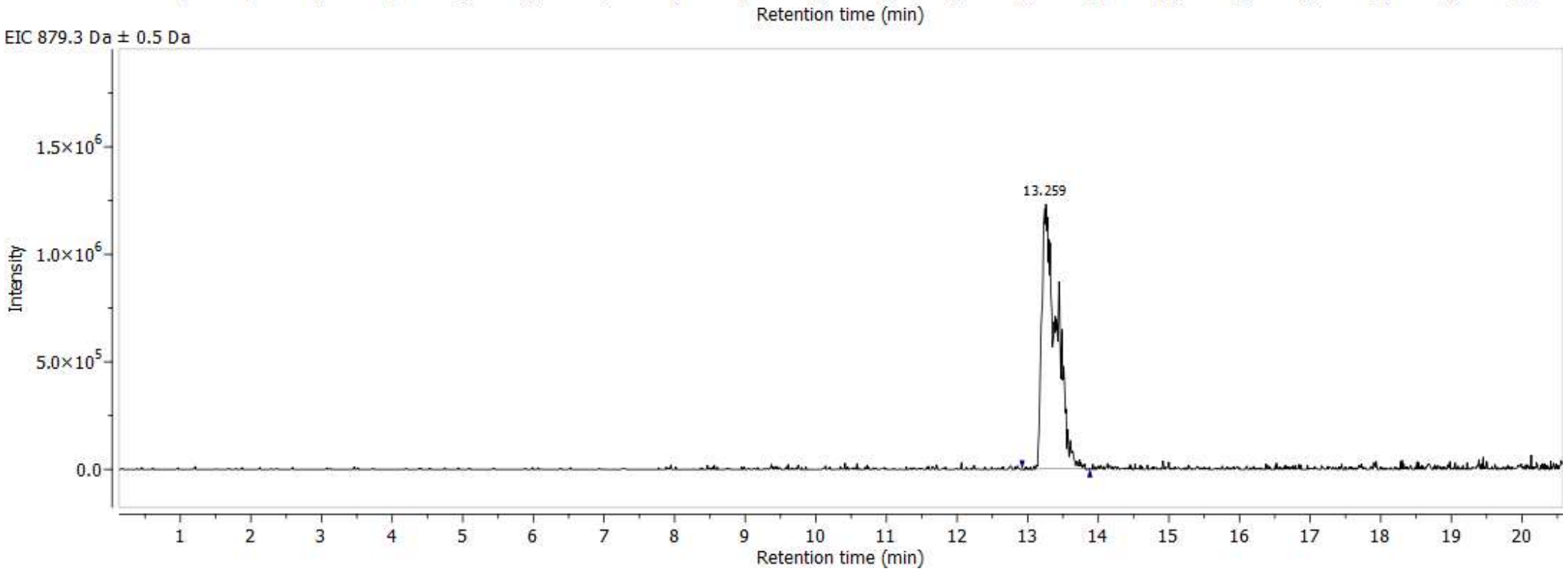

Monomer **2**

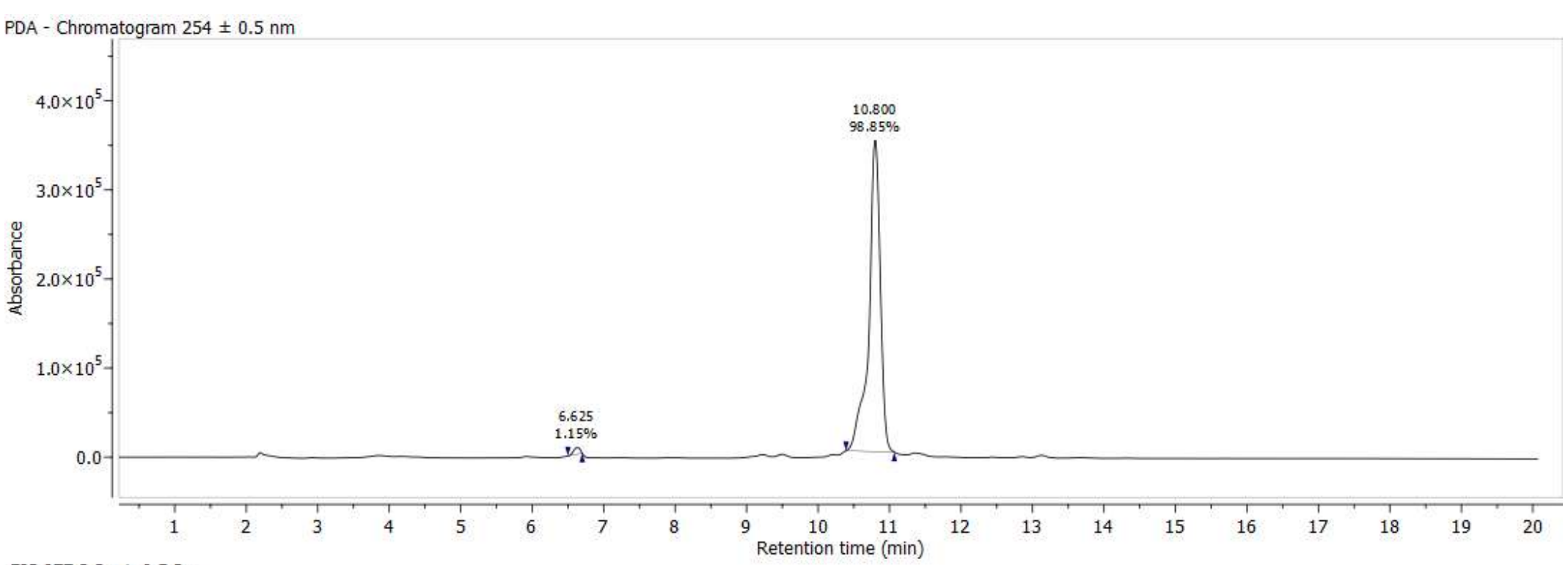

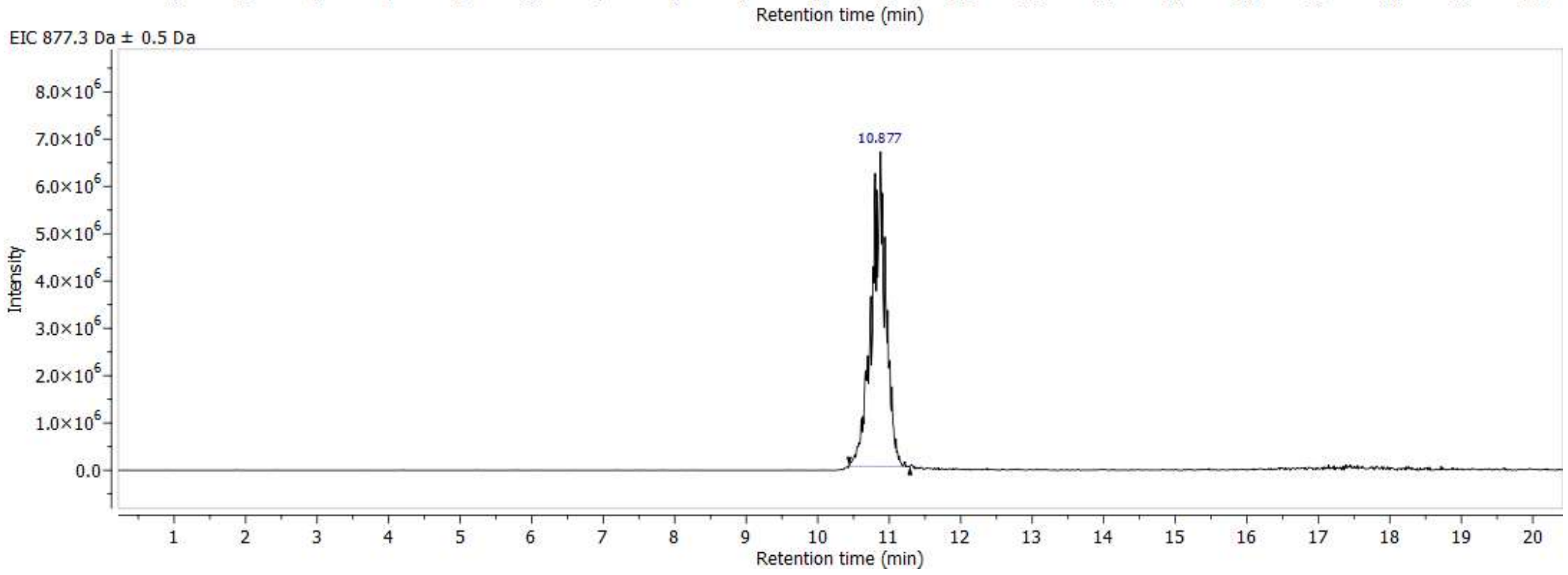

Monomer **3**

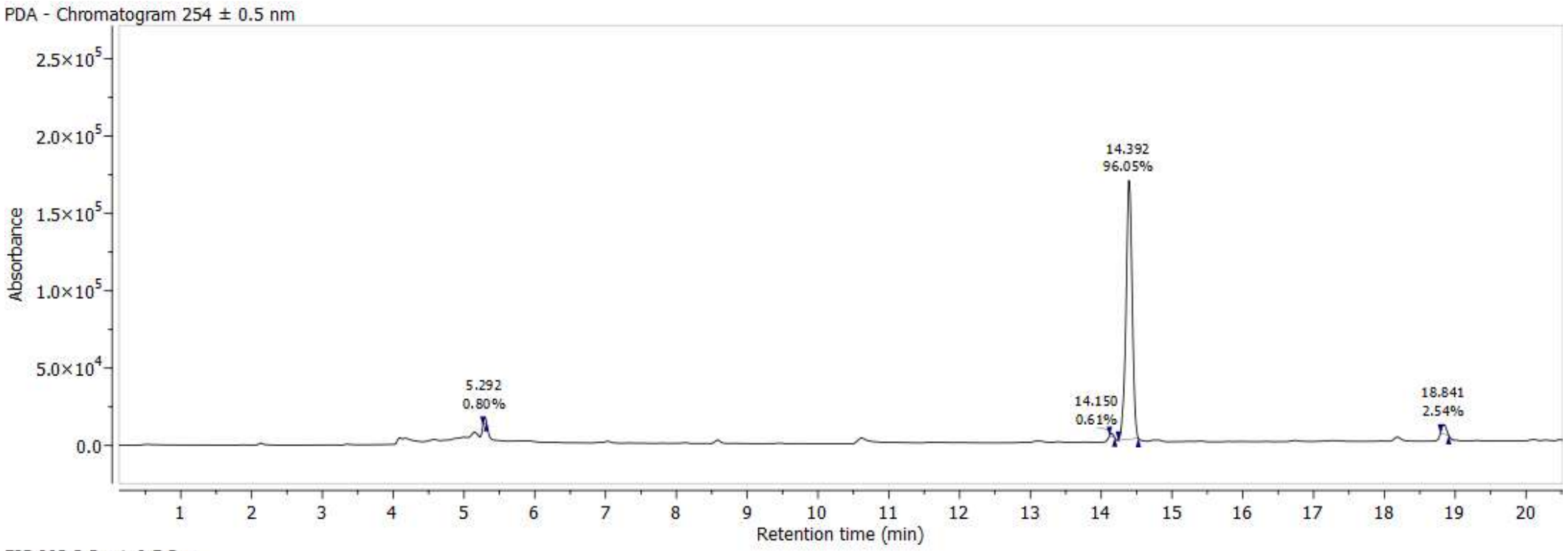

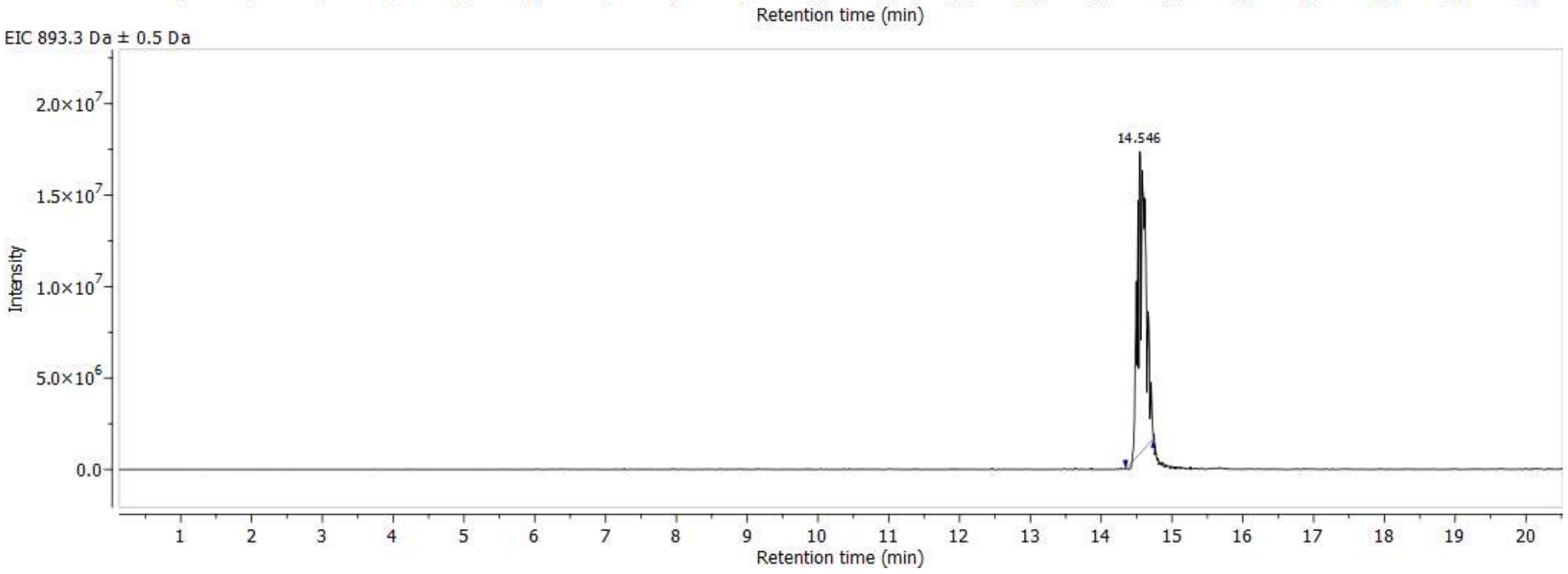

Monomer **4**

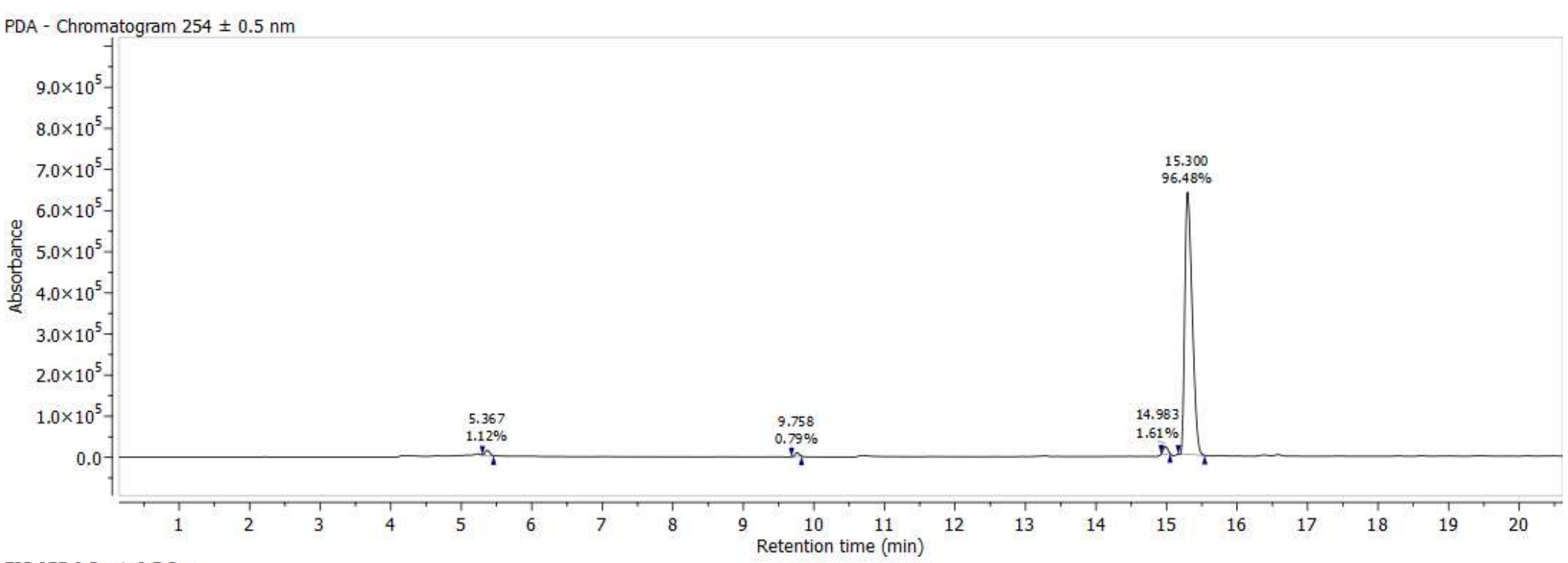

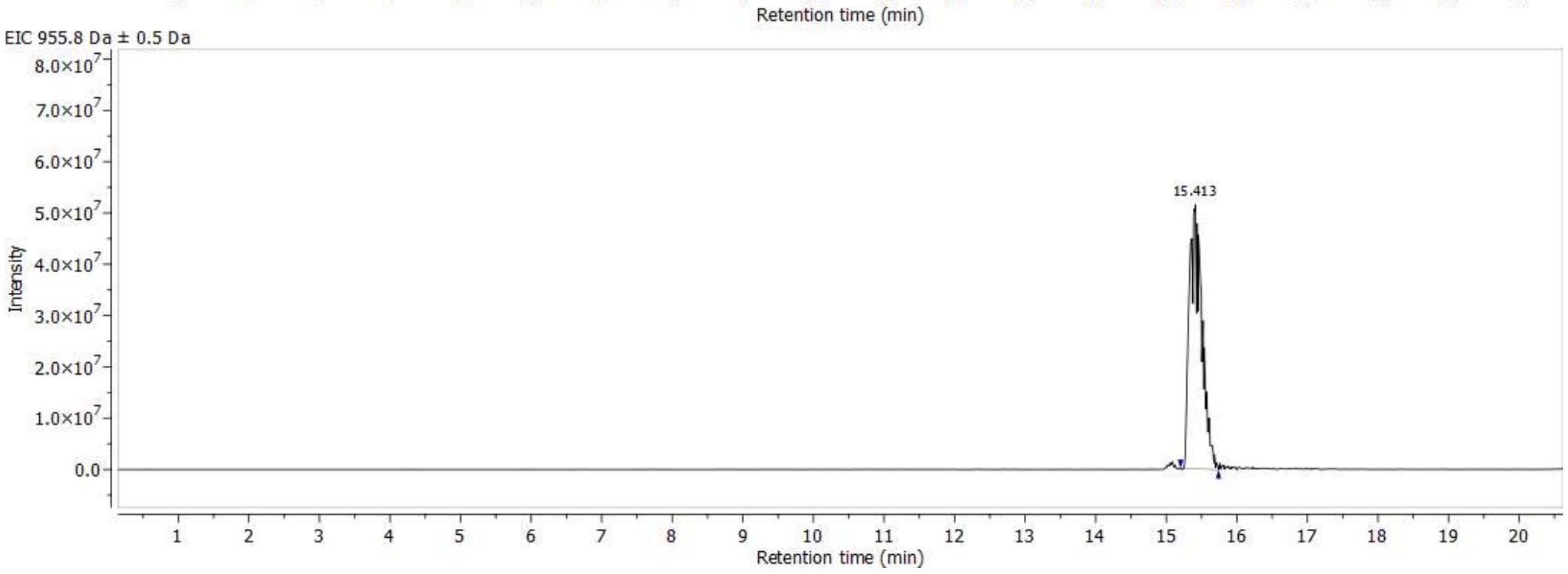

Monomer **5**

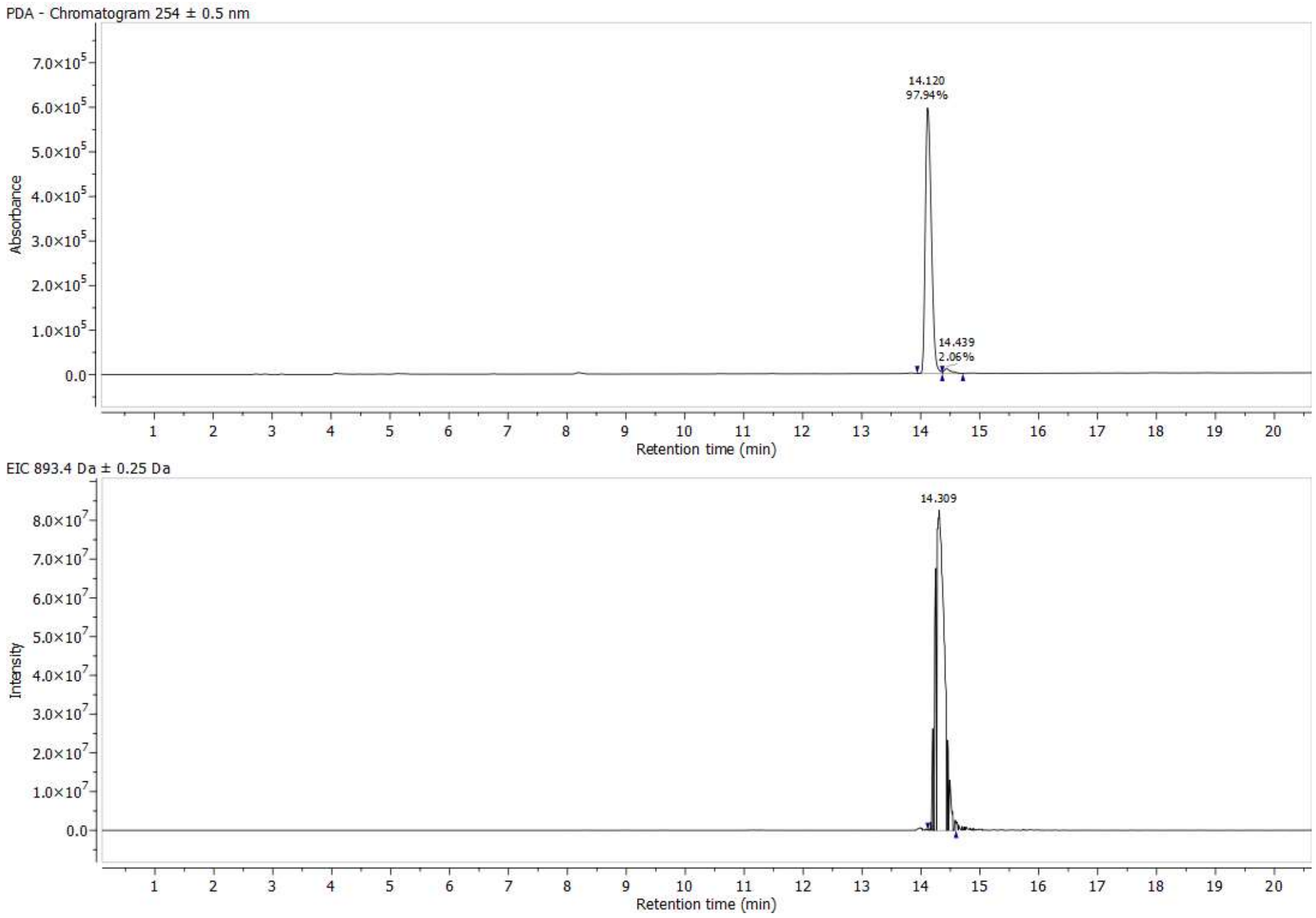

Monomer **6** (diastereoisomeric mixture)

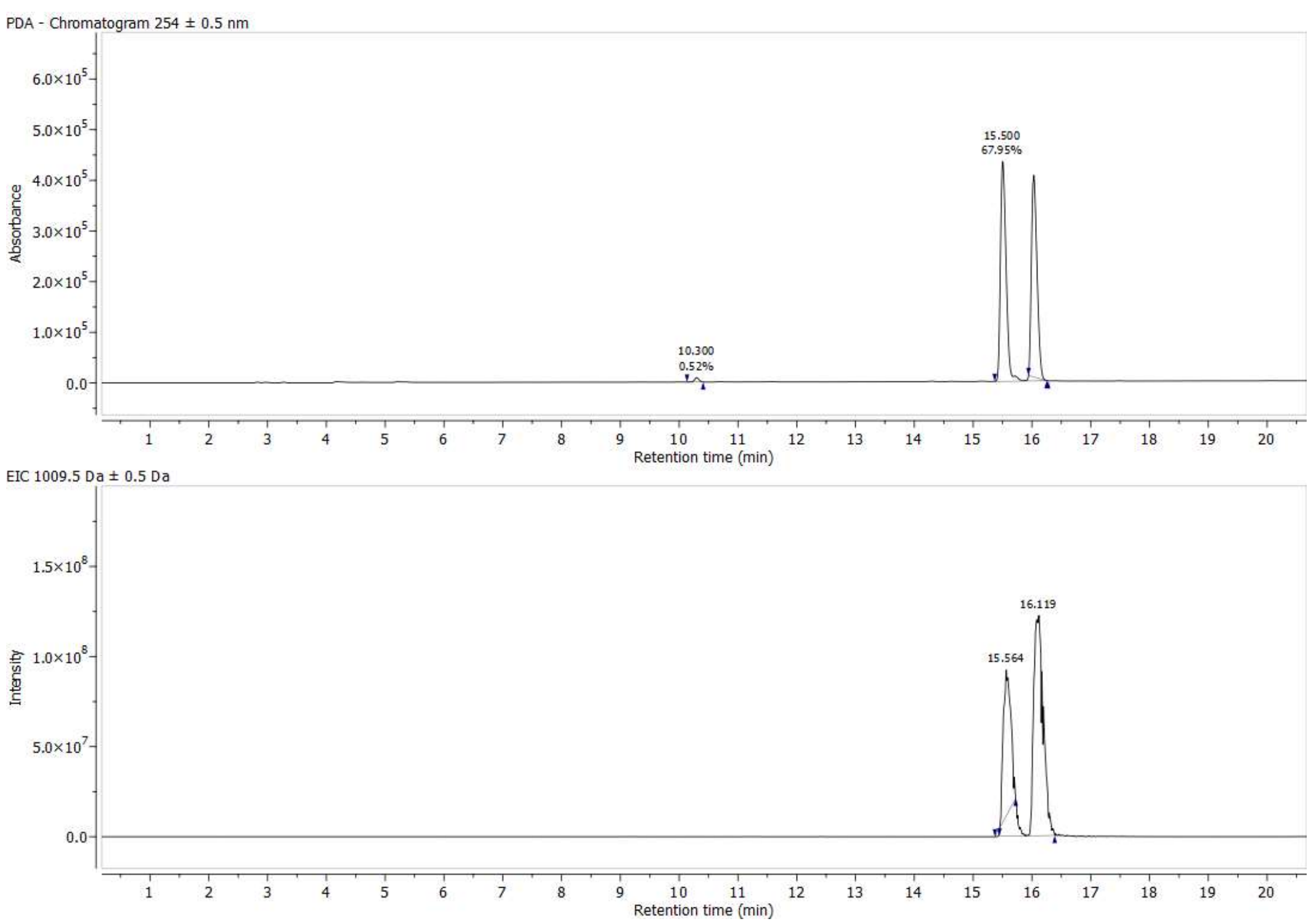

Monomer **7**

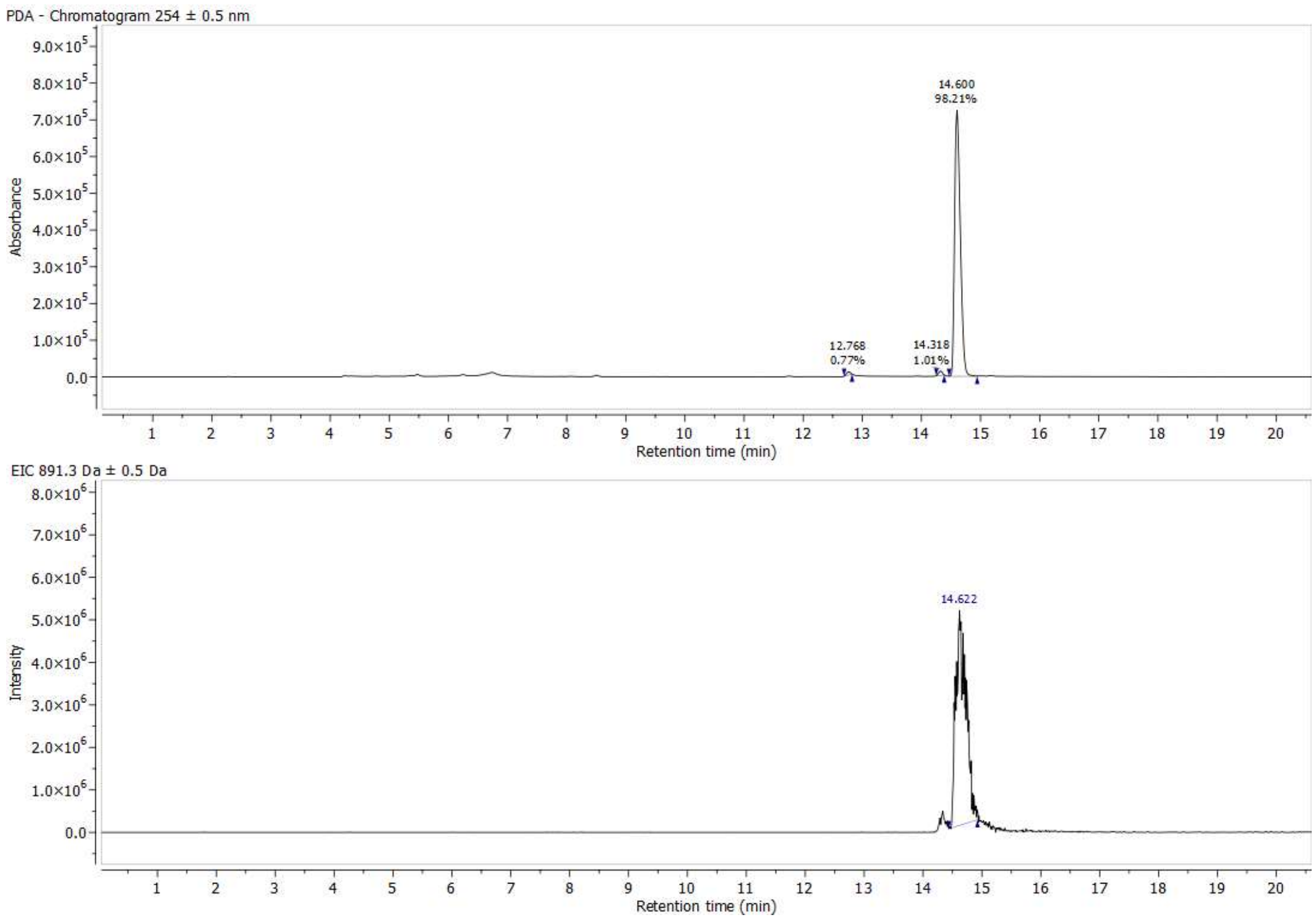

Monomer **8**

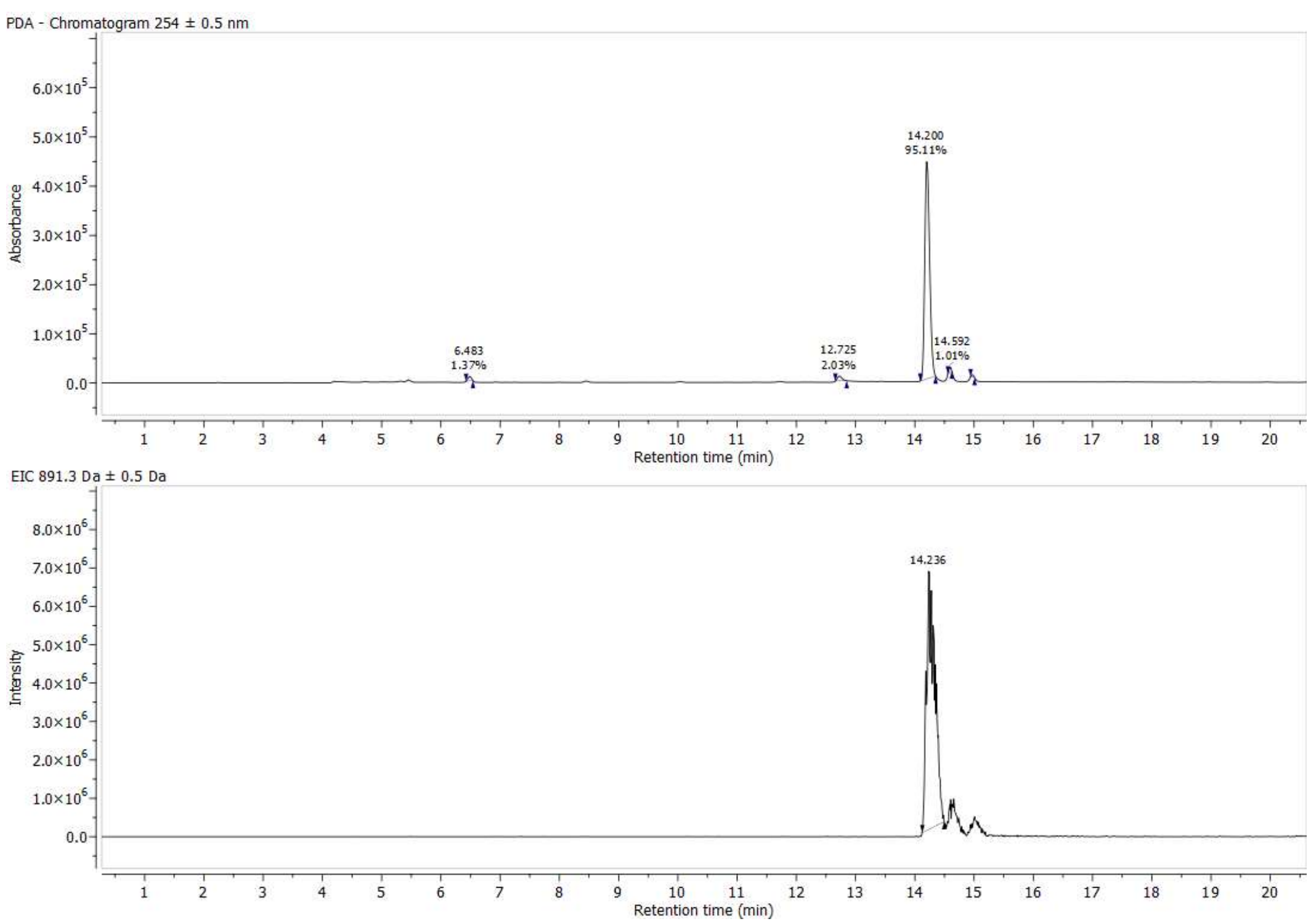

Monomer **9**

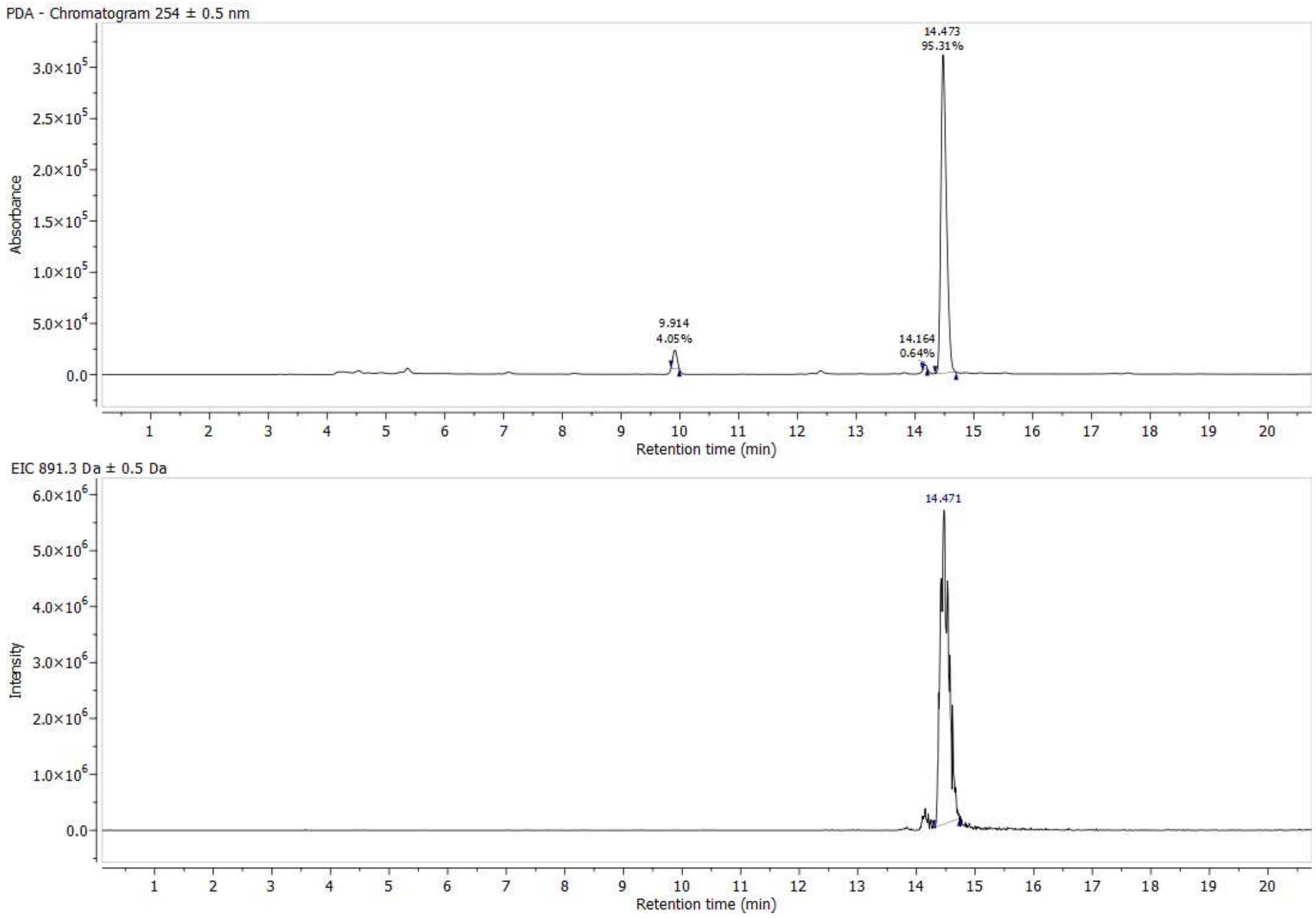

Monomer **10**

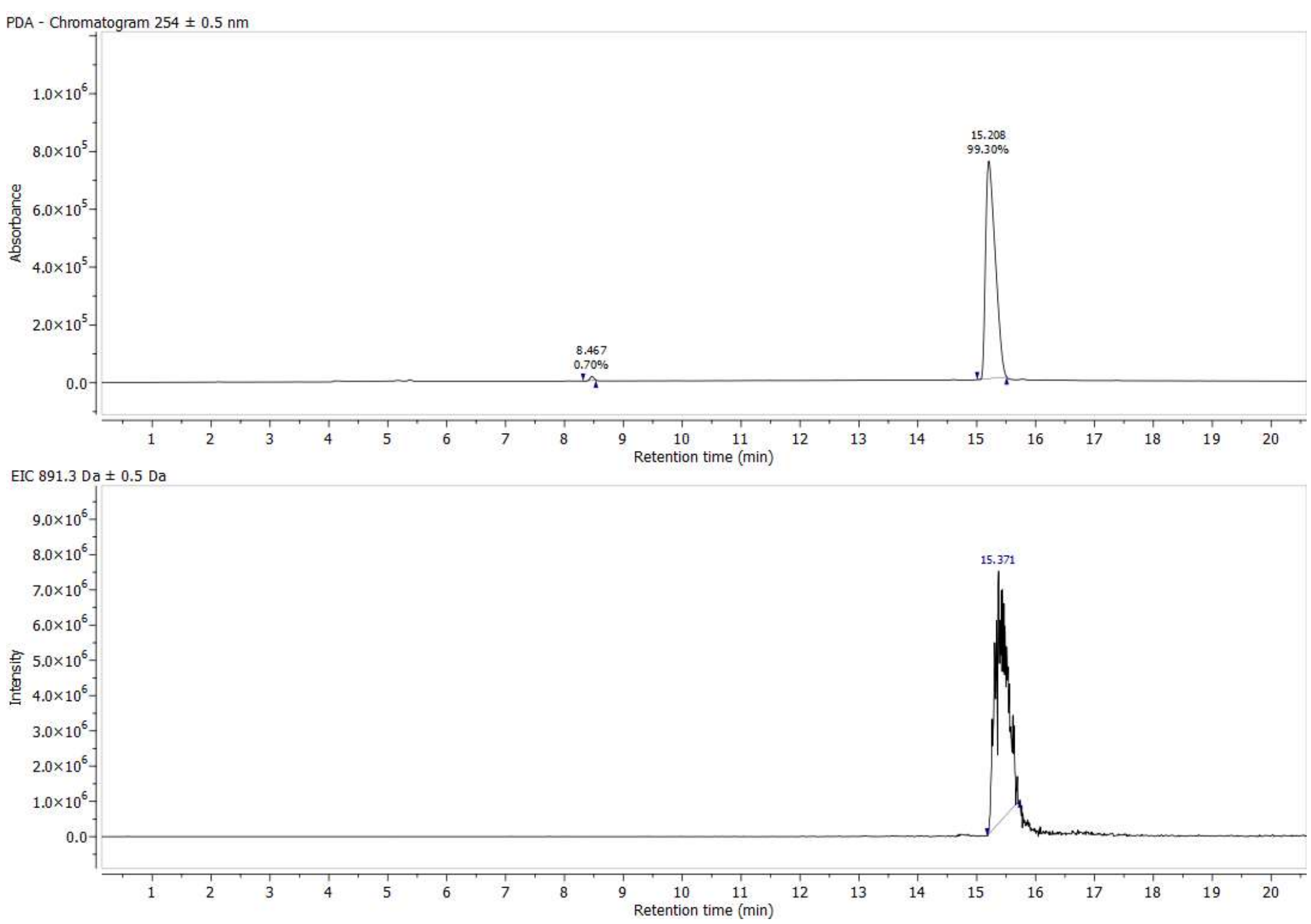

Monomer **11**

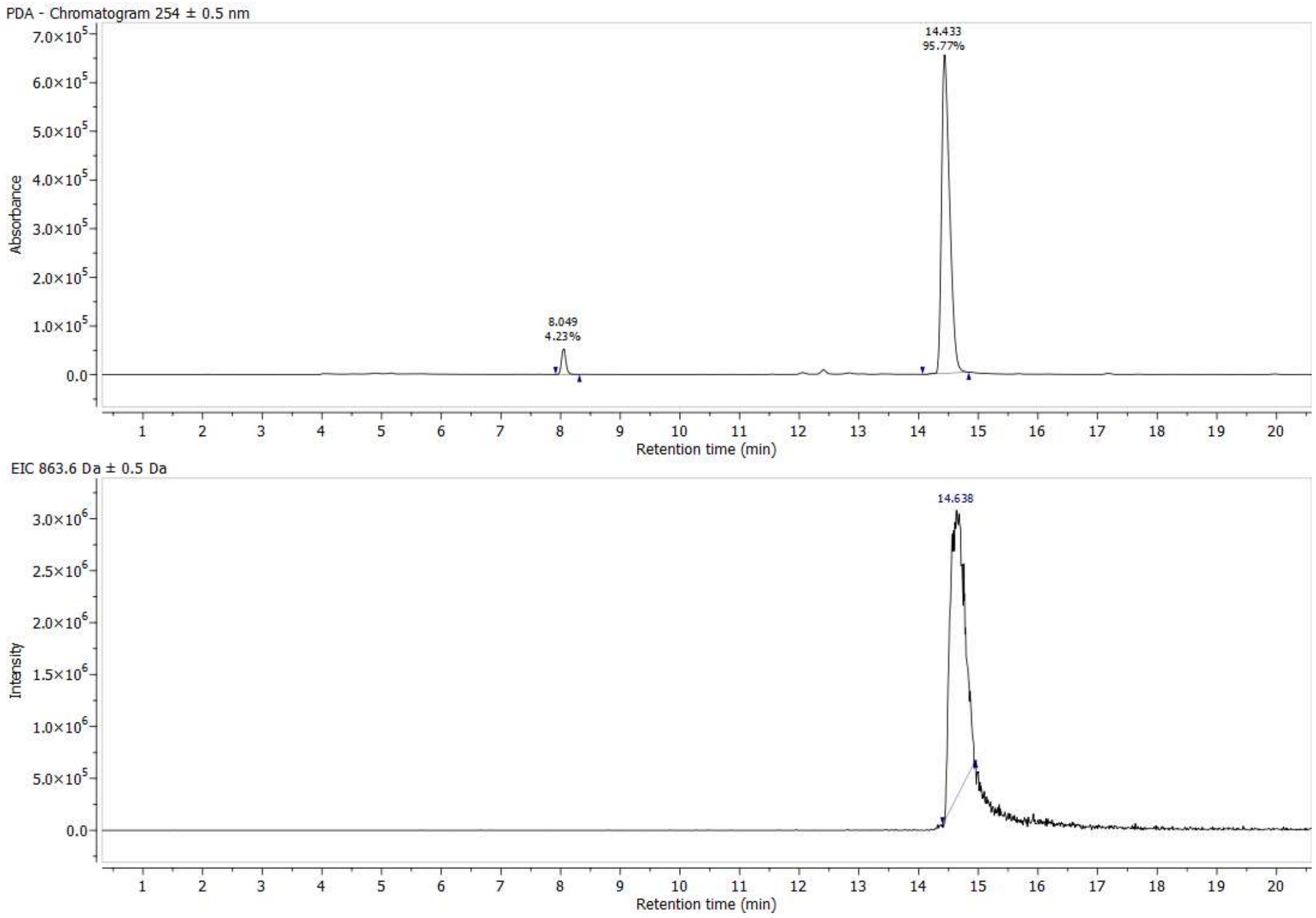

Monomer **12**

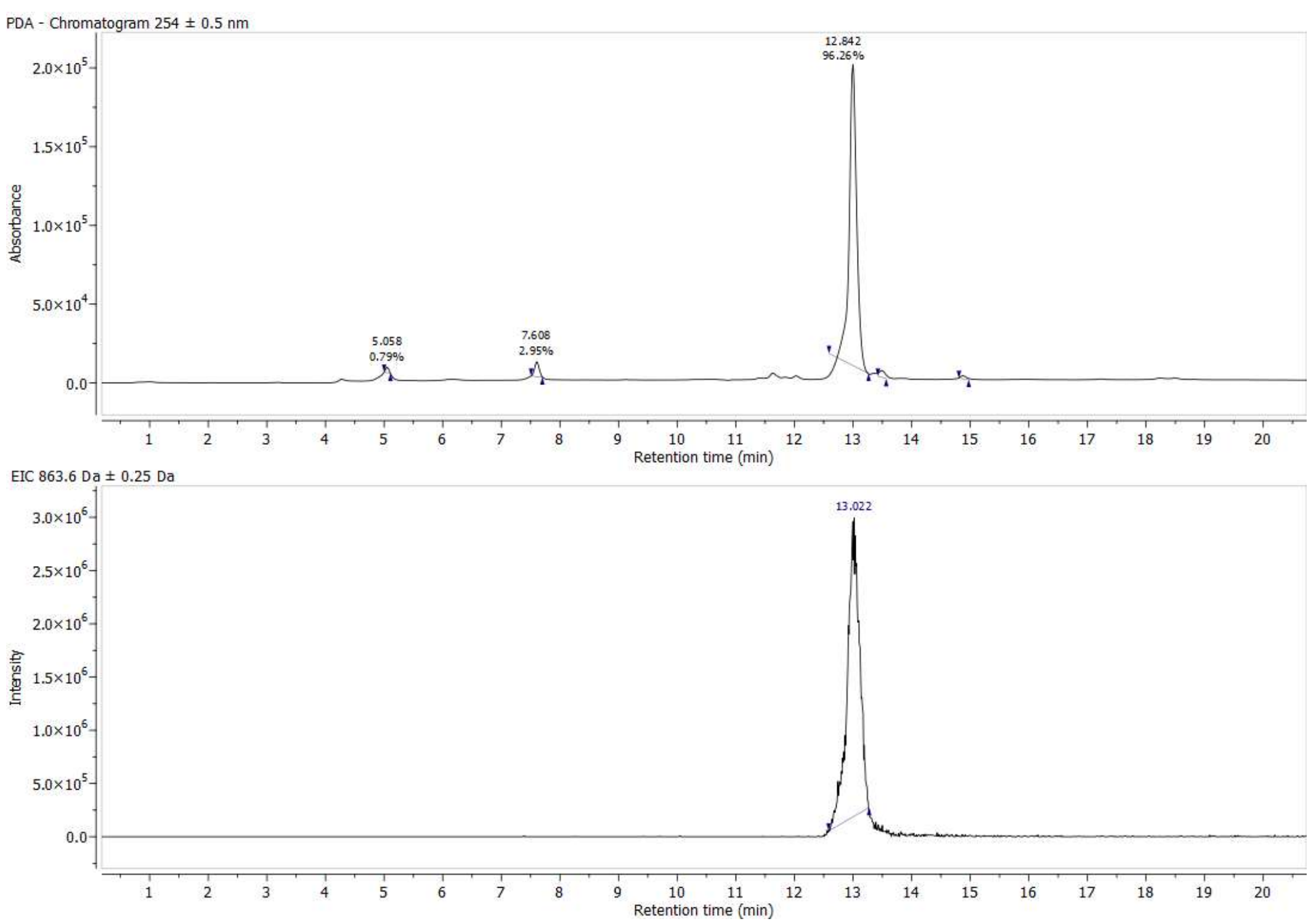

Monomer **13**

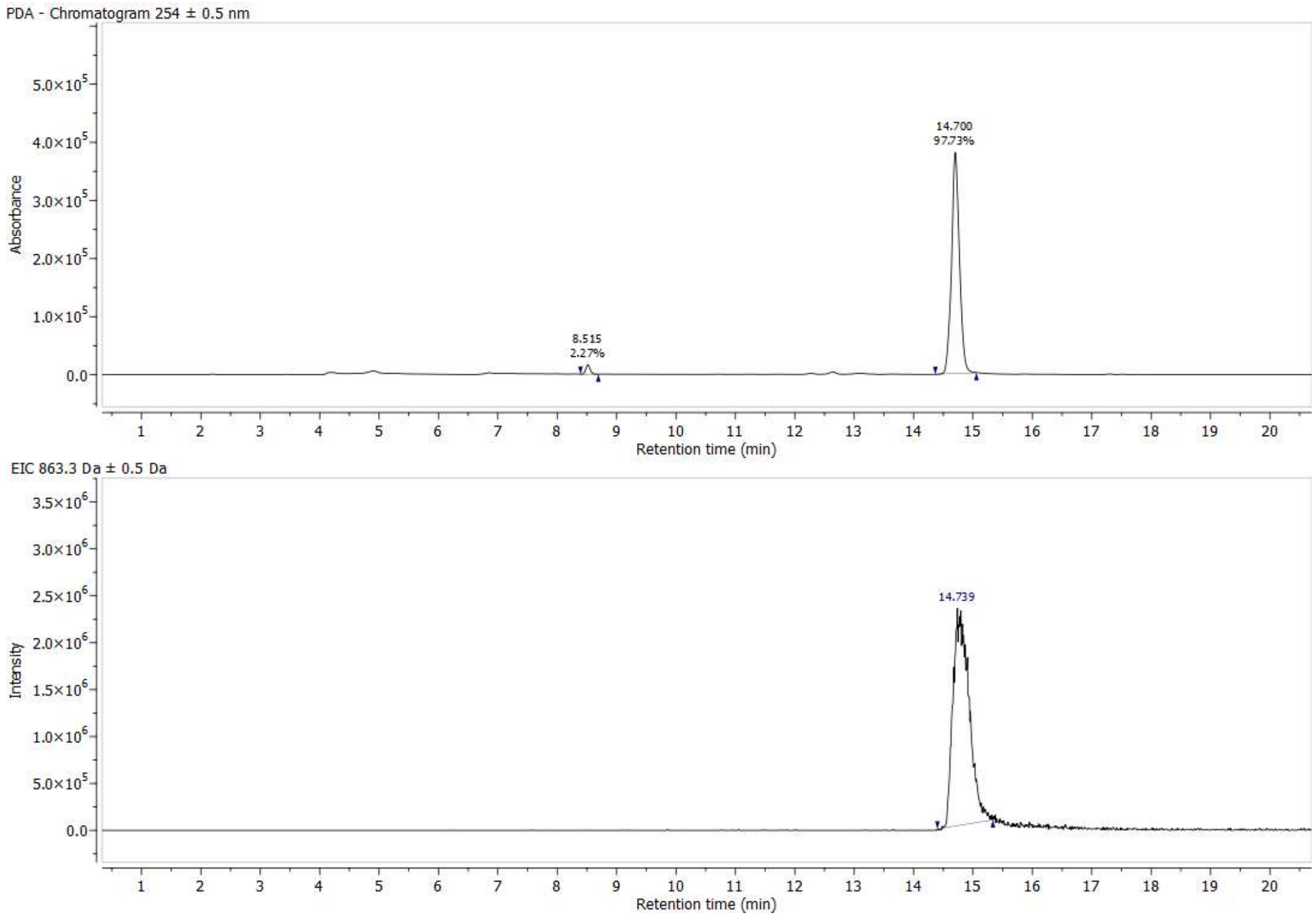

Monomer **14**

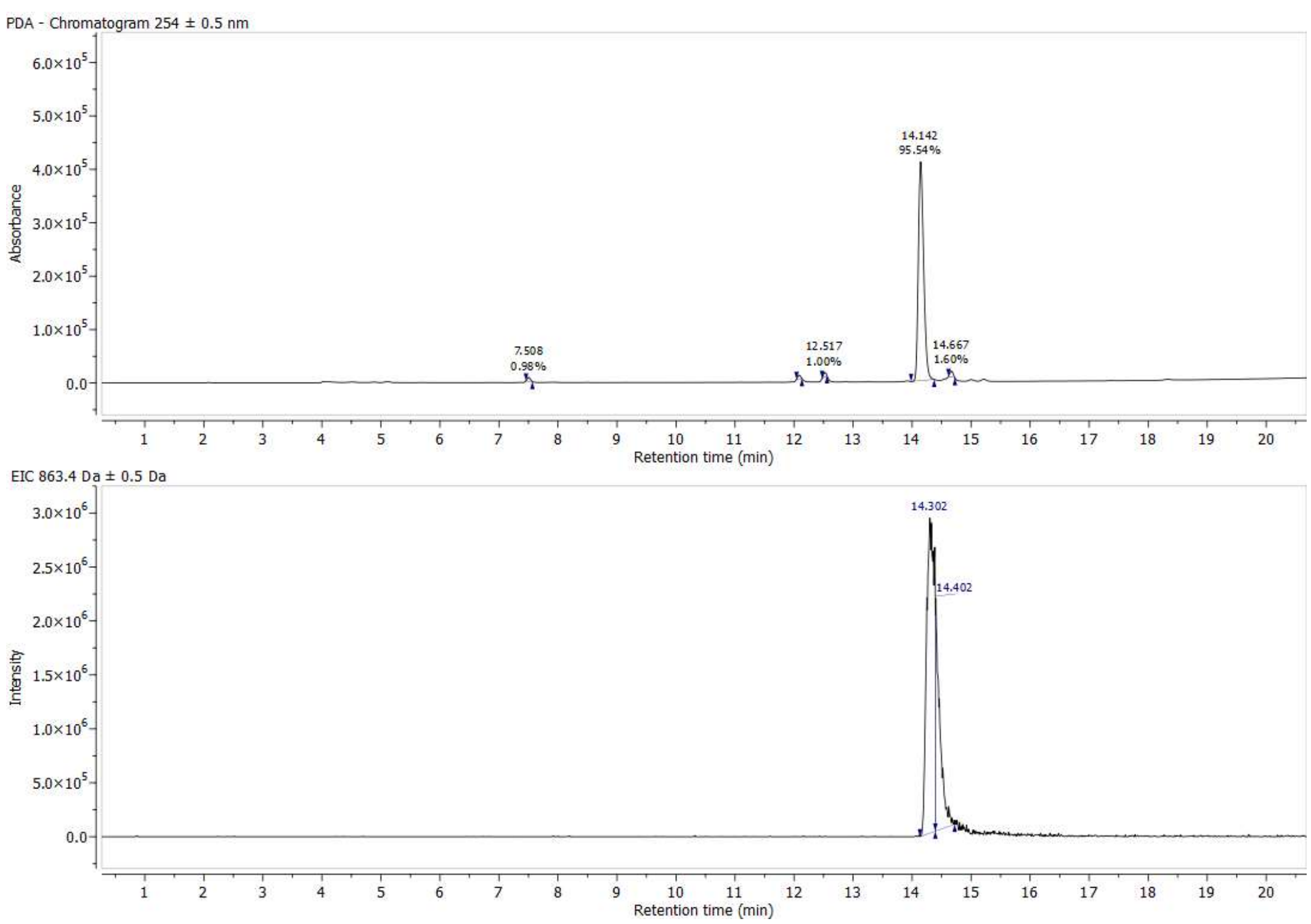

Monomer **15**

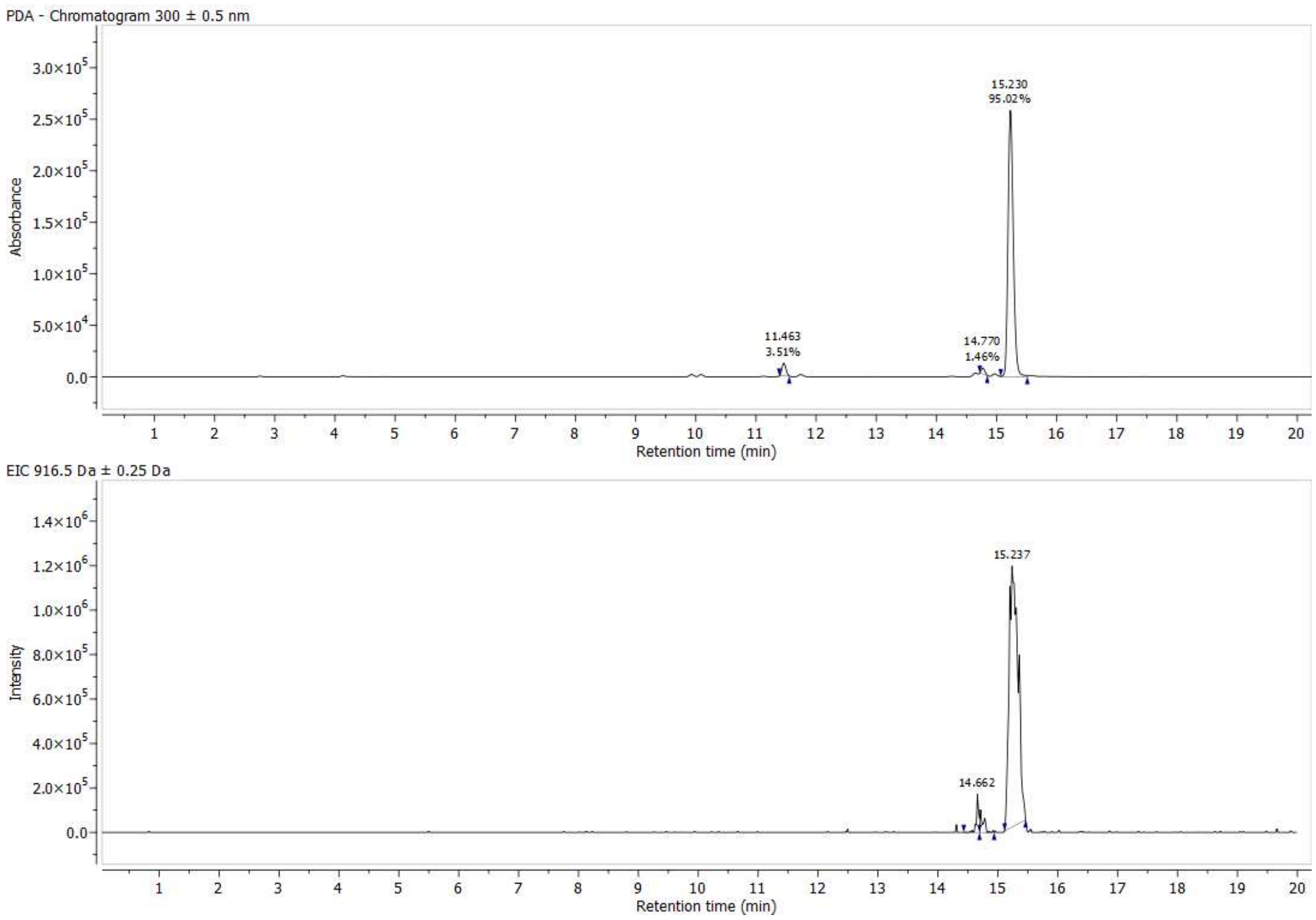

Monomer **16**

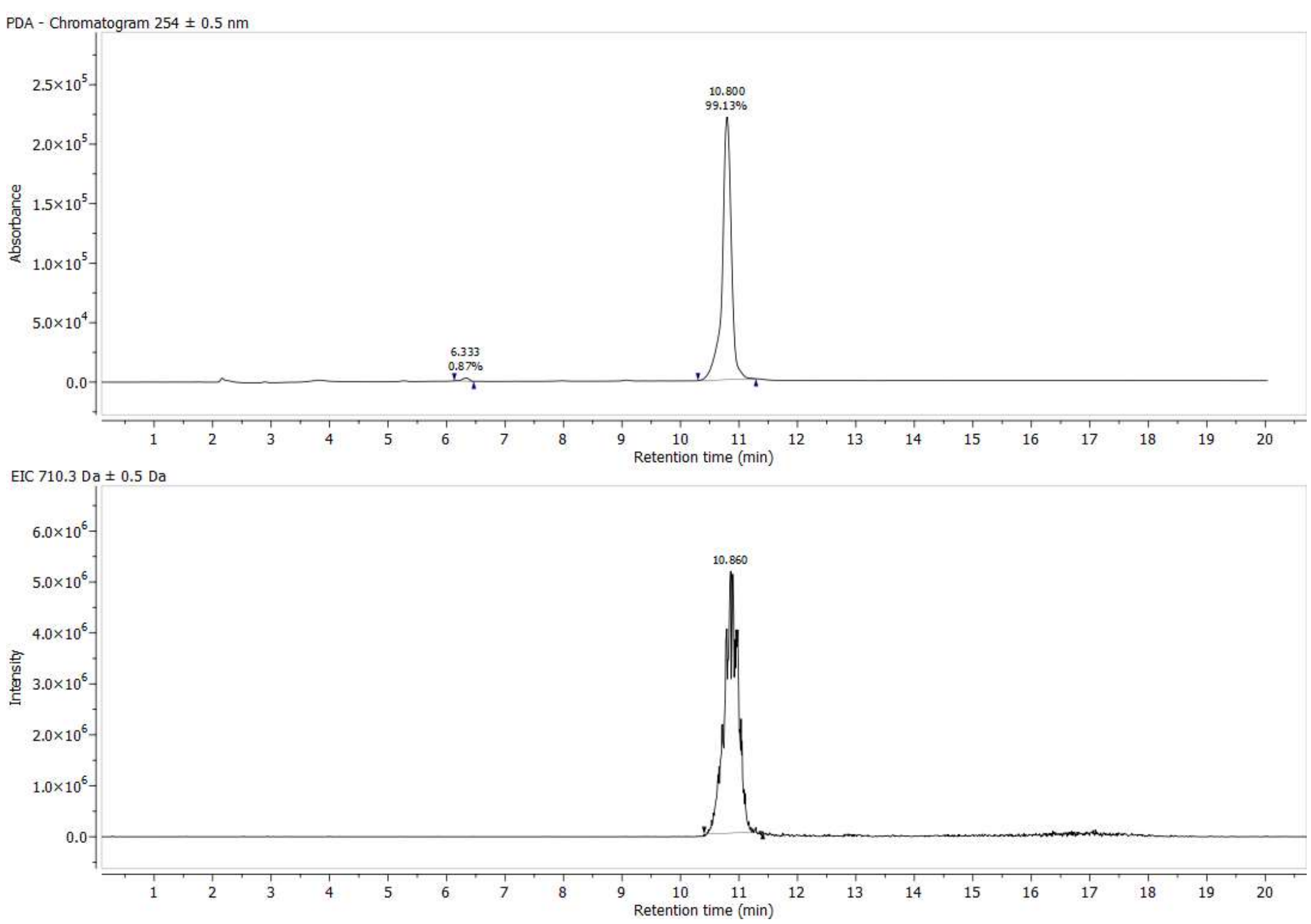

MALDI-TOF MS and purity data for prepared ODNs

Code	Internal code	$(M+H)^+$ calcd	$(M+H)^+$ found	Purity (%)	Gradient
**ODN1**	ON-695c	7746.7	7746.6	99	5%B-65%B/20min in A, 60 °C
**ODN2**	ON-678a	7746.7	7747.3	99	5%B-75%B/20min in A, 80 °C
**ODN3**	ON-742c	7805.7	7805.4	97	10%B-80%B/20min in A, 80 °C
**ODN4**	ON-742b	8053.7	8053.6	98	10%B-80%B/20min in A, 80 °C
**ODN5**	ON-742e	7805.7	7804.8	99	10%B-80%B/20min in A, 80 °C
**ODN6**	ON-742d	7867.7	7867.0	97	10%B-80%B/20min in A, 80 °C
**ODN7**	ON-745a	7805.7	7805.8	99	5%B-75%B/20min in A, 80 °C
**ODN8**	ON-745b	7805.7	7805.6	98	5%B-75%B/20min in A, 80 °C
**ODN9**	ON-745c	7805.7	7805.9	98	5%B-75%B/20min in A, 80 °C
**ODN10**	ON-745d	7805.7	7805.8	95	5%B-75%B/20min in A, 80 °C
**ODN11**	ON-695a	7689.7	7689.4	98	5%B-60%B/20min in A, 60 °C
**ODN12**	ON-728c	7689.7	7689.8	99	5%B-75%B/20min in A, 80 °C
**ODN13**	ON-728a	7689.7	7689.8	98	5%B-75%B/20min in A, 80 °C
**ODN14**	ON-728b	7689.7	7689.6	99	5%B-75%B/20min in A, 80 °C
**ODN15**	ON-695f	7753.7	7753.8	99	5%B-75%B/20min in A, 80 °C
**ODN16**	ON-691b	7755.2	7755.2	96	5%B-75%B/20min in A, 80 °C
**ODN-A**	ON-761A	1808.6	1808.9	96	5%B-35%B/20min in A, 80 °C
**ODN-B**	ON-761B	1821.3	1821.0	99	5%B-15%B/20min in A, 80 °C
**ODN-C**	ON-761C	3194.2	3194.6	99	5%B-15%B/20min in A, 80 °C

Table S5. MALDI-TOF MS and purity data for prepared ODNs IE analysis on a DNAPac PA100 column (4 × 250 mm; Dionex) at a flow rate of 1 mL/min using mobile phases: A, 25 mM TRIS/HCl and 10 mM $NaClO_4$ in MeCN-H_2O (20:80); B, 25 mM NaOAc and 0.5 M $NaClO_4$ in MeCN-H_2O (20:80), UV detection at 254 nm.

Purity chromatograms and MALDI-TOF MS of prepared oligonucleotides

ODN1

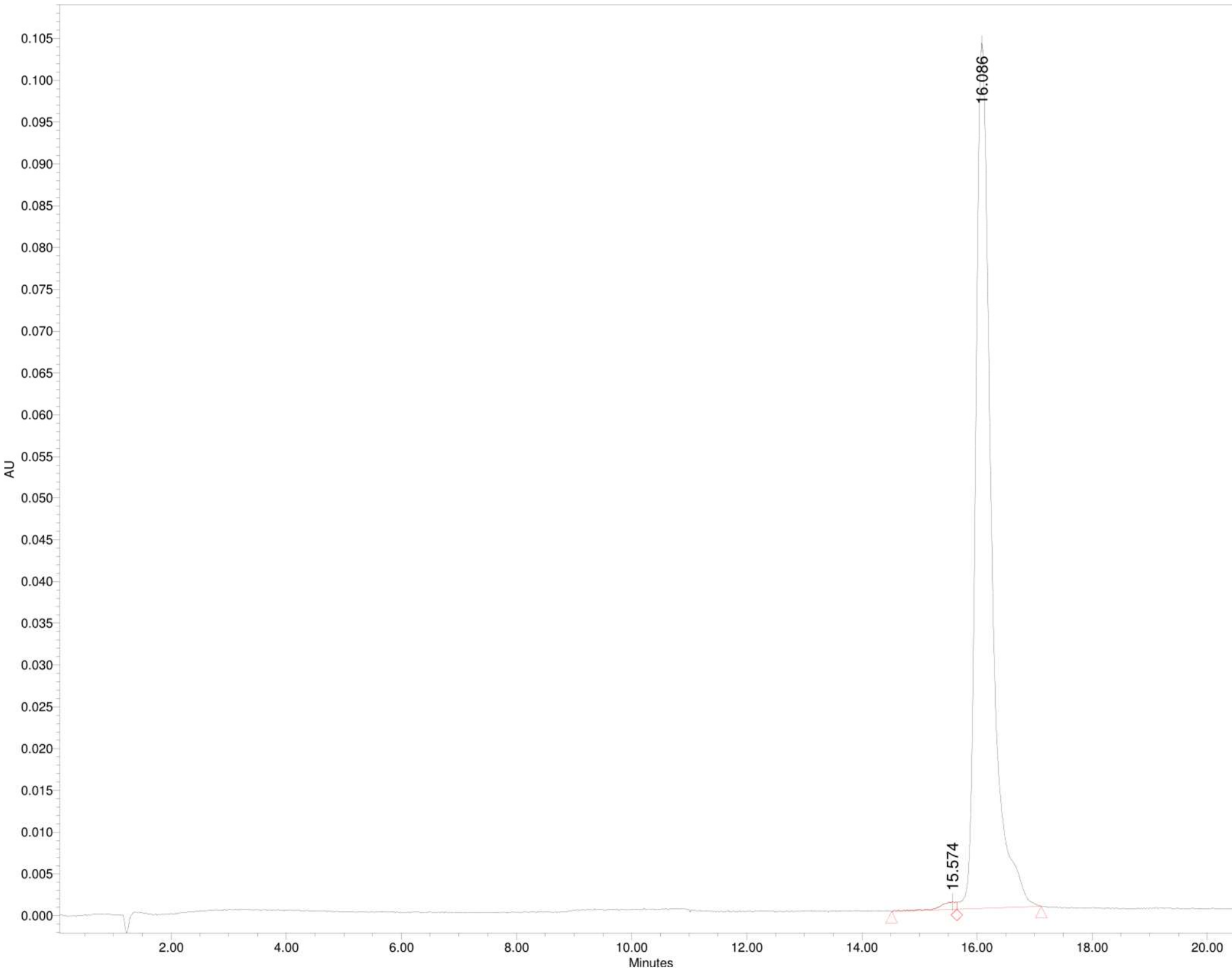

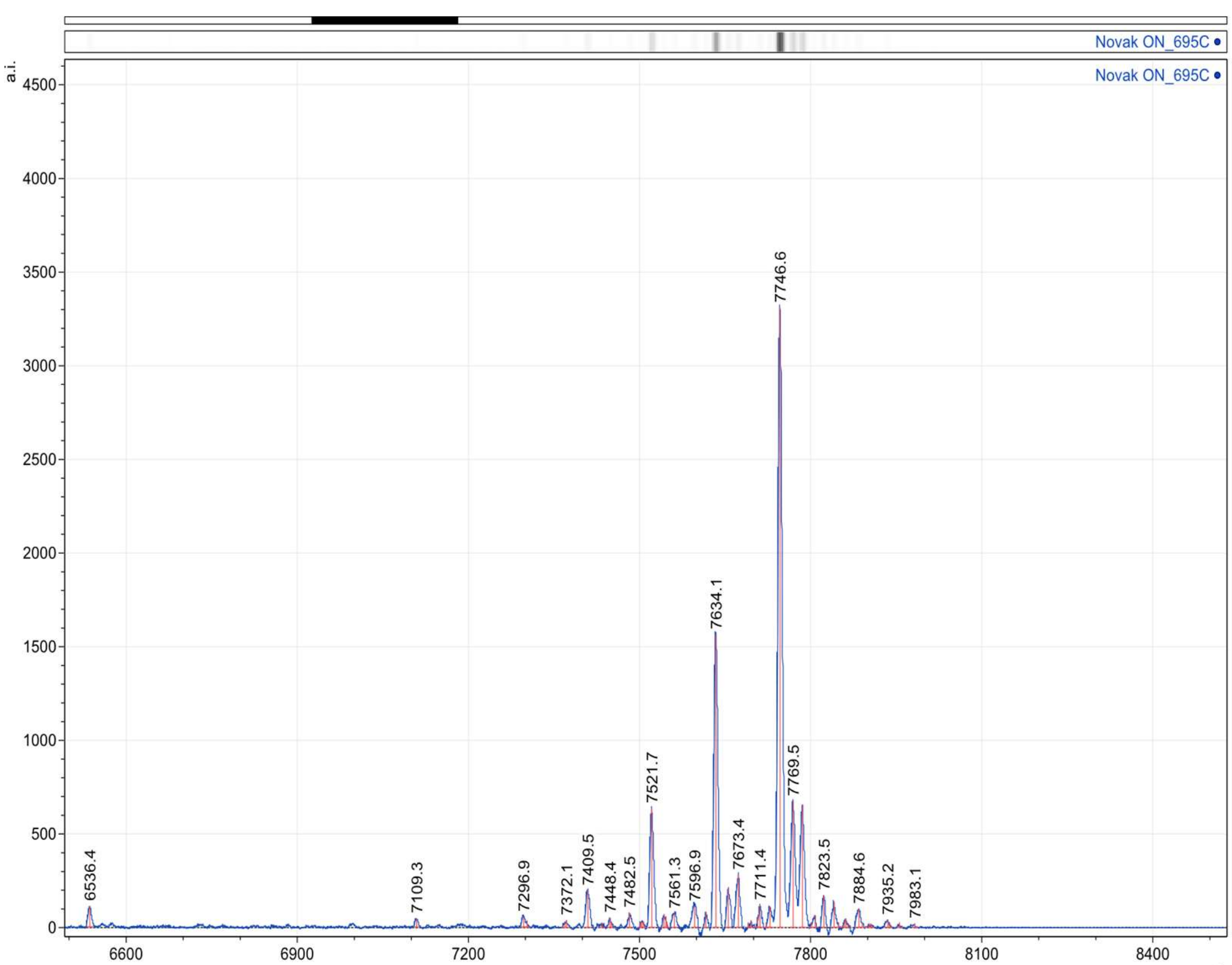

ODN2

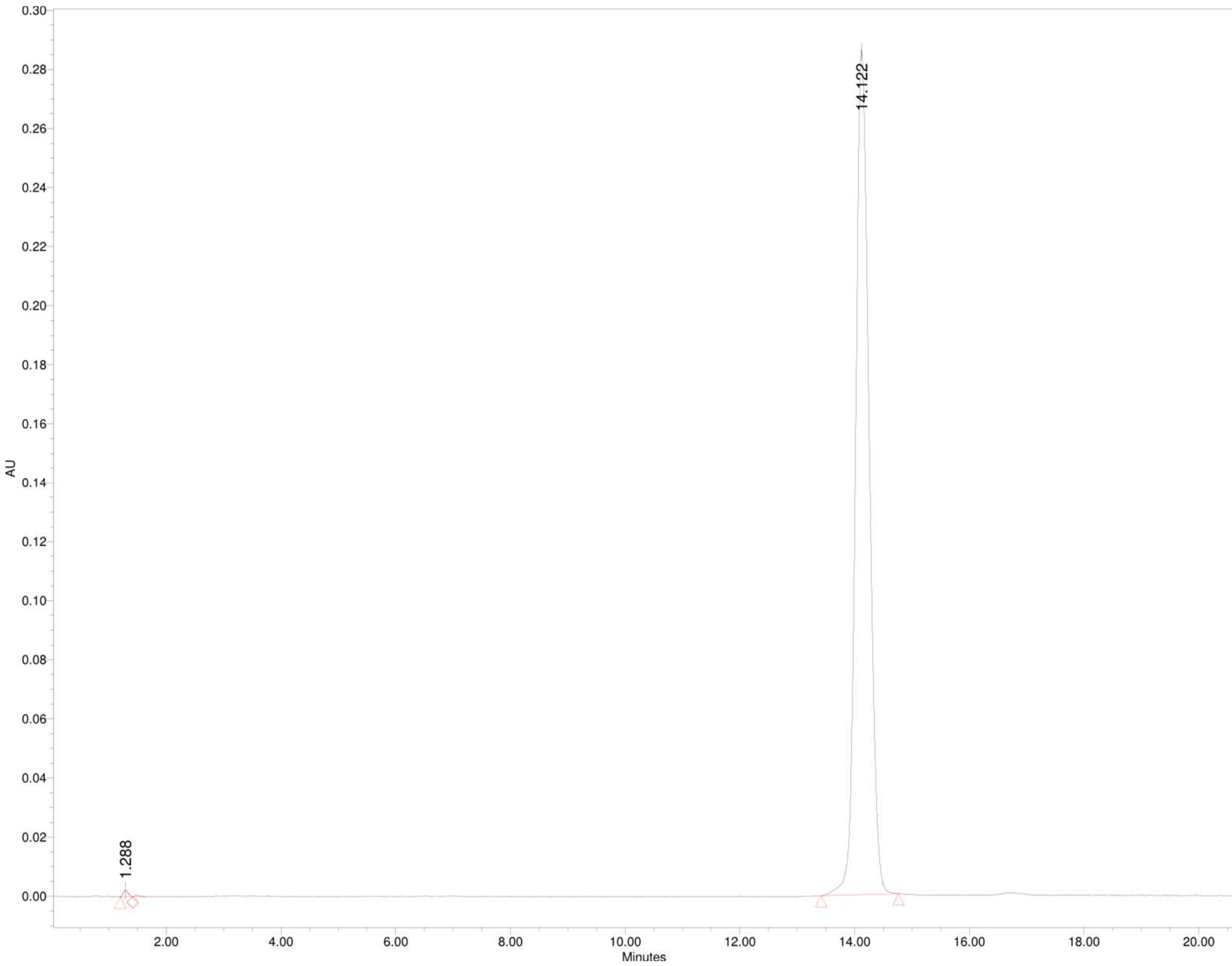

AU
Minutes
14.122
1.288
0.30
0.28
0.26
0.24
0.22
0.20
0.18
0.16
0.14
0.12
0.10
0.08
0.06
0.04
0.02
0.00
2.00
4.00
6.00
8.00
10.00
12.00
14.00
16.00
18.00
20.00

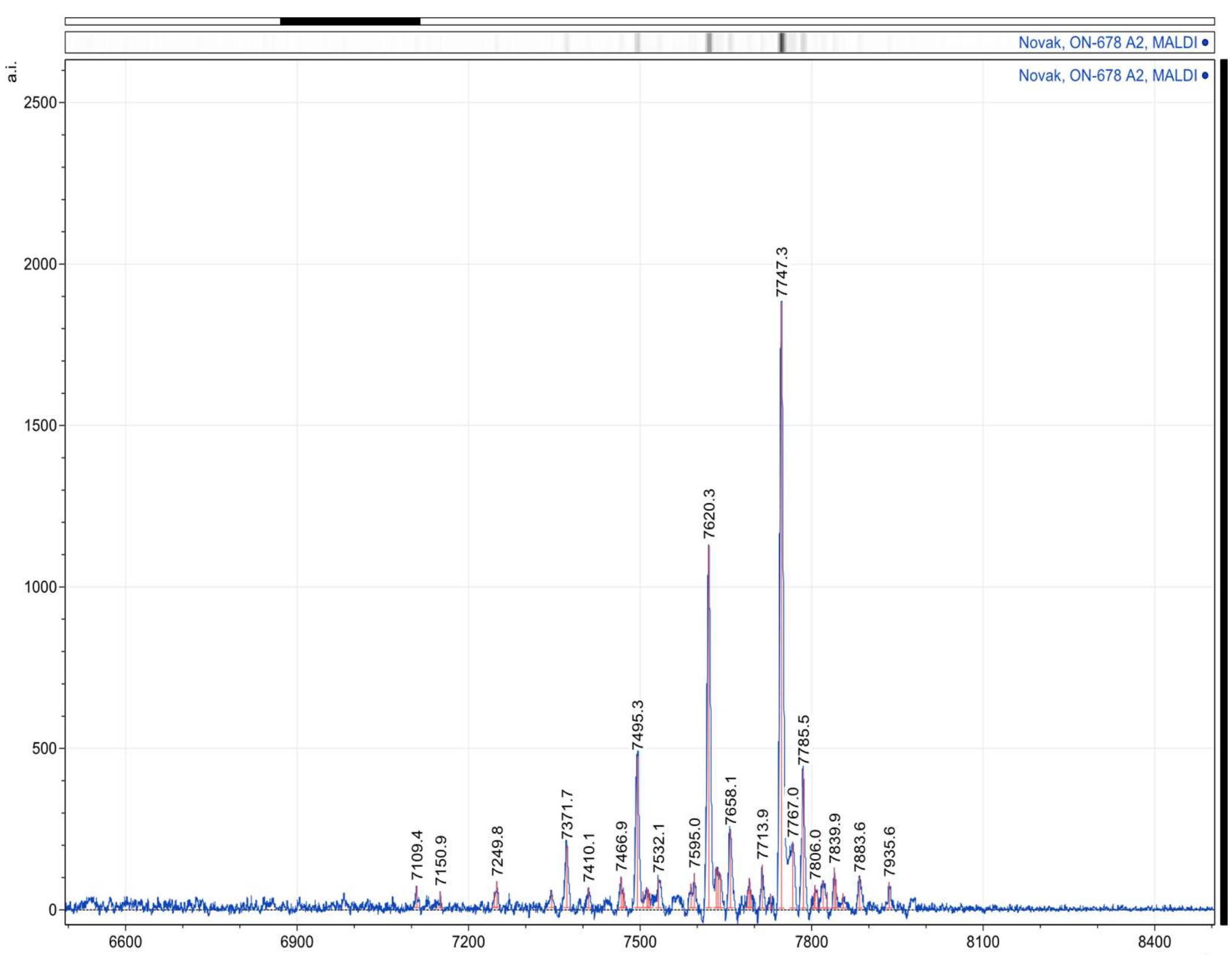

Novak, ON-678 A2, MALDI
Novak, ON-678 A2, MALDI
a.i.
m/z
7747.3
7620.3
7495.3
7785.5
7371.7
7658.1
7767.0
7109.4
7150.9
7249.8
7410.1
7466.9
7532.1
7595.0
7713.9
7806.0
7839.9
7883.6
7935.6
2500
2000
1500
1000
500
0
6600
6900
7200
7500
7800
8100
8400

ODN3

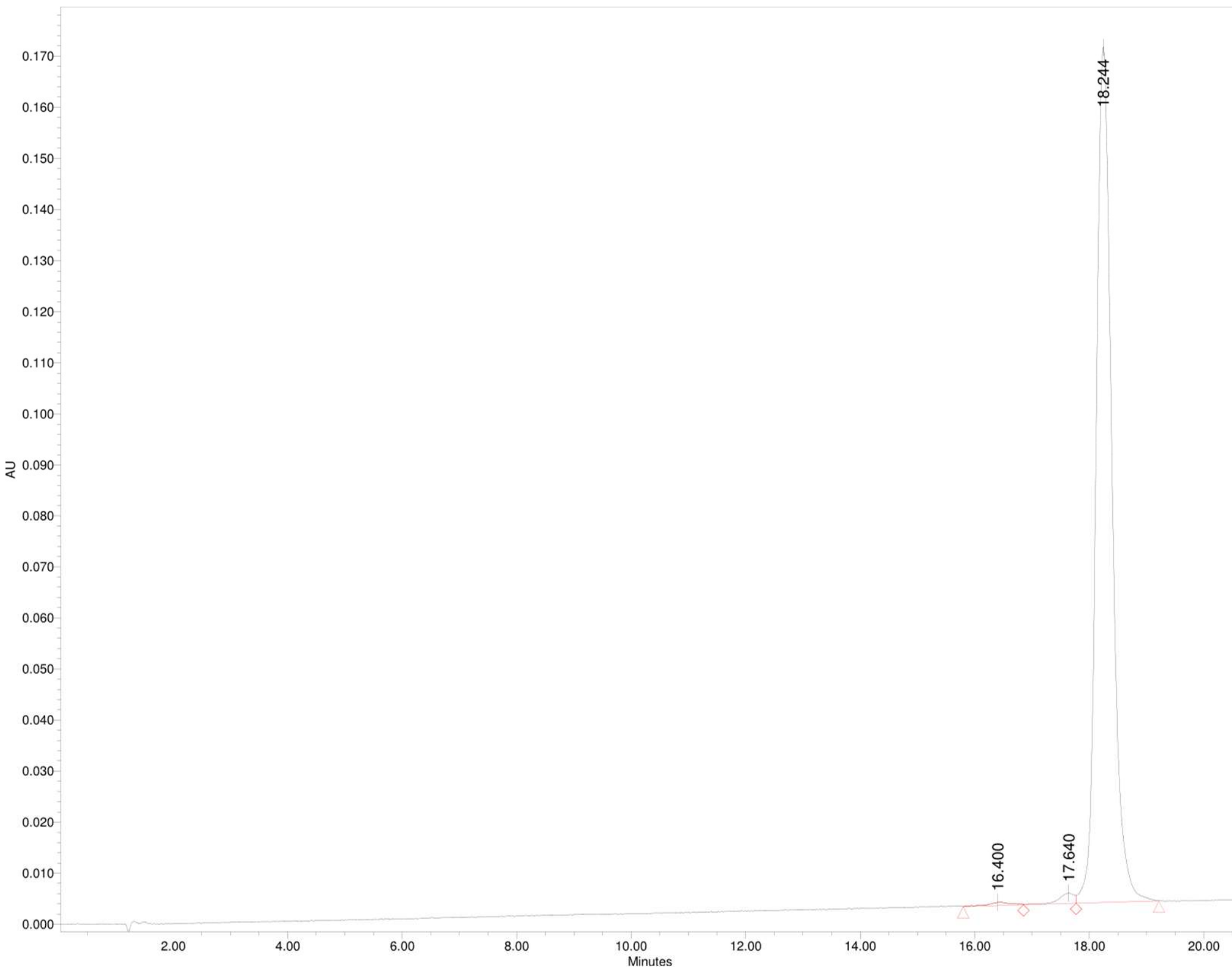

18.244
16.400
17.640
AU
Minutes

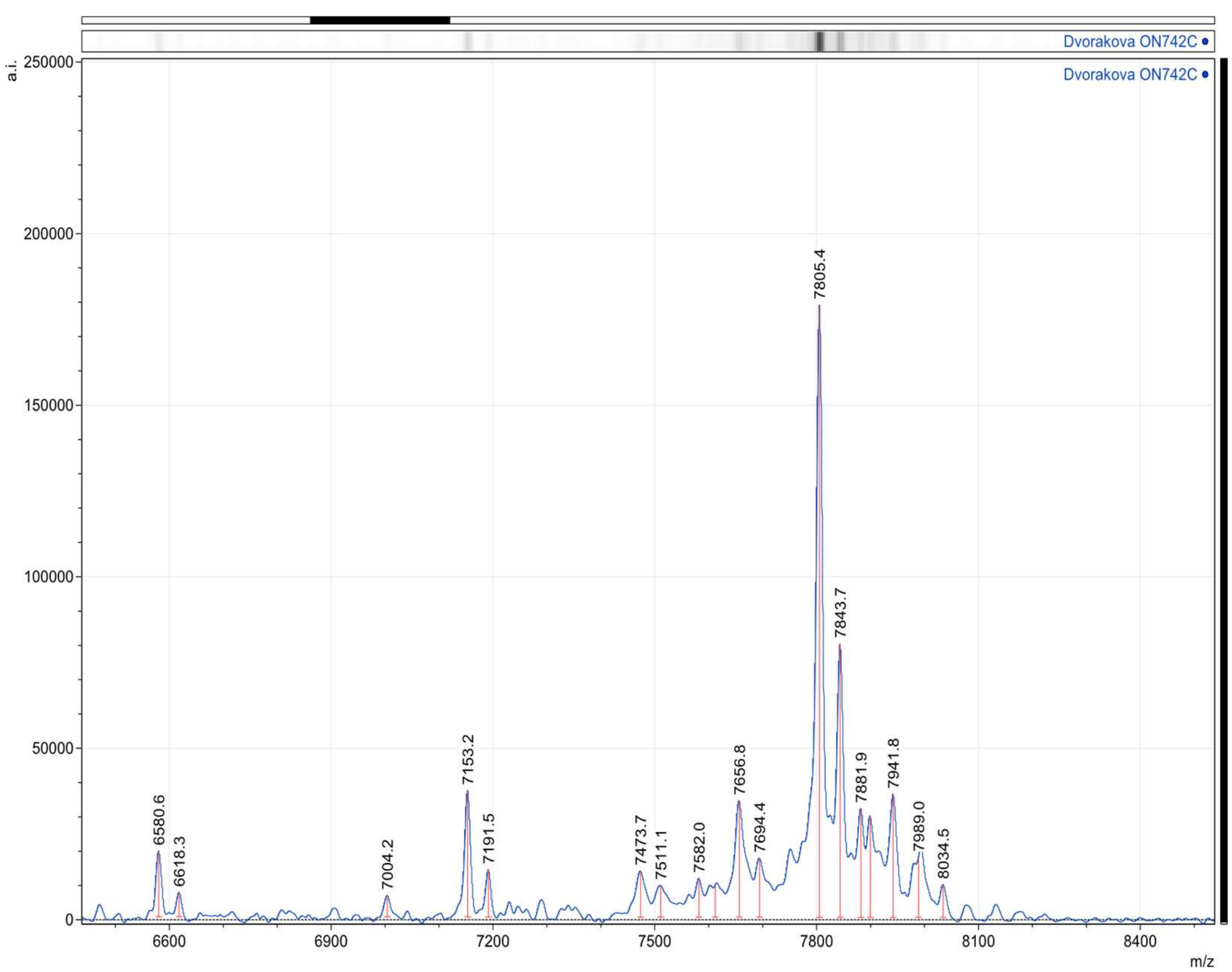

Dvorakova ON742C
a.i.
7805.4
7843.7
7881.9
7941.8
7989.0
8034.5
7153.2
7191.5
7656.8
7694.4
7582.0
7511.1
7473.7
7004.2
6580.6
6618.3
m/z

ODN4

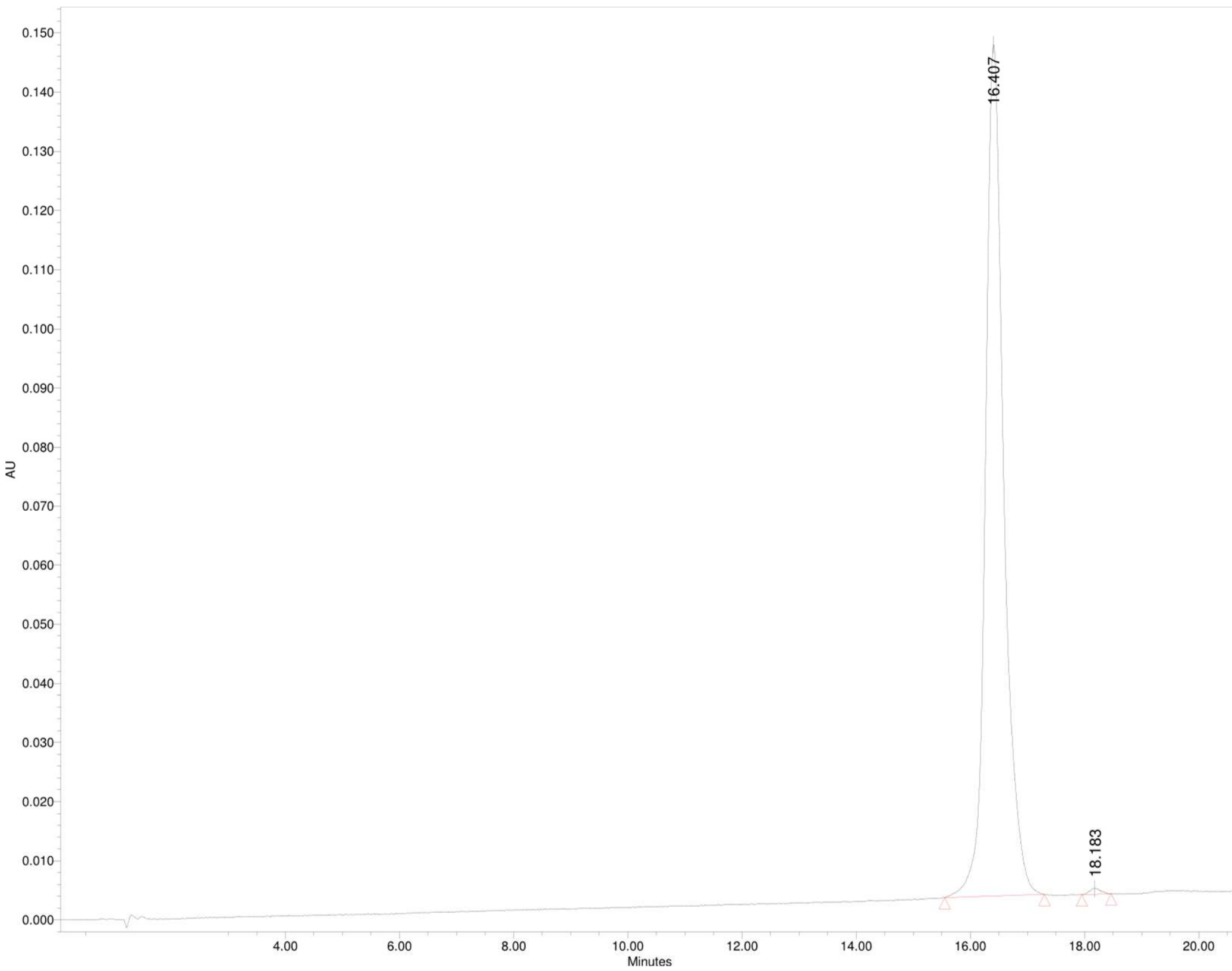

0.150
0.140
0.130
0.120
0.110
0.100
0.090
0.080
0.070
0.060
0.050
0.040
0.030
0.020
0.010
0.000
AU
16.407
18.183
4.00
6.00
8.00
10.00
12.00
14.00
16.00
18.00
20.00
Minutes

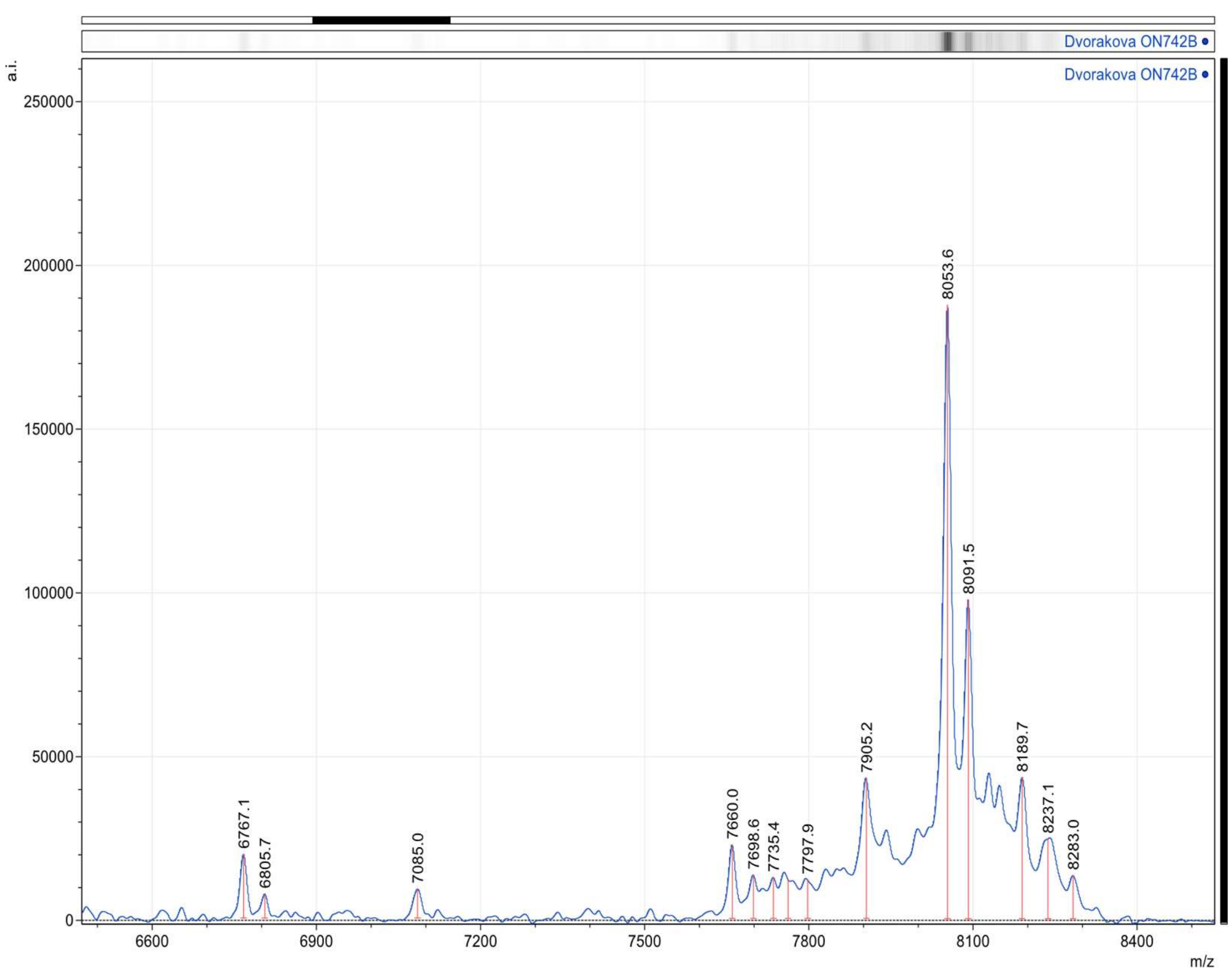

Dvorakova ON742B
Dvorakova ON742B
a.i.
250000
200000
150000
100000
50000
0
8053.6
8091.5
7905.2
8189.7
8237.1
8283.0
7660.0
7698.6
7735.4
7797.9
6767.1
6805.7
7085.0
6600
6900
7200
7500
7800
8100
8400
m/z

ODN5

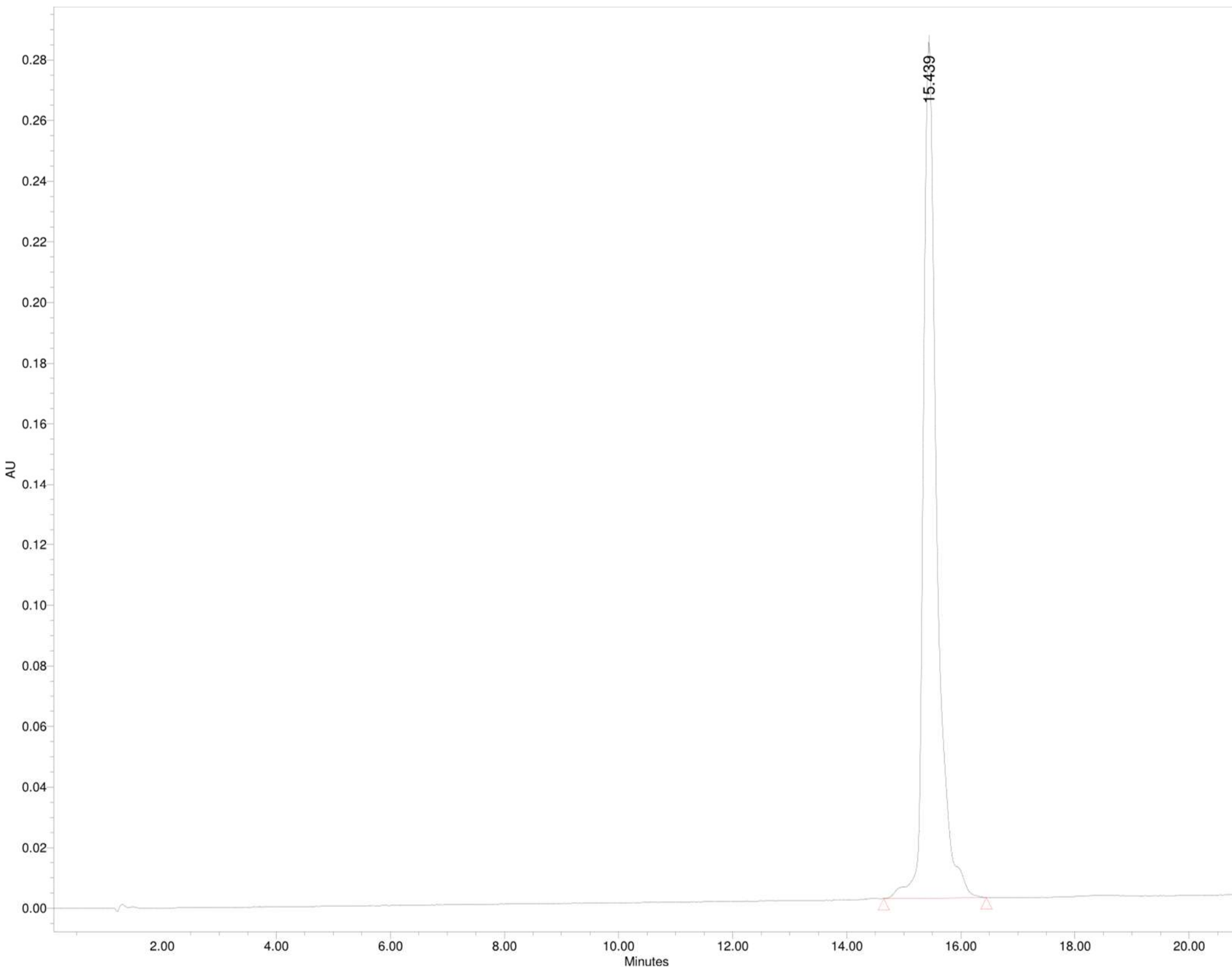

15.439
AU
0.28
0.26
0.24
0.22
0.20
0.18
0.16
0.14
0.12
0.10
0.08
0.06
0.04
0.02
0.00
2.00
4.00
6.00
8.00
10.00
12.00
14.00
16.00
18.00
20.00
Minutes

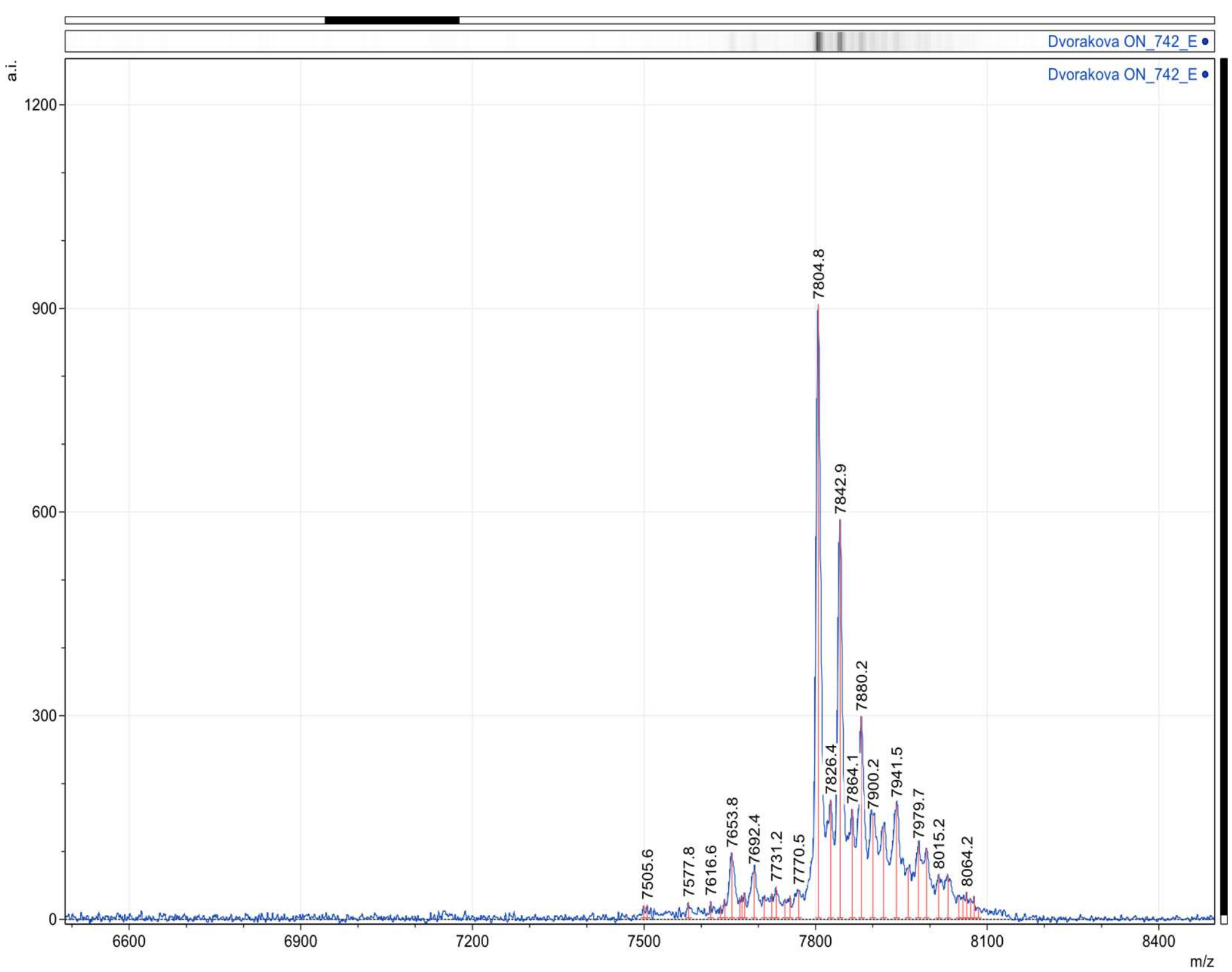

Dvorakova ON_742_E
a.i.
1200
900
600
300
0
7804.8
7842.9
7880.2
7826.4
7864.1
7900.2
7941.5
7979.7
8015.2
8064.2
7505.6
7577.8
7616.6
7653.8
7692.4
7731.2
7770.5
6600
6900
7200
7500
7800
8100
8400
m/z

ODN6

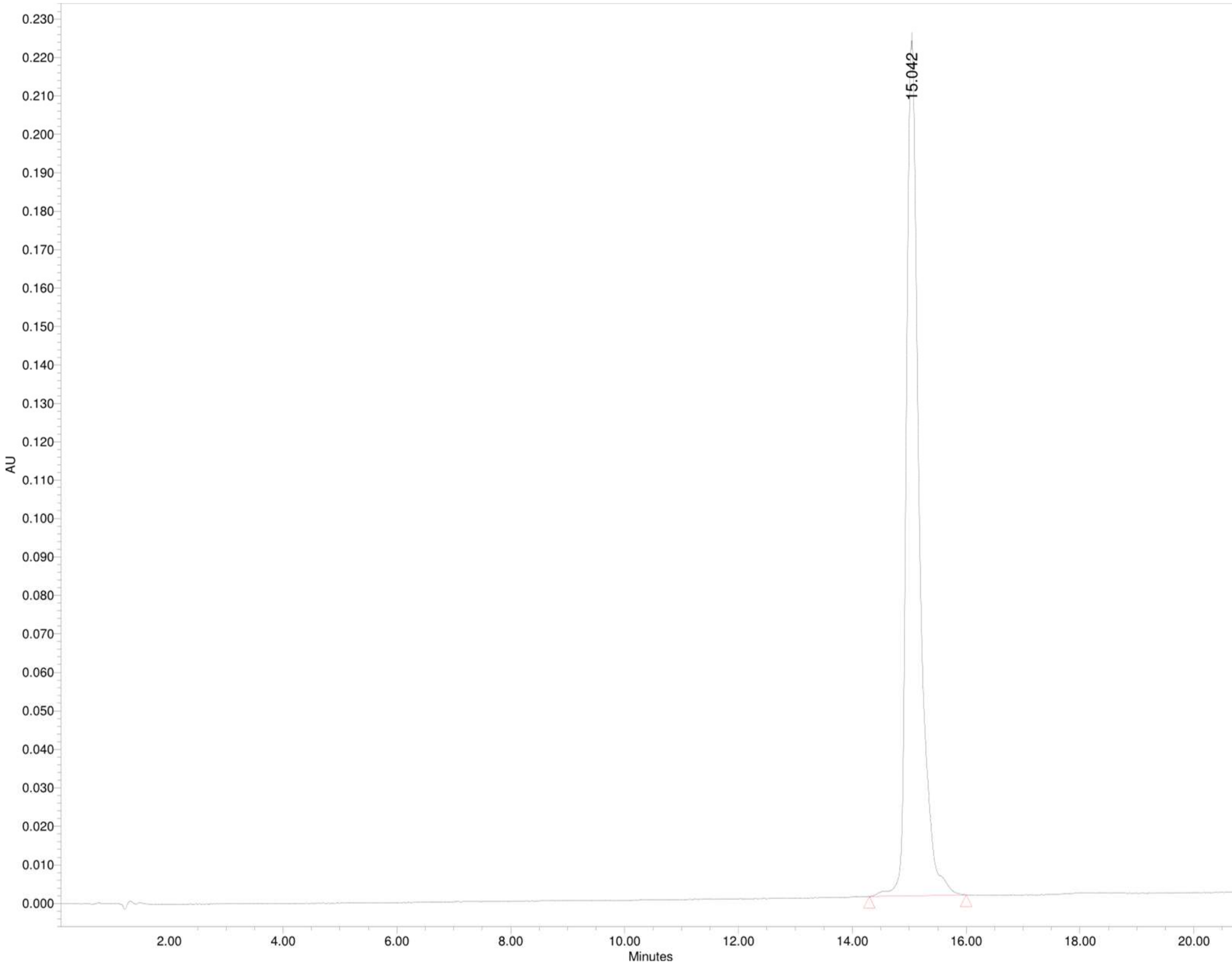

15.042
AU
Minutes
0.230
0.220
0.210
0.200
0.190
0.180
0.170
0.160
0.150
0.140
0.130
0.120
0.110
0.100
0.090
0.080
0.070
0.060
0.050
0.040
0.030
0.020
0.010
0.000
2.00
4.00
6.00
8.00
10.00
12.00
14.00
16.00
18.00
20.00

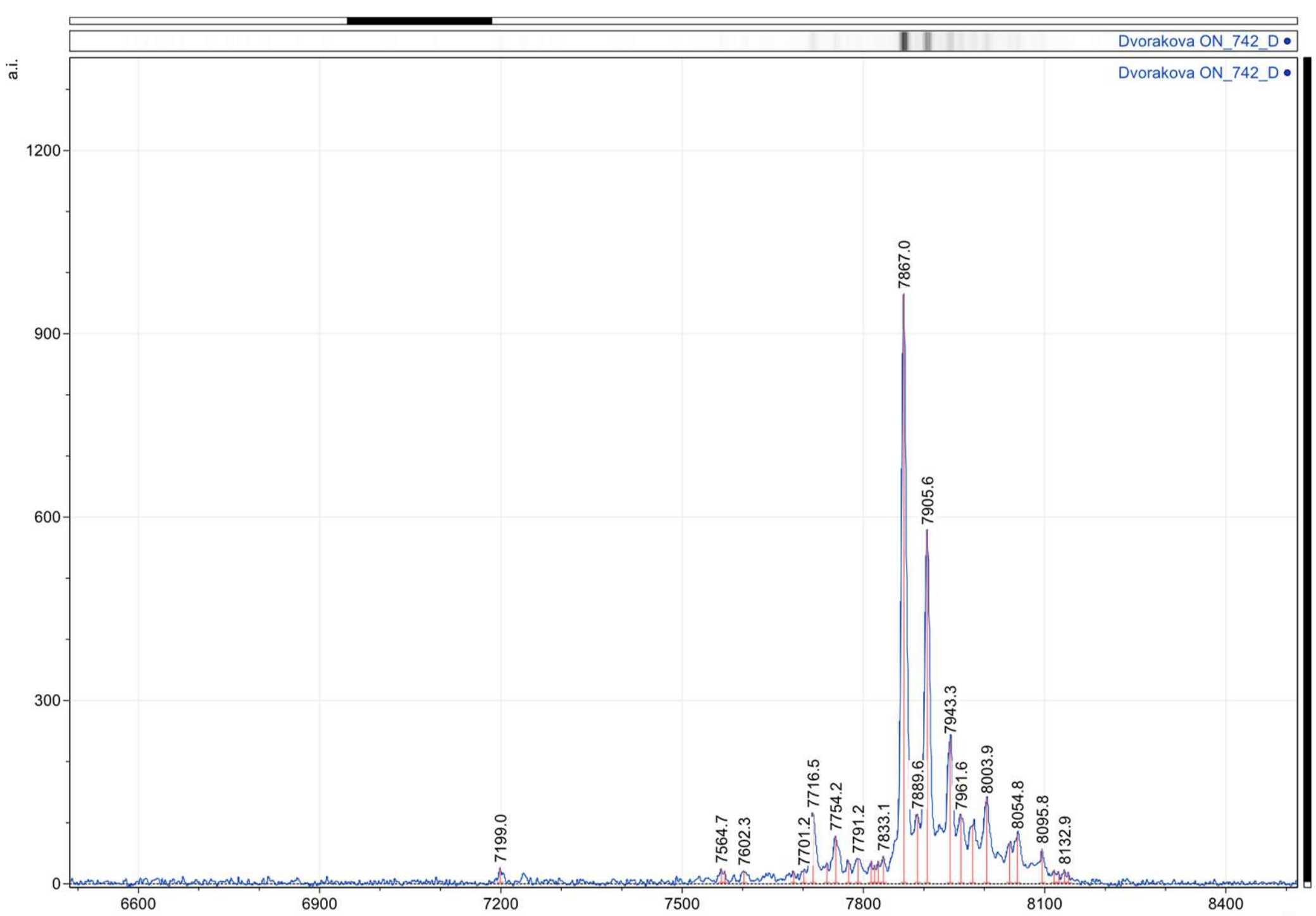

Dvorakova ON_742_D
Dvorakova ON_742_D
a.i.
1200
900
600
300
0
7867.0
7905.6
7943.3
7889.6
7961.6
8003.9
8054.8
8095.8
8132.9
7716.5
7754.2
7701.2
7791.2
7833.1
7199.0
7564.7
7602.3
6600
6900
7200
7500
7800
8100
8400
m/z

ODN7

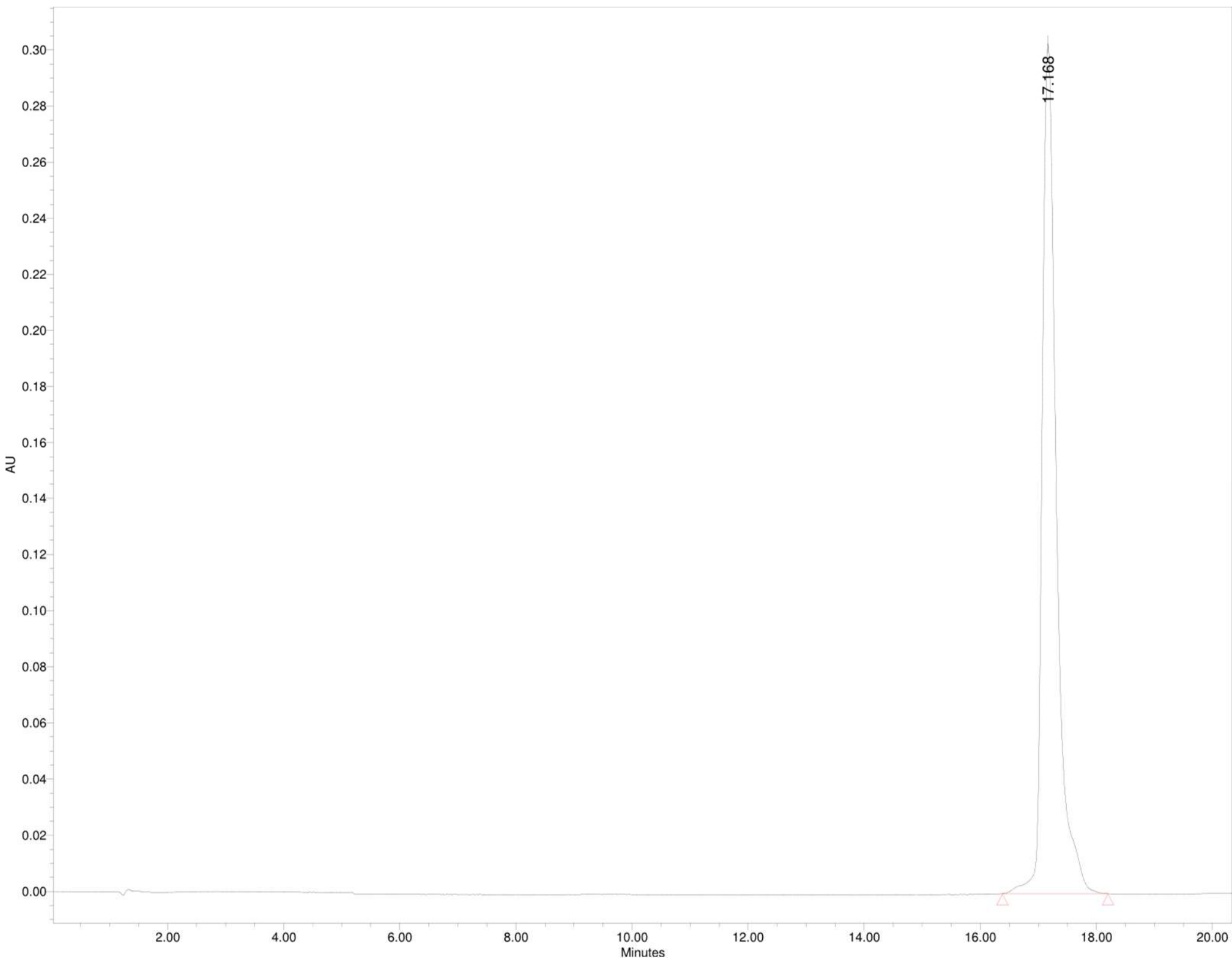

17.168
AU
Minutes

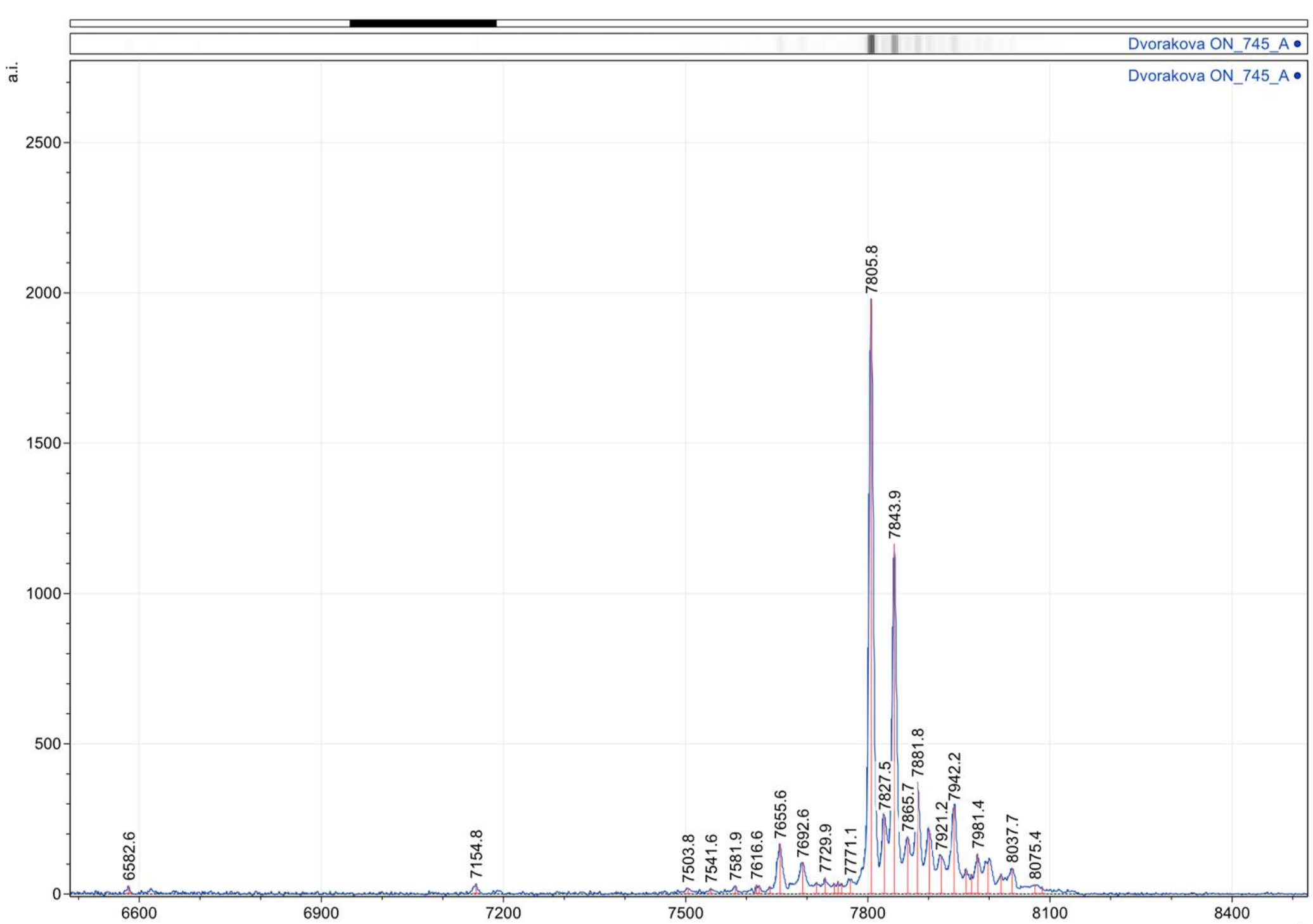

Dvorakova ON_745_A
a.i.
m/z
7805.8
7843.9
7881.8
7942.2
7827.5
7865.7
7921.2
7981.4
8037.7
8075.4
7655.6
7692.6
7729.9
7771.1
7616.6
7581.9
7541.6
7503.8
7154.8
6582.6

ODN8

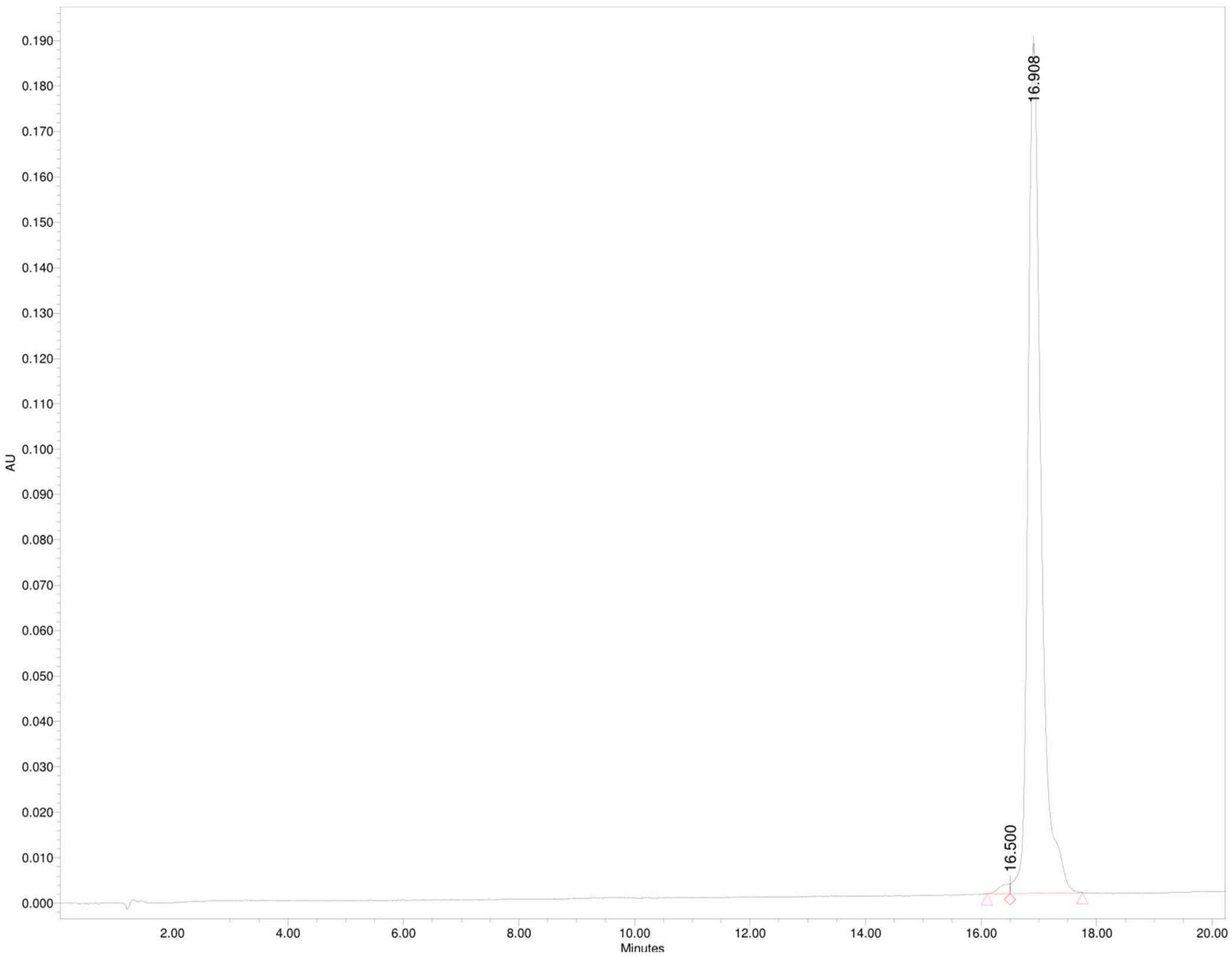

16.908
16.500
AU
Minutes

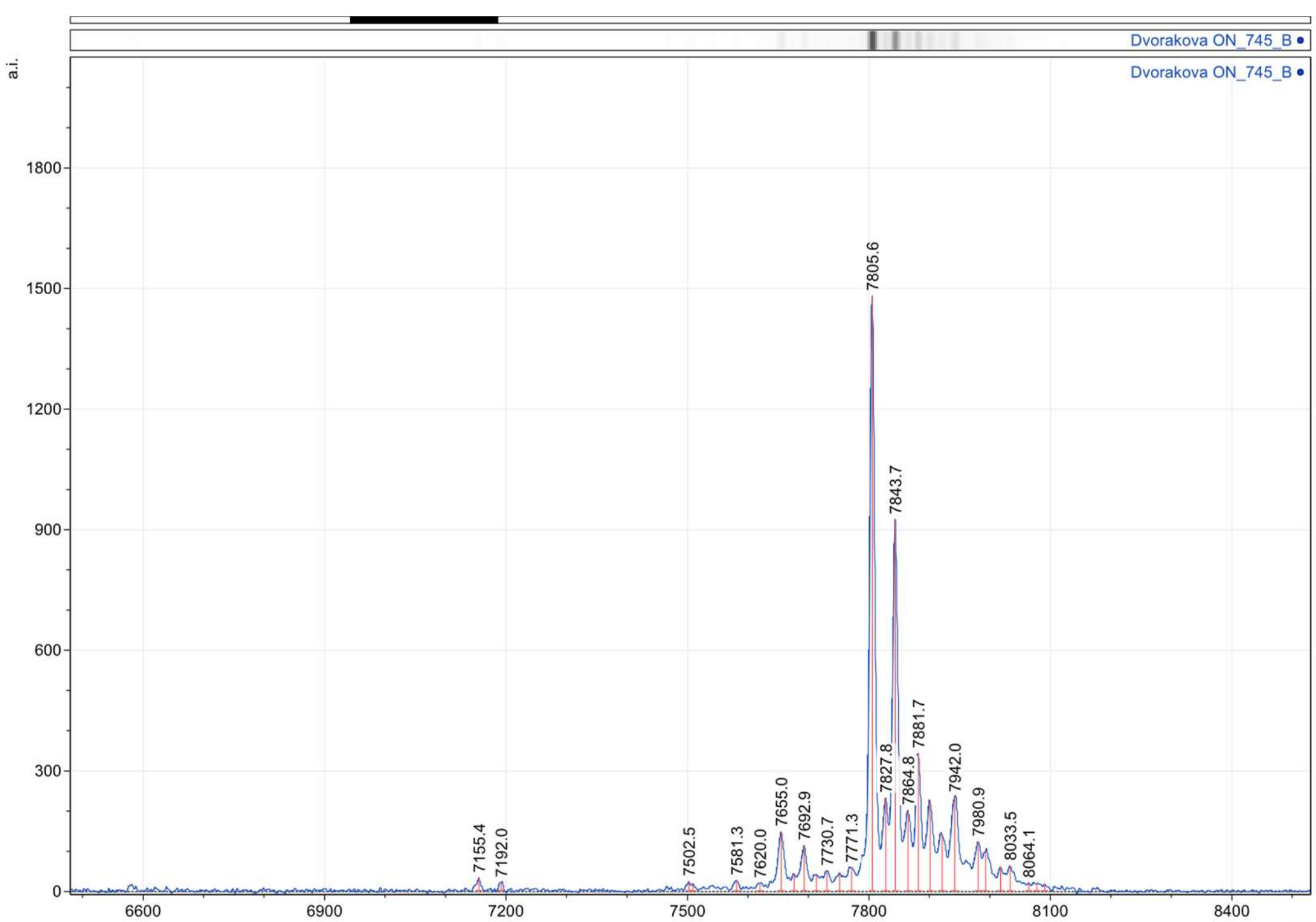

Dvorakova ON_745_B
a.i.
7805.6
7843.7
7881.7
7827.8
7864.8
7942.0
7980.9
8033.5
8064.1
7655.0
7692.9
7730.7
7771.3
7620.0
7581.3
7502.5
7155.4
7192.0
m/z

ODN9

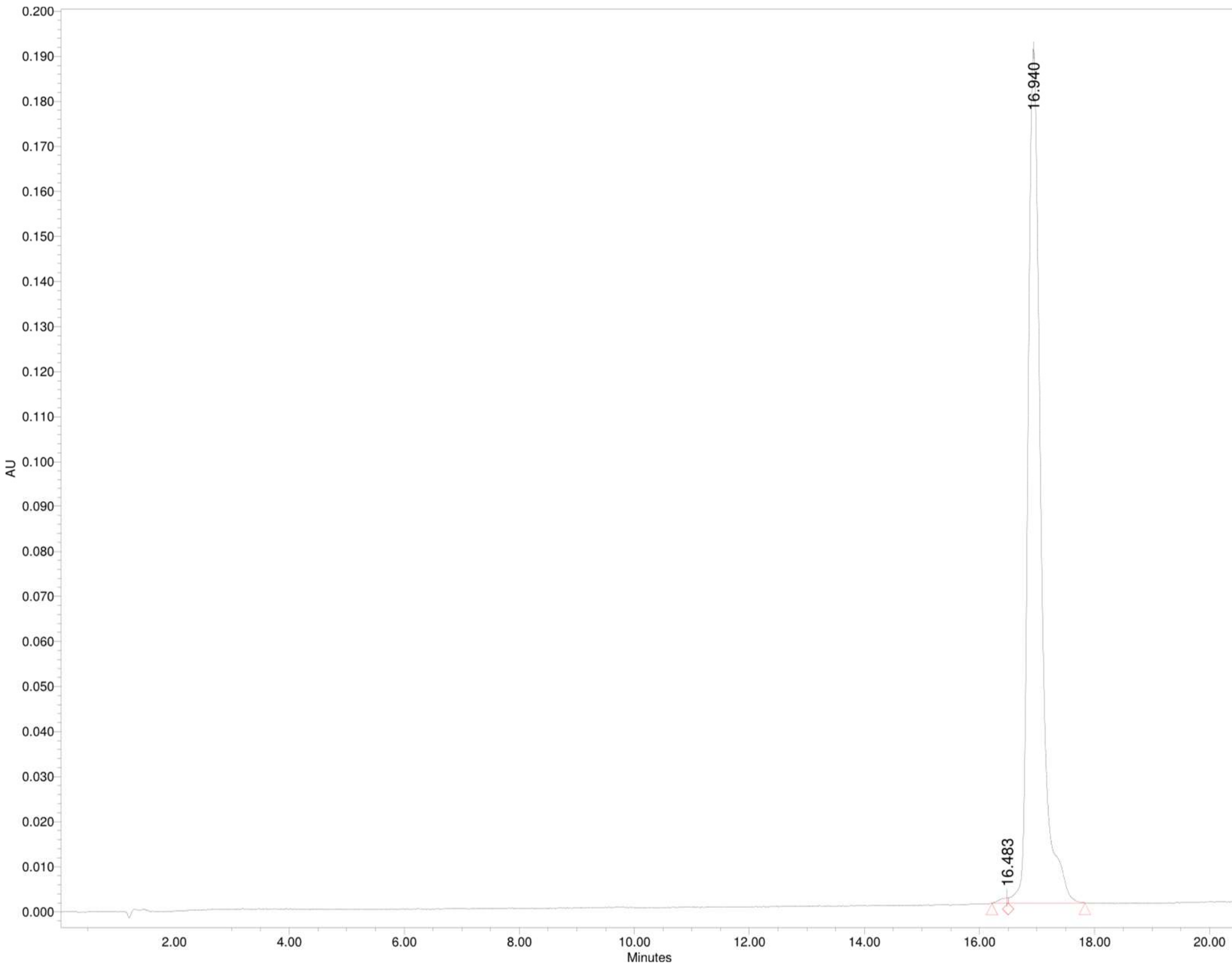

16.940
16.483
AU
Minutes

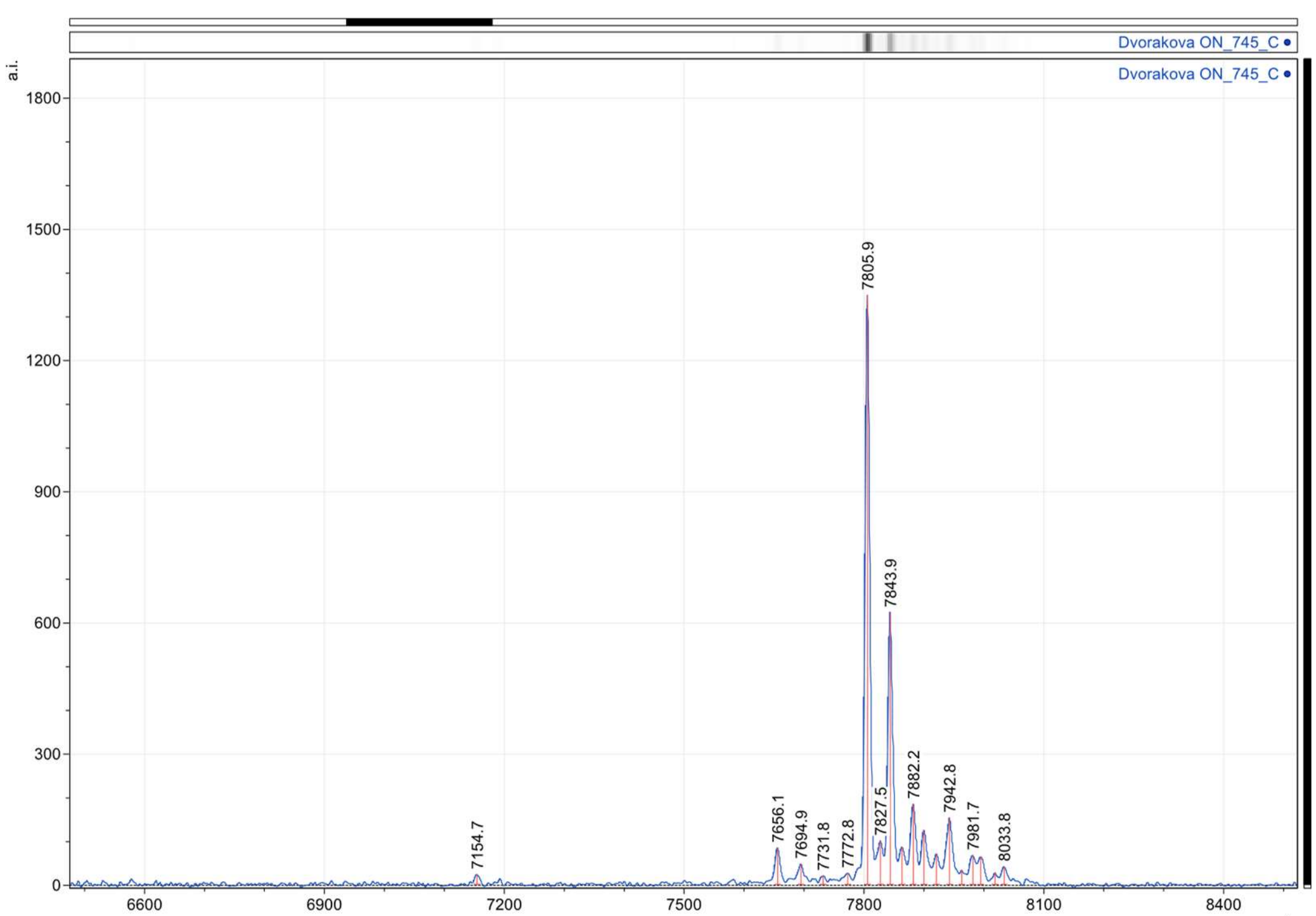

Dvorakova ON_745_C
a.i.
7805.9
7843.9
7882.2
7942.8
7827.5
7981.7
8033.8
7656.1
7694.9
7731.8
7772.8
7154.7
m/z

ODN10

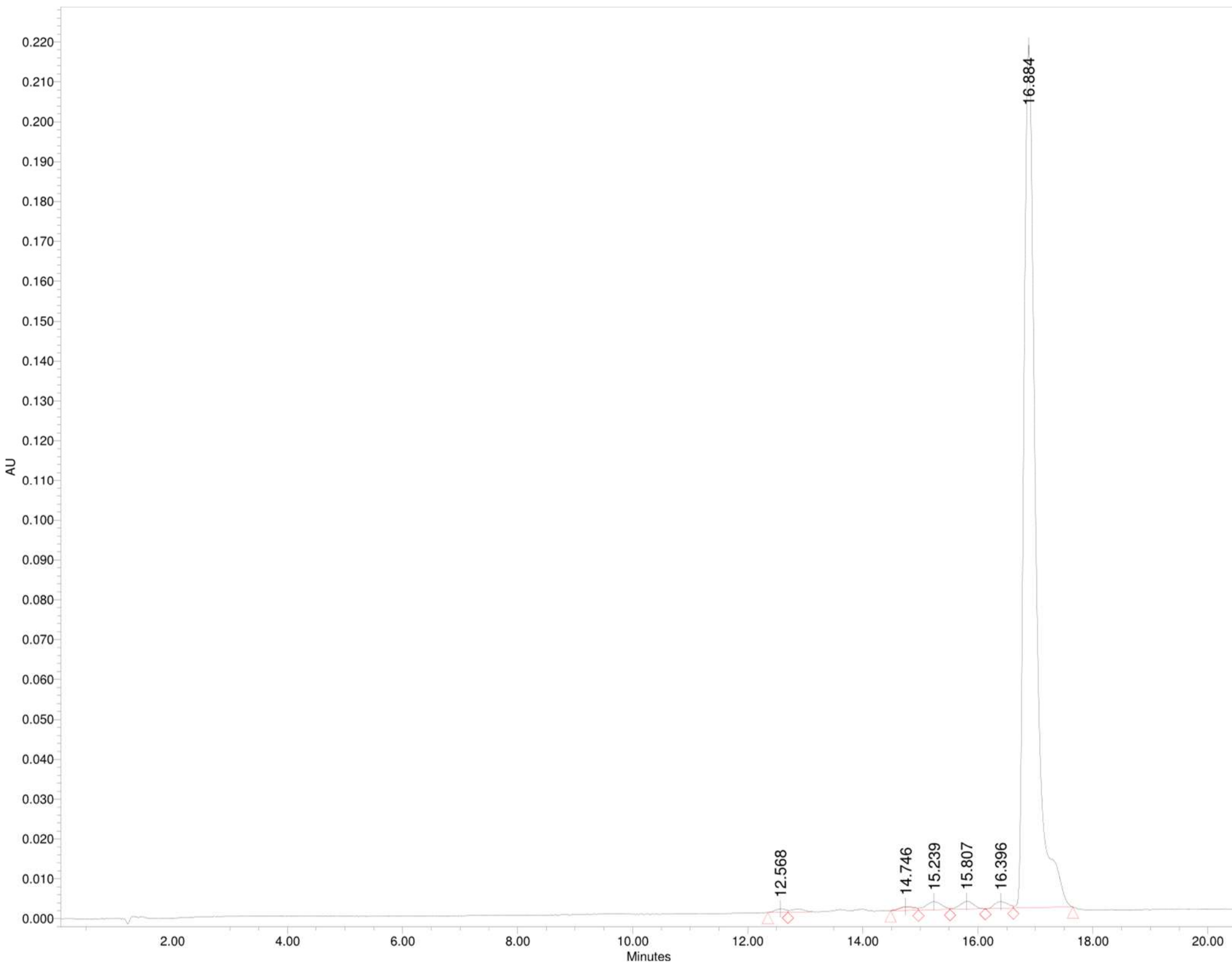

AU
Minutes
16.884
12.568
14.746
15.239
15.807
16.396

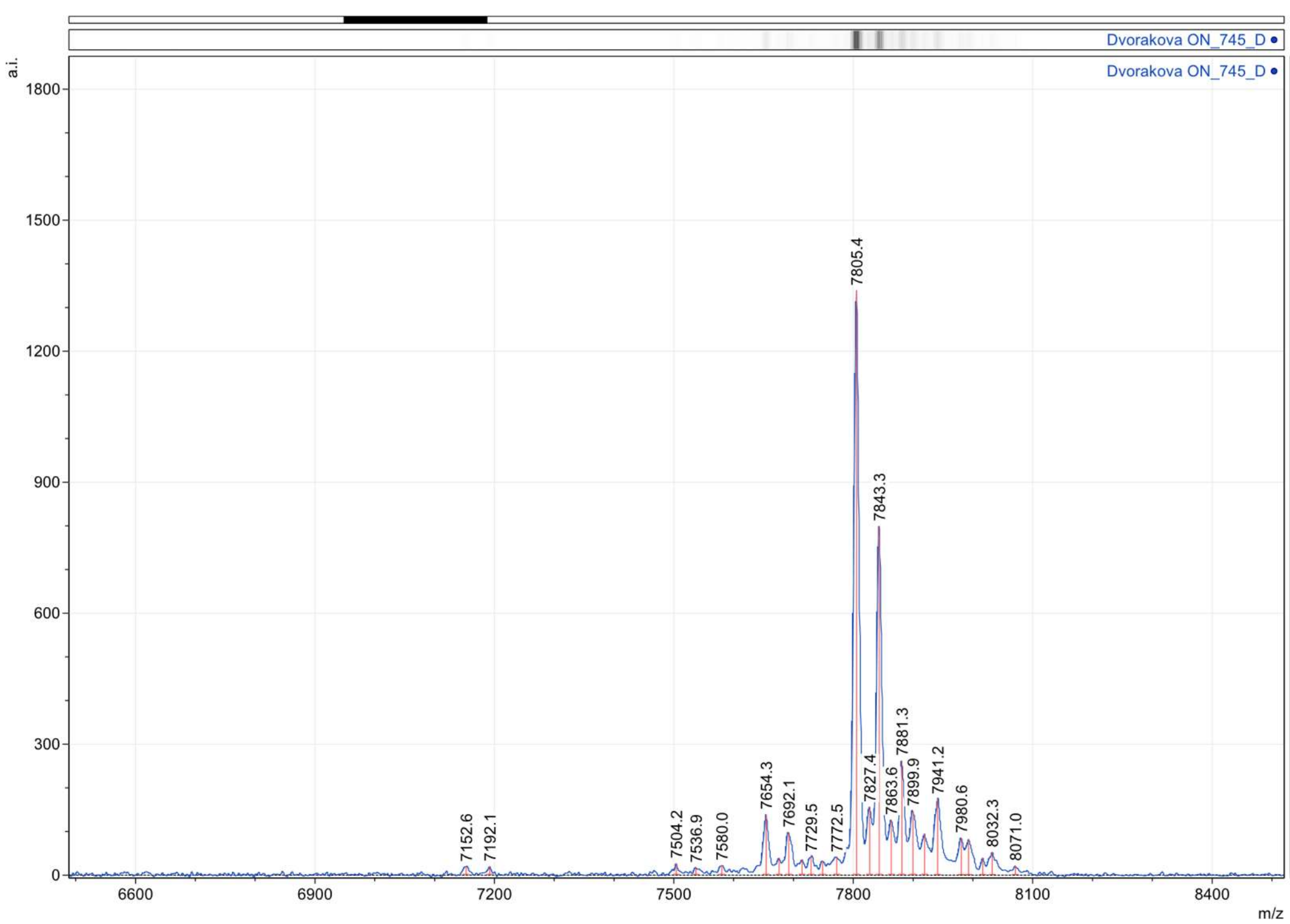

Dvorakova ON_745_D
a.i.
m/z
7805.4
7843.3
7881.3
7827.4
7863.6
7899.9
7941.2
7980.6
8032.3
8071.0
7654.3
7692.1
7729.5
7772.5
7504.2
7536.9
7580.0
7152.6
7192.1

ODN11

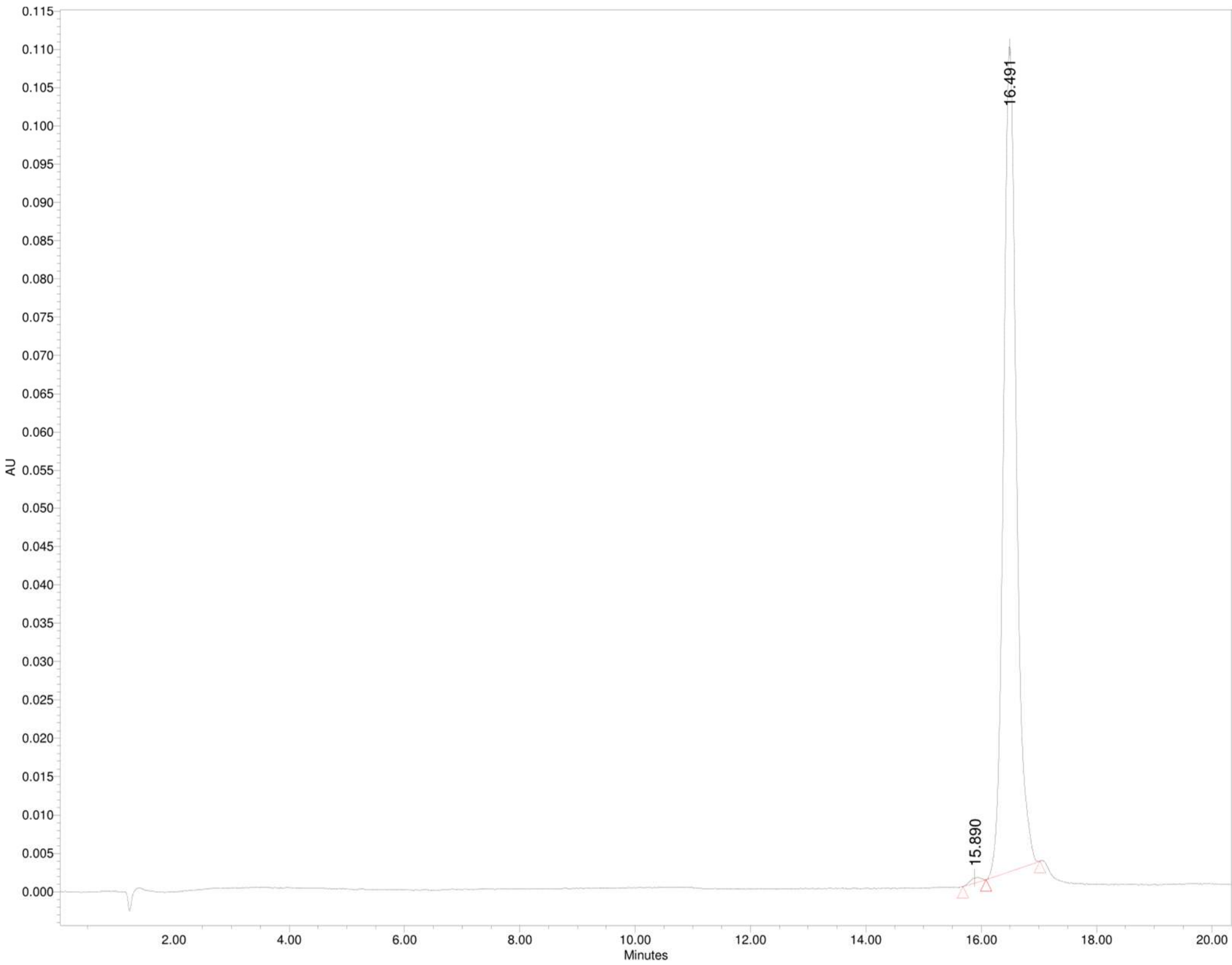

16.491
15.890
AU
Minutes

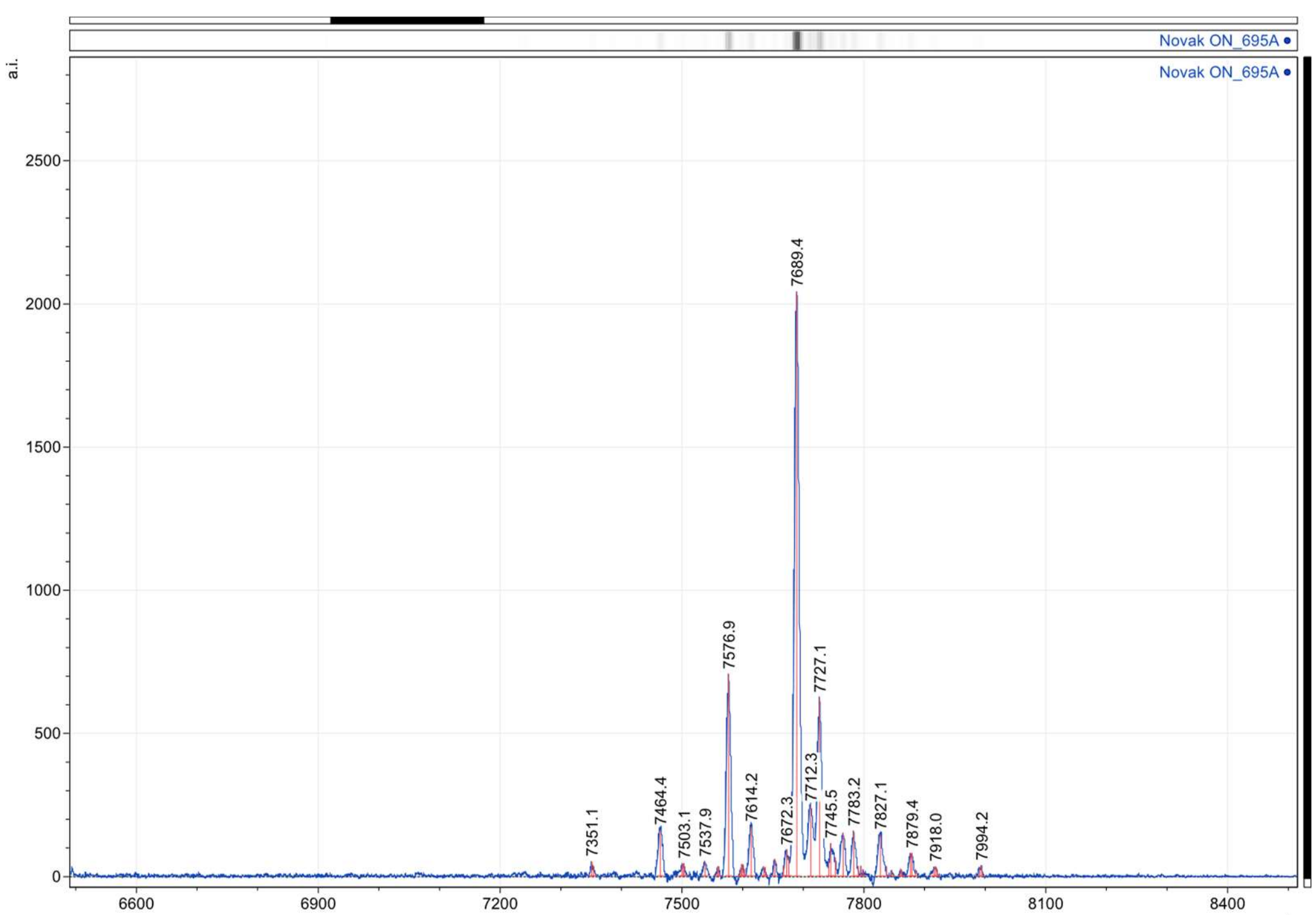

Novak ON_695A
a.i.
7689.4
7576.9
7727.1
7464.4
7351.1
7503.1
7537.9
7614.2
7672.3
7712.3
7745.5
7783.2
7827.1
7879.4
7918.0
7994.2
m/z

ODN12

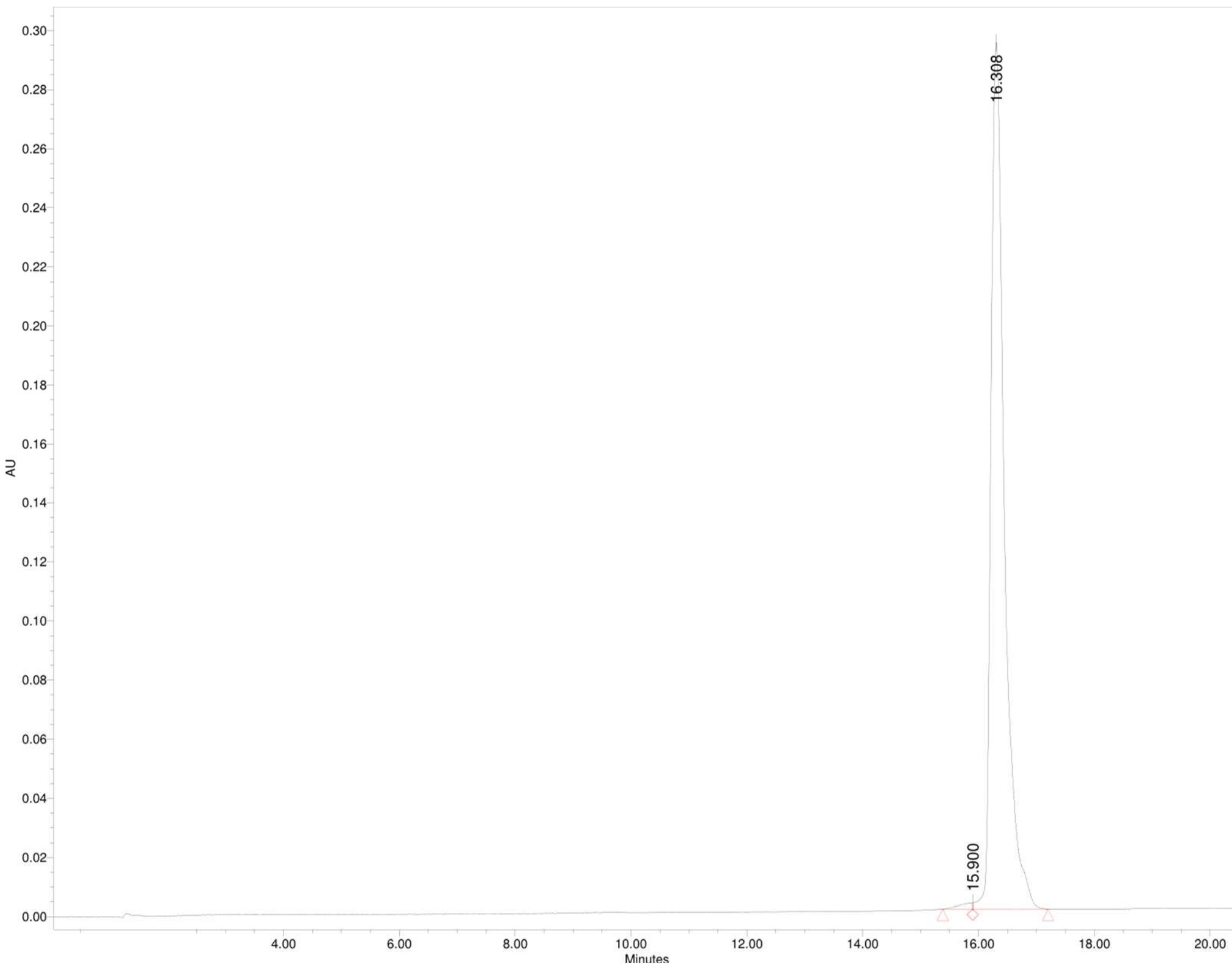

16.308
15.900
AU
Minutes
0.00
0.02
0.04
0.06
0.08
0.10
0.12
0.14
0.16
0.18
0.20
0.22
0.24
0.26
0.28
0.30
4.00
6.00
8.00
10.00
12.00
14.00
16.00
18.00
20.00

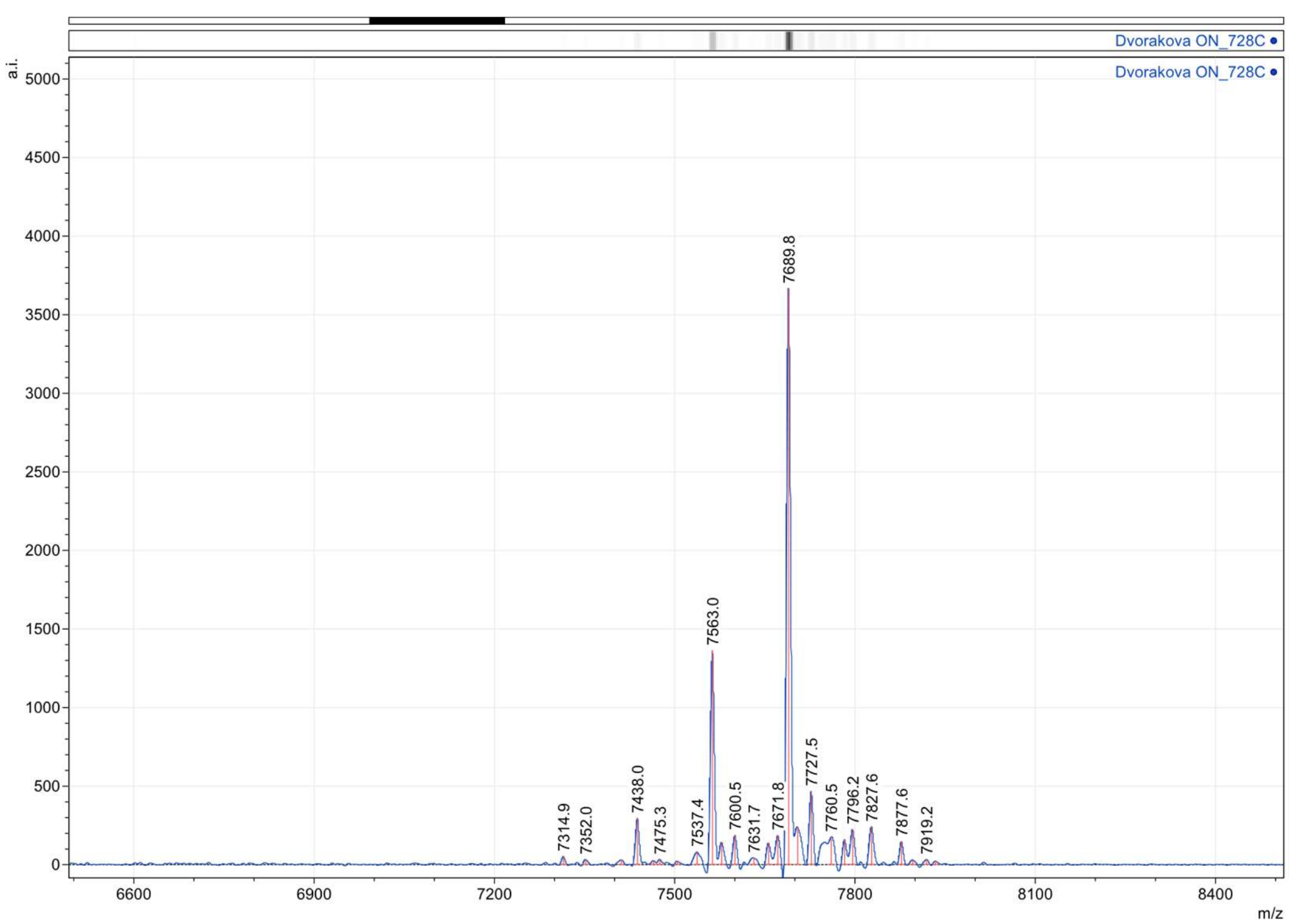

Dvorakova ON_728C
Dvorakova ON_728C
a.i.
m/z
7689.8
7563.0
7314.9
7352.0
7438.0
7475.3
7537.4
7600.5
7631.7
7671.8
7727.5
7760.5
7796.2
7827.6
7877.6
7919.2
0
500
1000
1500
2000
2500
3000
3500
4000
4500
5000
6600
6900
7200
7500
7800
8100
8400

ODN13

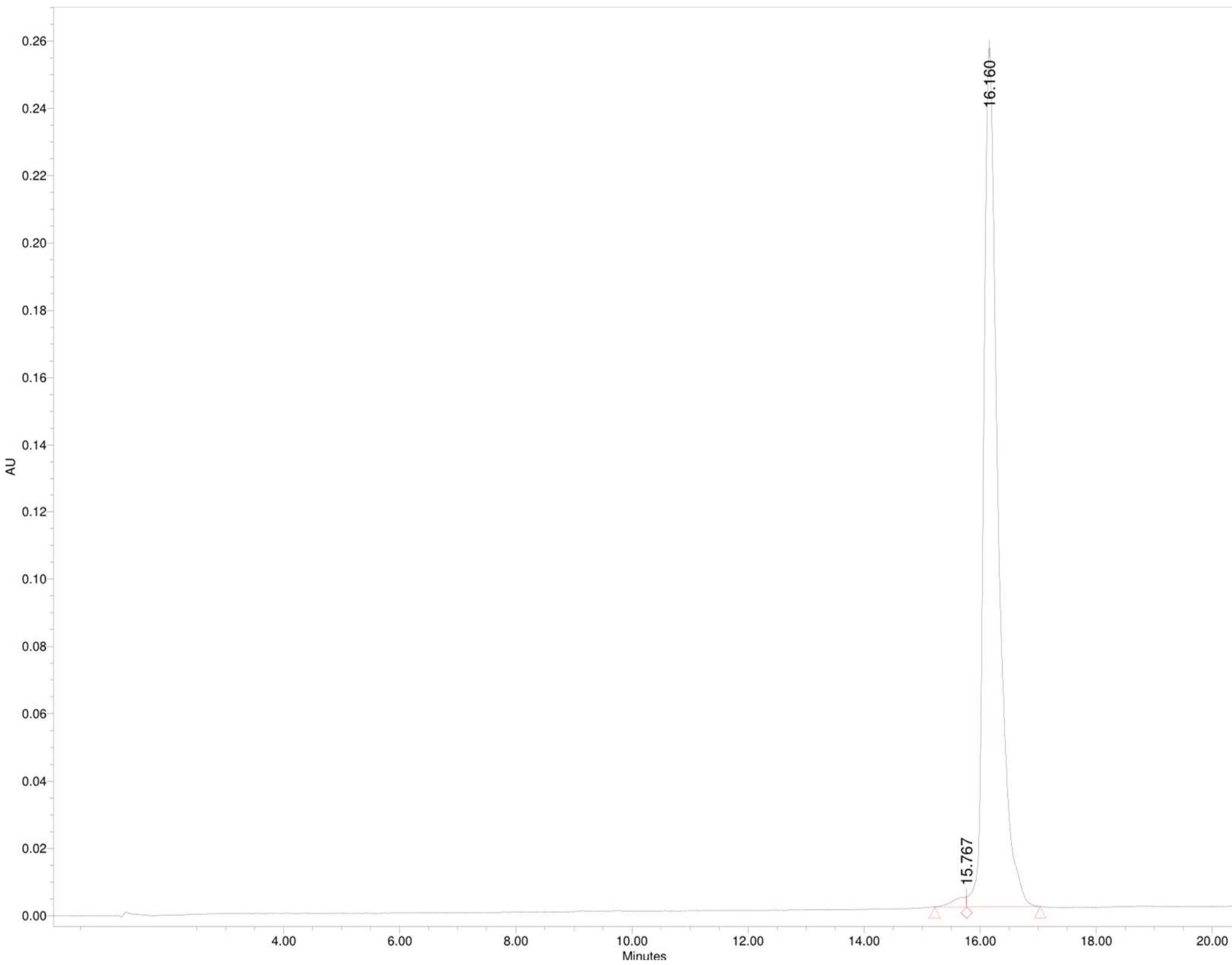

16.160
15.767
AU
Minutes
0.00
0.02
0.04
0.06
0.08
0.10
0.12
0.14
0.16
0.18
0.20
0.22
0.24
0.26
4.00
6.00
8.00
10.00
12.00
14.00
16.00
18.00
20.00

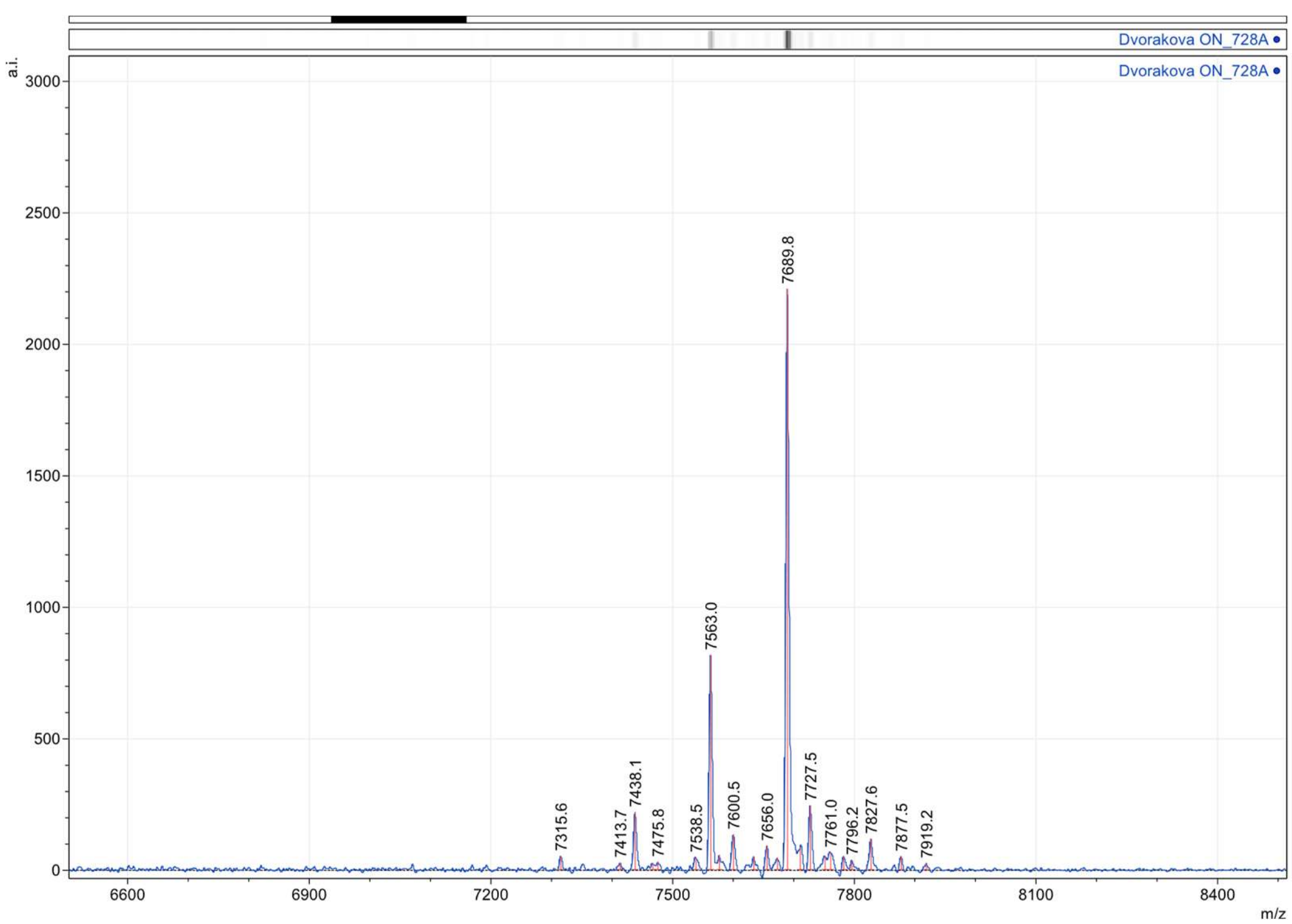

Dvorakova ON_728A
a.i.
m/z
7689.8
7563.0
7315.6
7413.7
7438.1
7475.8
7538.5
7600.5
7656.0
7727.5
7761.0
7796.2
7827.6
7877.5
7919.2
0
500
1000
1500
2000
2500
3000
6600
6900
7200
7500
7800
8100
8400

ODN14

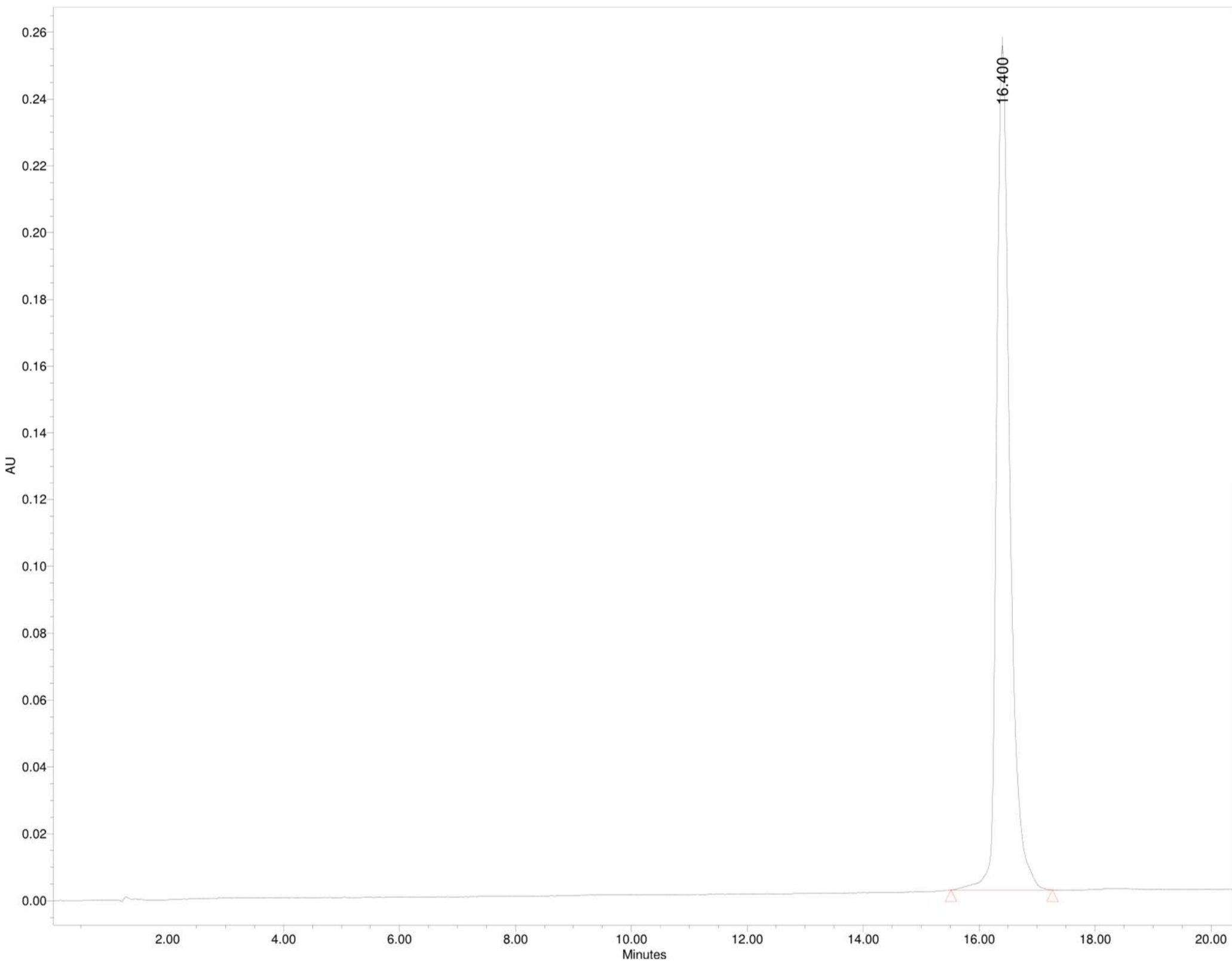

16.400
AU
Minutes

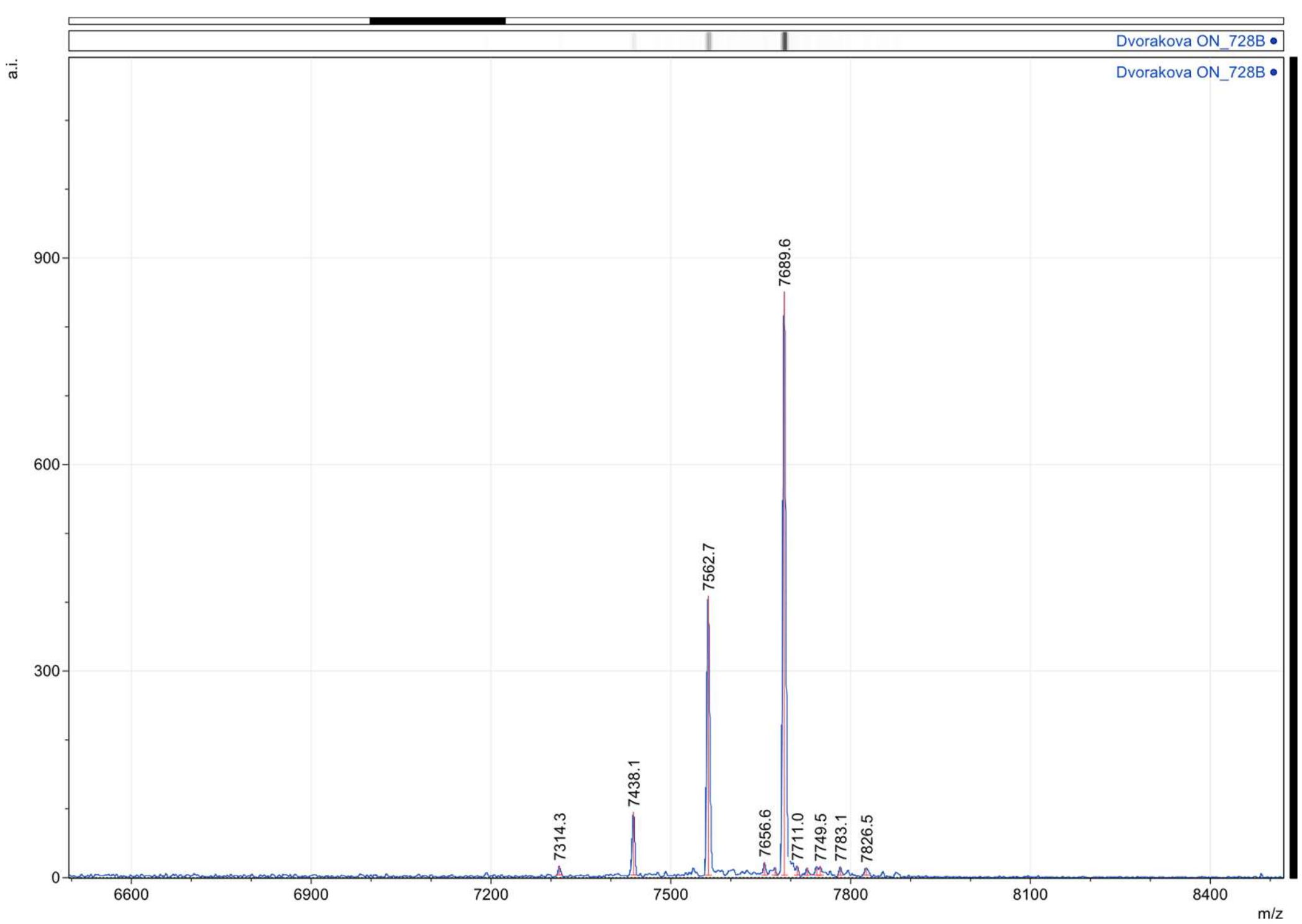

Dvorakova ON_728B
a.i.
7689.6
7562.7
7438.1
7314.3
7656.6
7711.0
7749.5
7783.1
7826.5
m/z

ODN15

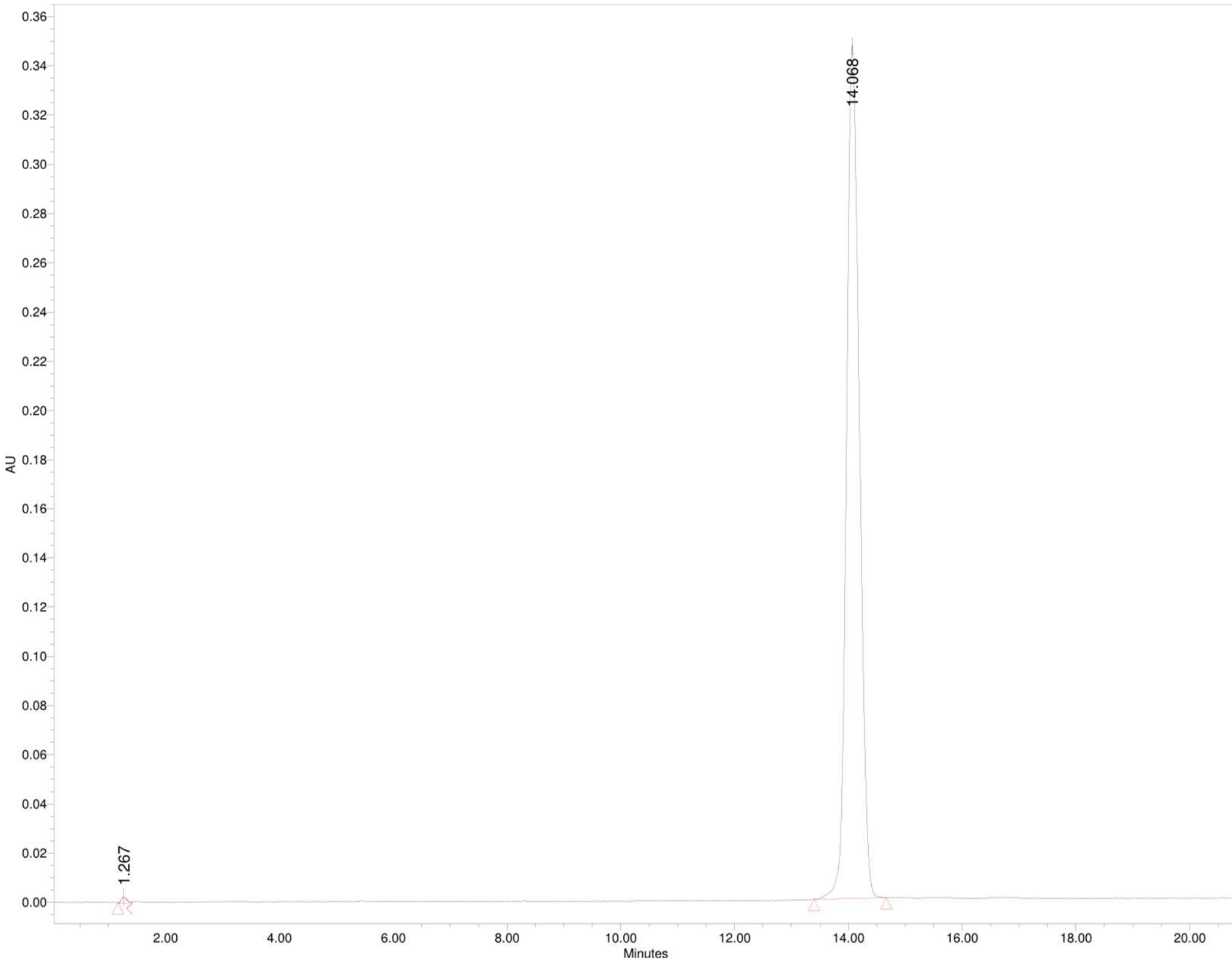
14.068
1.267
AU
Minutes
0.36
0.34
0.32
0.30
0.28
0.26
0.24
0.22
0.20
0.18
0.16
0.14
0.12
0.10
0.08
0.06
0.04
0.02
0.00
2.00
4.00
6.00
8.00
10.00
12.00
14.00
16.00
18.00
20.00

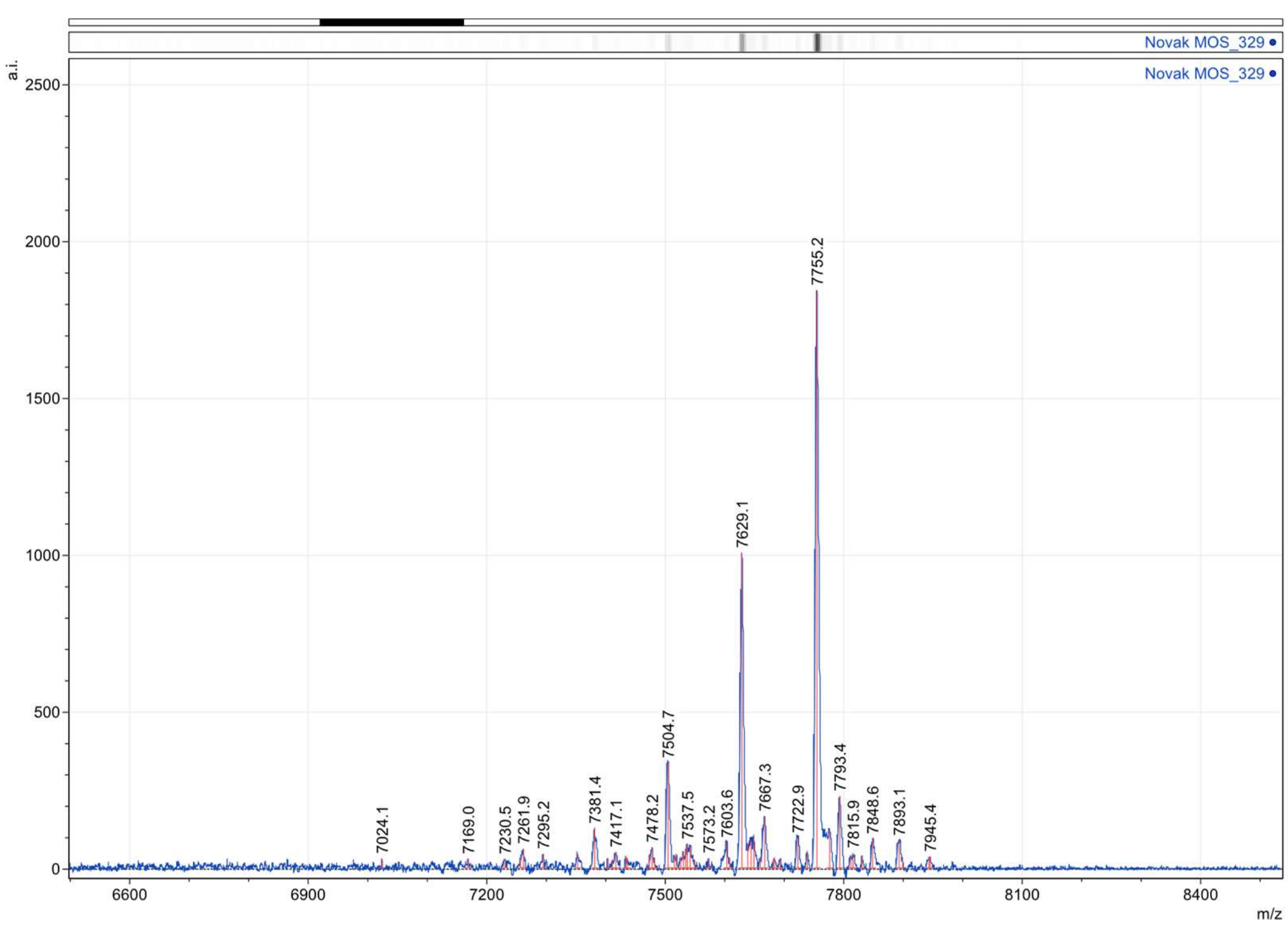
Novak MOS_329
Novak MOS_329
a.i.
m/z
7755.2
7629.1
7504.7
7793.4
7667.3
7381.4
7024.1
7169.0
7230.5
7261.9
7295.2
7417.1
7478.2
7537.5
7573.2
7603.6
7722.9
7815.9
7848.6
7893.1
7945.4
2500
2000
1500
1000
500
0
6600
6900
7200
7500
7800
8100
8400

ODN16

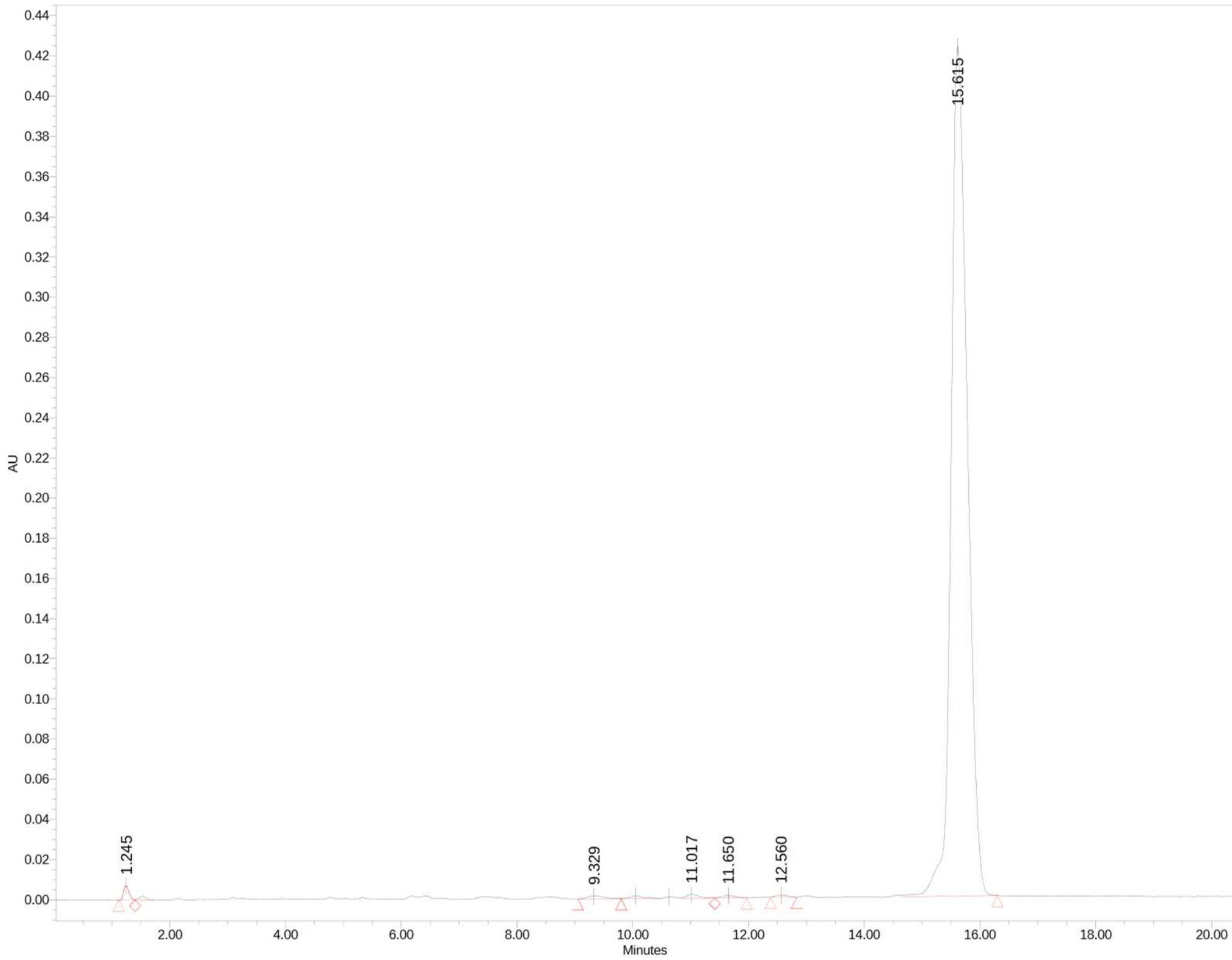

AU
Minutes
15.615
1.245
9.329
11.017
11.650
12.560

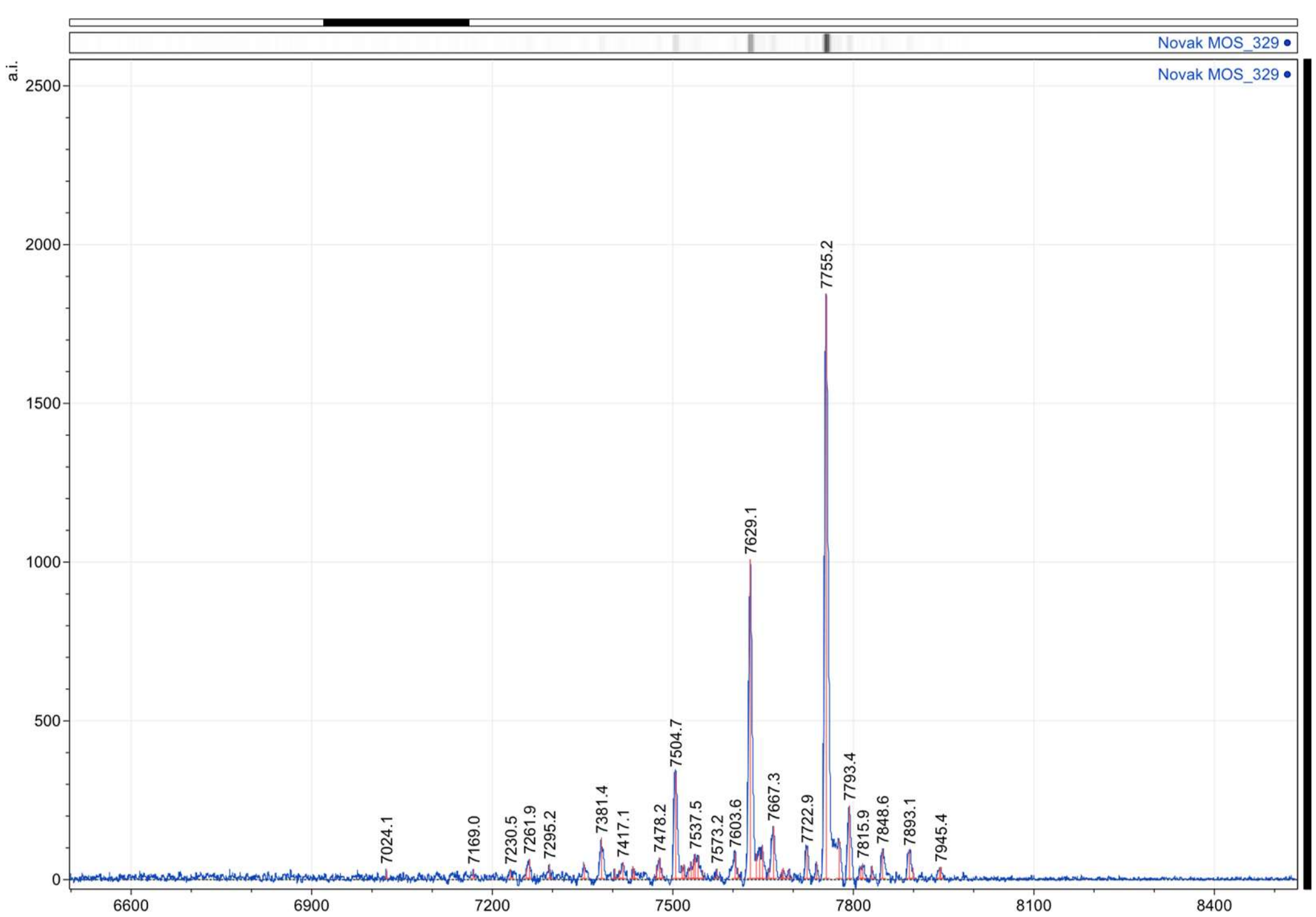

Novak MOS_329
a.i.
m/z
7755.2
7629.1
7504.7
7793.4
7381.4
7667.3
7024.1
7169.0
7230.5
7261.9
7295.2
7417.1
7478.2
7537.5
7573.2
7603.6
7722.9
7815.9
7848.6
7893.1
7945.4

ODN-A

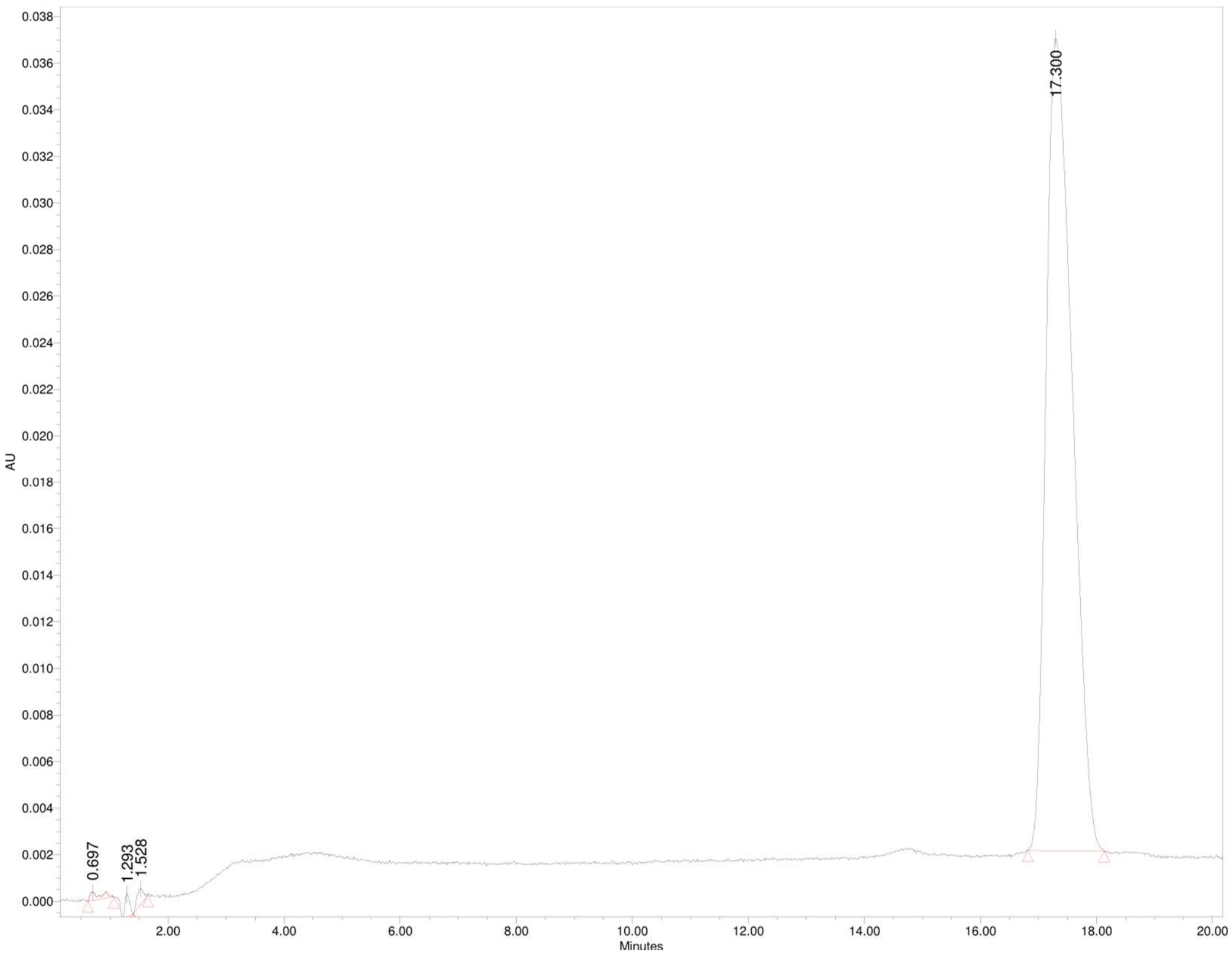

AU
Minutes
0.697
1.293
1.528
17.300

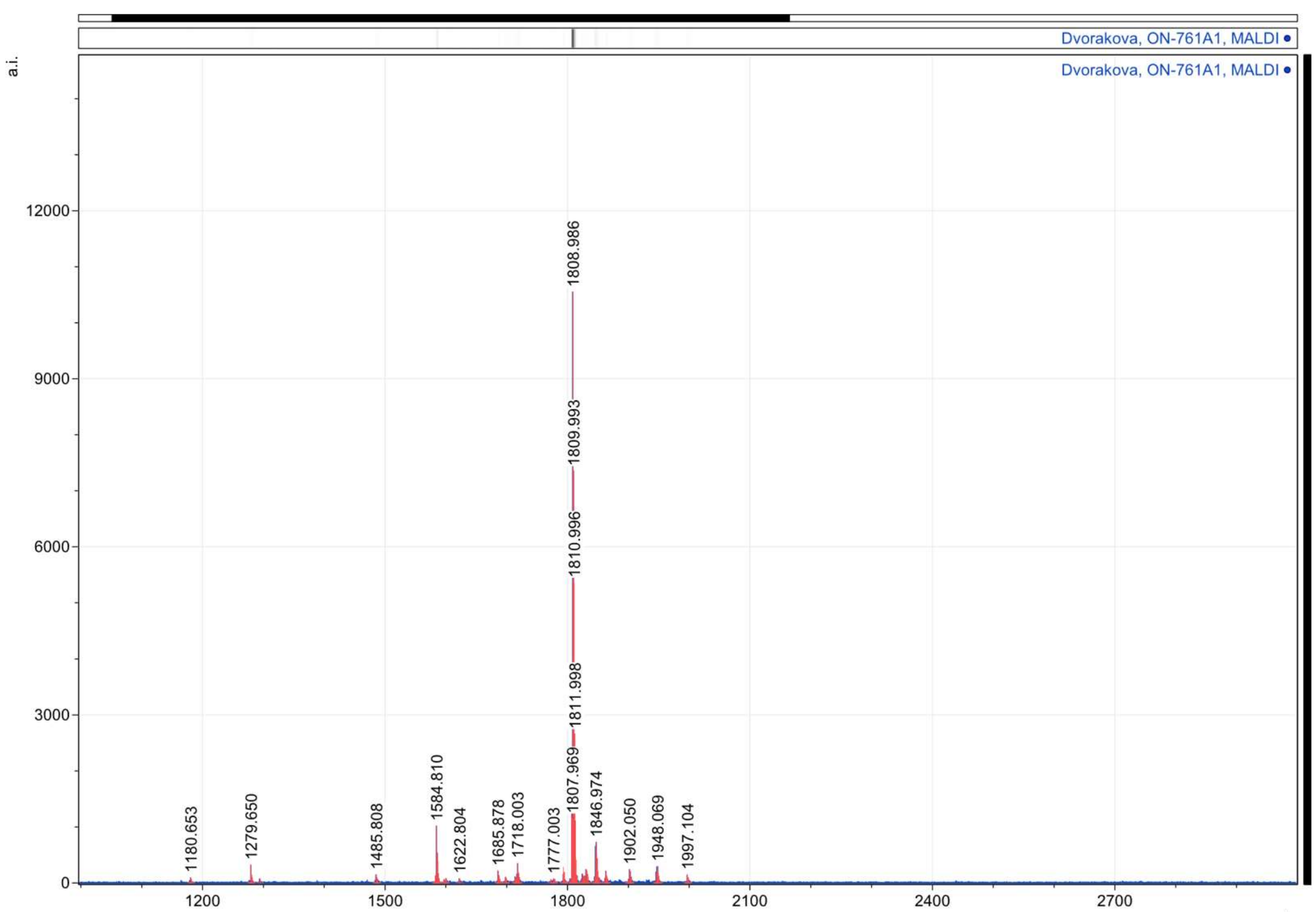

Dvorakova, ON-761A1, MALDI
a.i.
m/z
1808.986
1809.993
1810.996
1811.998
1807.969
1584.810
1846.974
1279.650
1180.653
1485.808
1622.804
1685.878
1718.003
1777.003
1902.050
1948.069
1997.104

ODN-B

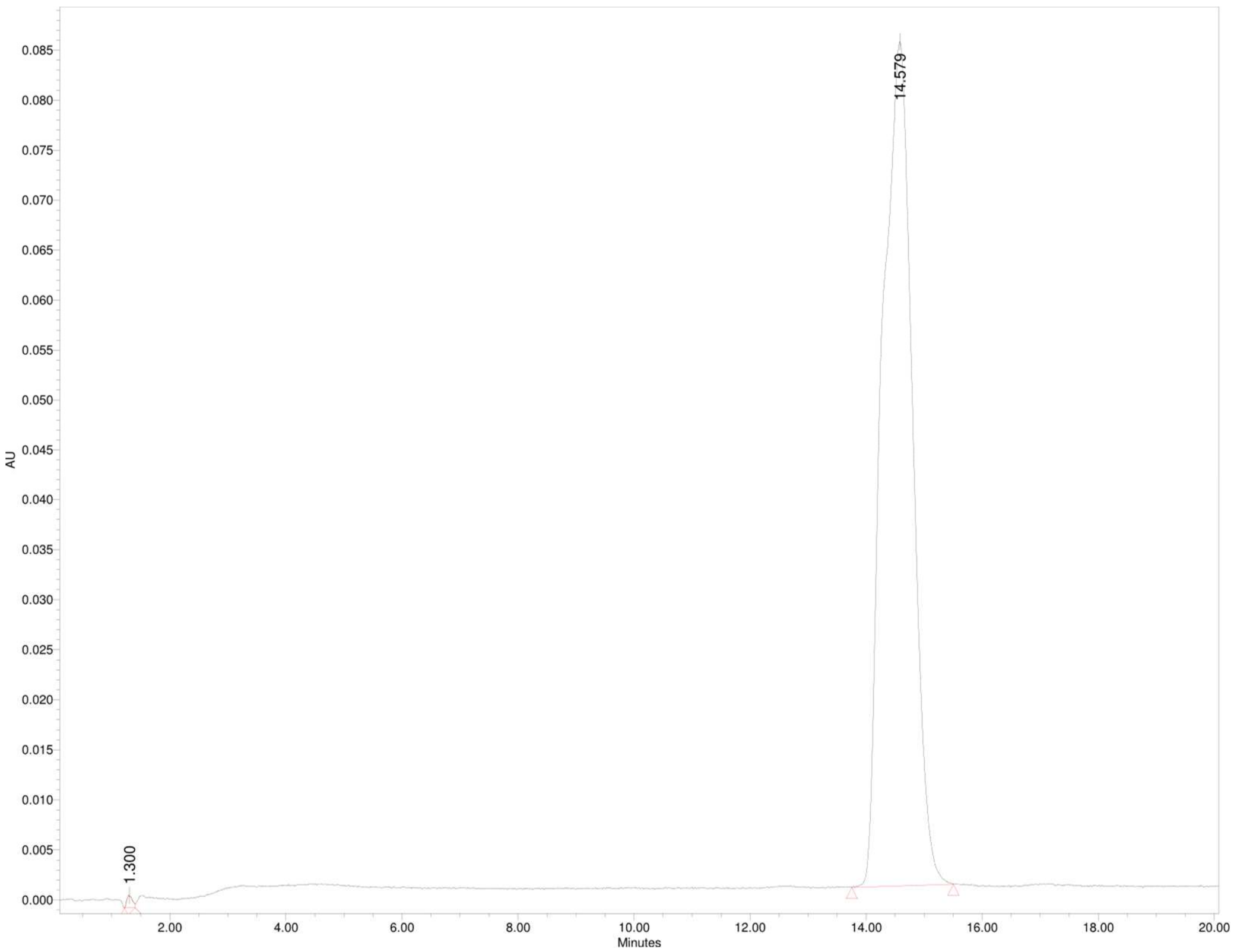

AU
Minutes
14.579
1.300
0.085
0.080
0.075
0.070
0.065
0.060
0.055
0.050
0.045
0.040
0.035
0.030
0.025
0.020
0.015
0.010
0.005
0.000
2.00
4.00
6.00
8.00
10.00
12.00
14.00
16.00
18.00
20.00

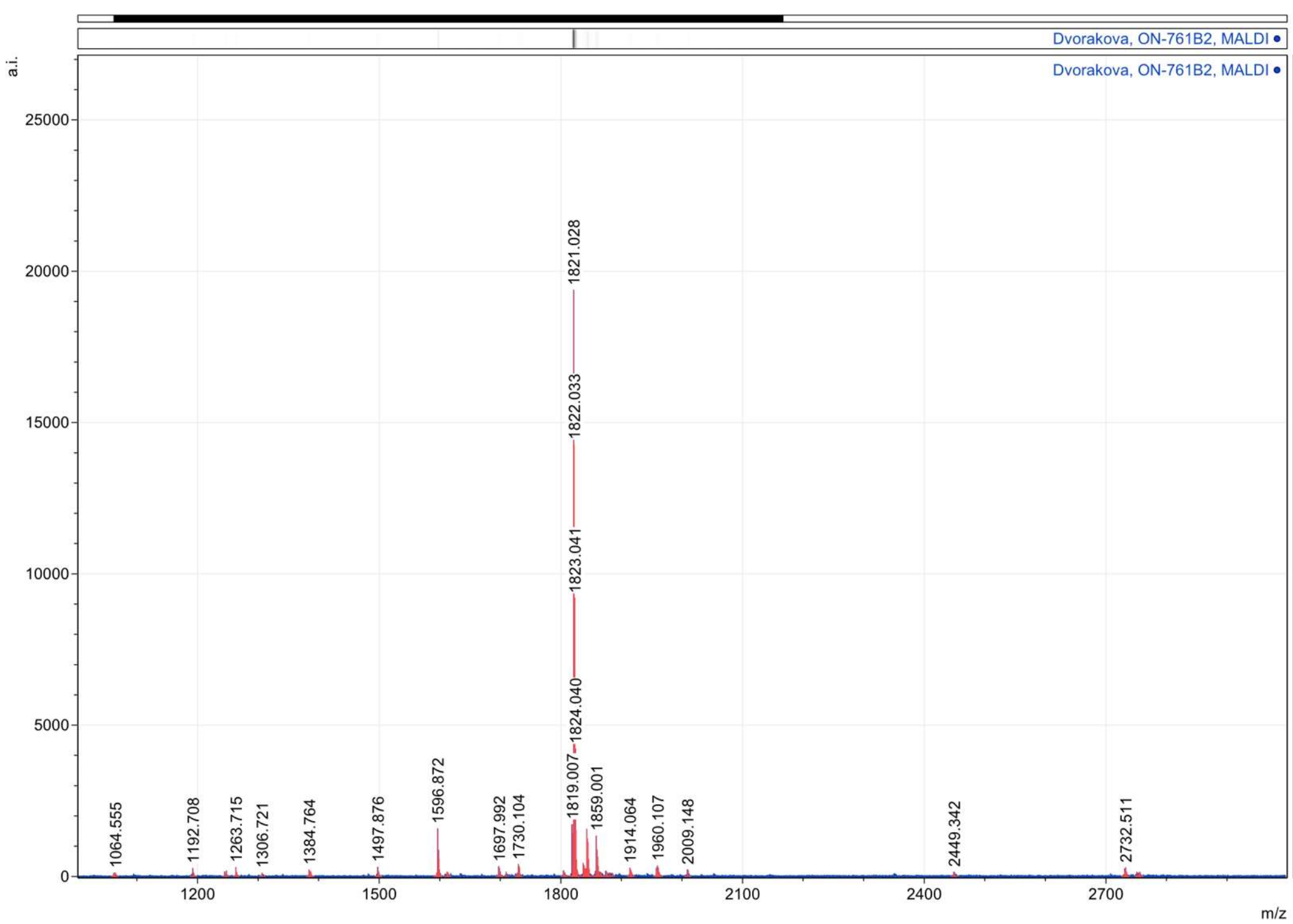

Dvorakova, ON-761B2, MALDI
a.i.
m/z
1821.028
1822.033
1823.041
1824.040
1819.007
1859.001
1596.872
1064.555
1192.708
1263.715
1306.721
1384.764
1497.876
1697.992
1730.104
1914.064
1960.107
2009.148
2449.342
2732.511
25000
20000
15000
10000
5000
0
1200
1500
1800
2100
2400
2700

ODN-C

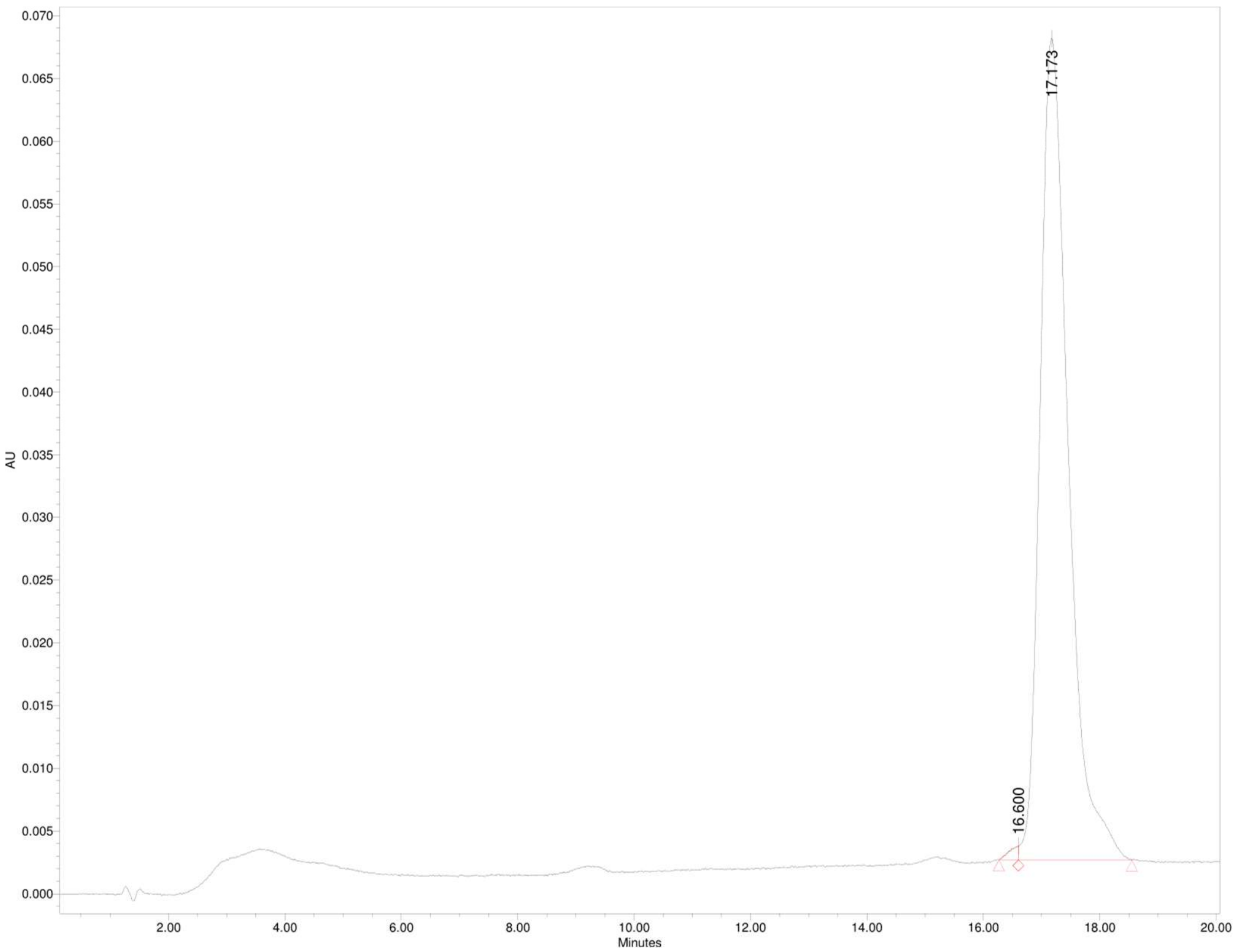

17.173
16.600
AU
Minutes

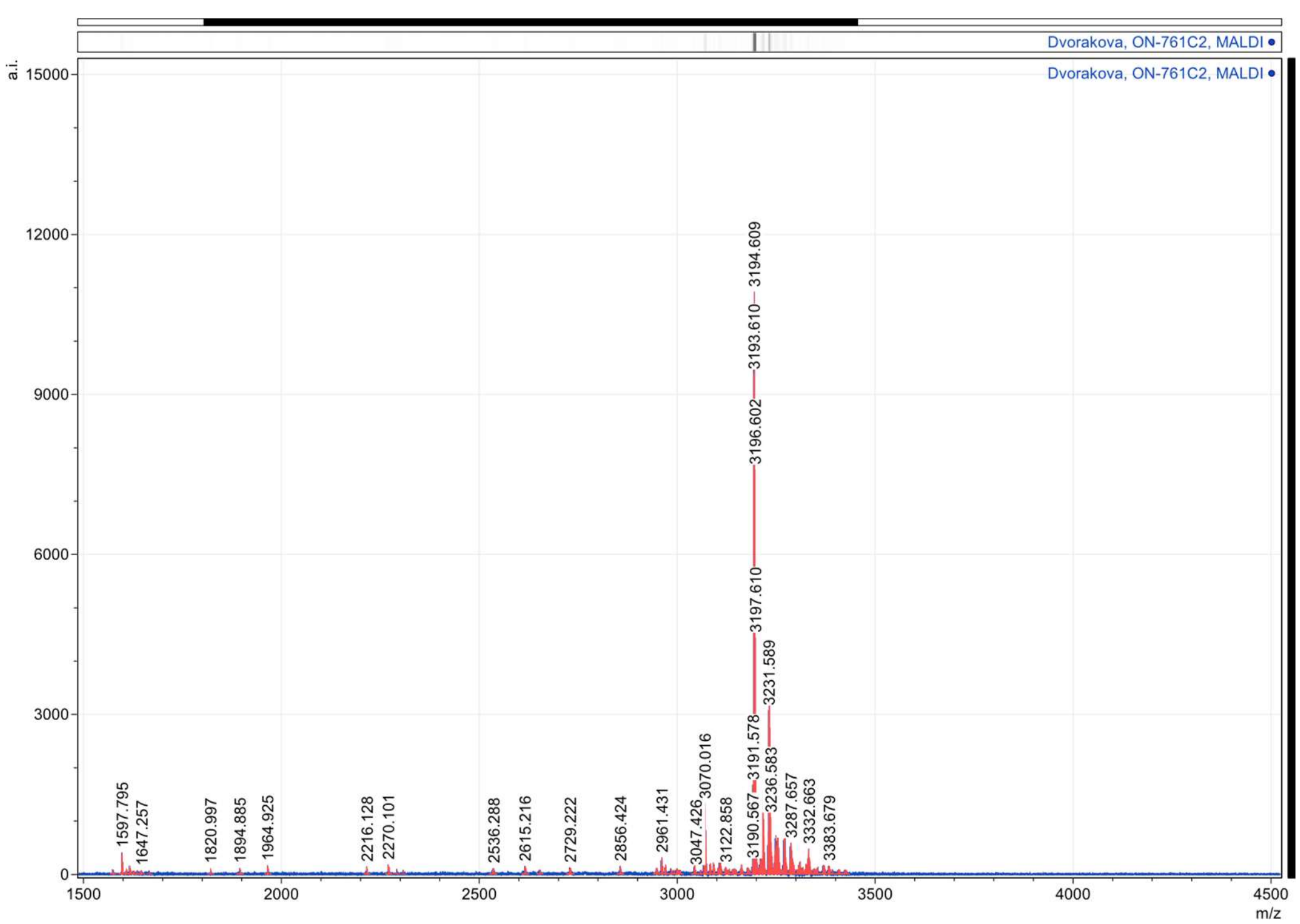

Dvorakova, ON-761C2, MALDI
a.i.
m/z
3194.609
3193.610
3196.602
3197.610
3231.589
3070.016
3191.578
3236.583
3287.657
3332.663
3383.679
3190.567
3122.858
3047.426
2961.431
2856.424
2729.222
2615.216
2536.288
2270.101
2216.128
1964.925
1894.885
1820.997
1647.257
1597.795

ODN stability in human serum

Solution of **ODN1**, **ODN2** and **ODN 2006** (1 nmol) in 0.05 M TRIS-HCl buffer (50 µL, pH 7.5 with 0.01 M magnesium sulphate was incubated for 20 hours with human serum (50mL) at 37 °C. Subsequent IE HPLC analyses showed complete nuclease stability of both the parental **ODN 2006** and its analogues under these conditions.

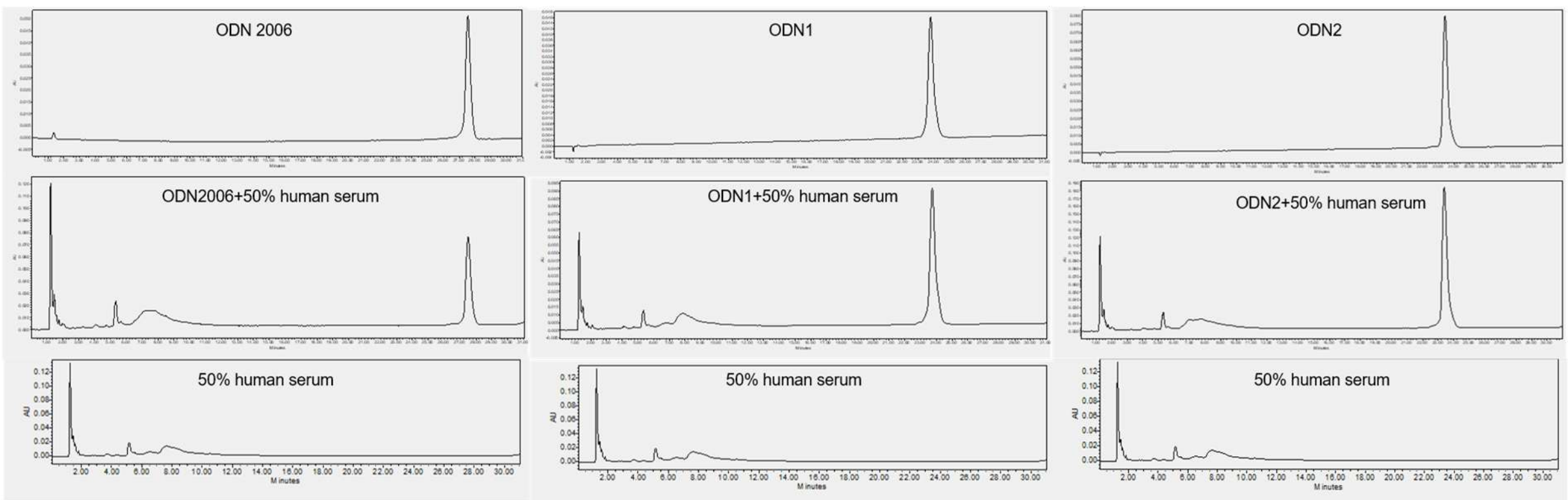

Figure S1 Stability of selected ODN in human serum. IE HPLC analysis: column: DNAPac PA100 (Dionex) 4.6 x 250 mm, elution: 0→0.3 M $NaClO_4$/30 min in 0.05 M TRIS-HCl (pH 7.5) containing 10 % (v/v) ACN; 1 mL/min; 80 °C, detection: $UV_{254\ nm}$

Abbreviations

ACN	Acetonitrile
AIBN	2,2′-Azobis(isobutyronitrile)
Bz	Benzoyl
DBAM	Dibutylaminomethylene
DCM	Dichloromethane
DMAP	4-(Dimethylamino)pyridine
DMF	*N,N*-Dimethylformamide
DMTr	4,4′-Dimethoxytrityl
DMTrCl	4,4′-Dimethoxytrityl chloride
DMSO	Dimethyl sulfoxide
HPLC	High-performance liquid chromatography
IE-HPLC	Ion-exchange high-performance liquid chromatography
LC-MS	Liquid chromatography–mass spectrometry
MOP	4-Hydroxy-1-oxido-2-pyridylmethyl
MOP-OH	2-(Hydroxymethyl)-4-methoxypyridine *N*-oxide
MsCl	Methanesulfonyl chloride
NEP-Cl	2-Chloro-5,5-dimethyl-1,3,2-dioxaphosphorinane 2-oxide
ODN	Oligodeoxynucleotide
TBAF	Tetrabutylammonium fluoride
TBDPS	tert-Butyldiphenylsilyl
TEAB	Triethylammonium bicarbonate
THF	Tetrahydrofuran
TiPBSCl	tert-Butyldiphenylsilyl chloride
TMSBr	Bromotrimethylsilane
TMSOTf	Trimethylsilyl trifluoromethanesulfonate
TsOH	*p*-Toluenesulfonic acid

References:

(1) Endová, M.; Masojídková, M.; Buděšínský, M.; Rosenberg, I. 2’,3’-O-Phosphonoalkylidene Derivatives of Ribonucleosides: Synthesis and Reactivity. *Tetrahedron* **1998**, *54* (37), 11151–11186. https://doi.org/10.1016/S0040-4020(98)00653-X.

(2) Páv, O.; Barvík, I.; Liboska, R.; Petrová, M.; Šimák, O.; Rosenbergová, Š.; Novák, P.; Buděšínský, M.; Rosenberg, I. Tuning the Hybridization Properties of Modified Oligonucleotides: From Flexible to Conformationally Constrained Phosphonate Internucleotide Linkages. *Org. Biomol. Chem.* **2017**, *15* (3), 701–707. https://doi.org/10.1039/c6ob02571d.

(3) Kóšiová, I.; Šimák, O.; Panova, N.; Buděšínský, M.; Petrová, M.; Rejman, D.; Liboska, R.; Páv, O.; Rosenberg, I. Inhibition of Human Thymidine Phosphorylase by Conformationally Constrained Pyrimidine Nucleoside Phosphonic Acids and Their “Open-Structure” Isosteres. *Eur. J. Med. Chem.* **2014**, *74*, 145–168. https://doi.org/10.1016/j.ejmech.2013.12.026.

(4) Rhie, S. Y.; Pfleiderer, W. Nucleosides. Lvi1synthesis and Chemical Modifications of 3’-Deoxy-Pyrimidine Nucleosides. *Nucleosides Nucleotides* **1994**, *13* (6–7), 1425–1452. https://doi.org/10.1080/15257779408012162.

(5) Seela, F.; Wörner, K.; Rosemeyer, H. 1-(2′-Deoxy-β-D-xylofuranosyl)Cytosine: Base Pairing of Oligonucleotides with a Configurationally Altered Sugar-phosphate Backbone. *Helv. Chim. Acta* **1994**, *77* (4), 883–896. https://doi.org/10.1002/hlca.19940770402.

(6) Kawana, M.; Kuzuhara, H. General Method for the Synthesis of 2’-Azido-2’,3’-Dideoxynucleosides by the Use of [1,2]-Hydride Shift and β-Elimination Reactions. *J. Chem. Soc., Perkin Trans. 1* **1992**, No. 4, 469–478. https://doi.org/10.1039/P19920000469.

(7) Kóšiová, I.; Točík, Z.; Buděšínský, M.; Šimák, O.; Liboska, R.; Rejman, D.; Pačes, O.; Rosenberg, I. Methyl 4-Toluenesulfonyloxymethylphosphonate, a New and Versatile Reagent for the Convenient Synthesis of Phosphonate-Containing Compounds. *Tetrahedron Lett.* **2009**, *50* (49), 6745–6747. https://doi.org/10.1016/j.tetlet.2009.09.062.

(8) Srivastav, N. C.; Shakya, N.; Mak, M.; Agrawal, B.; Tyrrell, D. L.; Kumar, R. Antiviral Activity of Various 1-(2′-Deoxy-β-d-Lyxofuranosyl), 1-(2′-Fluoro-β-d-Xylofuranosyl), 1-(3′-Fluoro-β-d-Arabinofuranosyl), and 2′-Fluoro-2′,3′-Didehydro-2′,3′-Dideoxyribose Pyrimidine Nucleoside Analogues against Duck Hepatitis B Virus (DHBV) and Human Hepatitis B Virus (HBV) Replication. *J. Med. Chem.* **2010**, *53* (19), 7156–7166. https://doi.org/10.1021/jm100803c.

(9) Kóšiová, I.; Buděšínský, M.; Panova, N.; Rosenberg, I. Synthesis of Novel Deoxynucleoside S-Methylphosphonic Acids Using S-(Diisopropylphosphonomethyl)Isothiouronium Tosylate, a New Equivalent of Mercaptomethylphosphonate. *Org. Biomol. Chem.* **2011**, *9* (8), 2856–2860. https://doi.org/10.1039/c0ob00738b.

(10) Kostov, O.; Páv, O.; Rosenberg, I. Nucleoside-O-Methyl-(H)-Phosphinates: Novel Monomers for the Synthesis of Methylphosphonate Oligonucleotides Using H-Phosphonate Chemistry. *Curr. Protoc. Nucleic Acid Chem.* **2017**, *70* (1), 4.76.1-4.76.22. https://doi.org/10.1002/cpnc.35.

(11) Kostov, O.; Liboska, R.; Páv, O.; Novák, P.; Rosenberg, I. Solid-Phase Synthesis of Phosphorothioate/ Phosphonothioate and Phosphoramidate/ Phosphonamidate Oligonucleotides. *Molecules* **2019**, *24* (10). https://doi.org/10.3390/molecules24101872.
